# Exosomes mediate LTB_4_ release during neutrophil chemotaxis

**DOI:** 10.1371/journal.pbio.3001271

**Published:** 2021-07-07

**Authors:** Ritankar Majumdar, Aidin Tavakoli Tameh, Subhash B. Arya, Carole A. Parent

**Affiliations:** 1 Laboratory of Cellular and Molecular Biology Center for Cancer Research, NCI, NIH, Bethesda, Maryland, United States of America; 2 Department of Pharmacology, University of Michigan Medical School, Ann Arbor, United States of America; 3 Life Sciences Institute, University of Michigan, Ann Arbor, Michigan, United States of America; 4 Department of Cell and Developmental Biology, University of Michigan Medical School, Ann Arbor, Michigan, United States of America; 5 Rogel Cancer Center, University of Michigan Medical School, Ann Arbor, Michigan, United States of America; National Jewish Medical and Research Center/Howard Hughes Medical Institute, UNITED STATES

## Abstract

Leukotriene B_4_ (LTB_4_) is secreted by chemotactic neutrophils, forming a secondary gradient that amplifies the reach of primary chemoattractants. This strategy increases the recruitment range for neutrophils and is important during inflammation. Here, we show that LTB_4_ and its synthesizing enzymes localize to intracellular multivesicular bodies, which, upon stimulation, release their content as exosomes. Purified exosomes can activate resting neutrophils and elicit chemotactic activity in an LTB_4_ receptor-dependent manner. Inhibition of exosome release leads to loss of directional motility with concomitant loss of LTB_4_ release. Our findings establish that the exosomal pool of LTB_4_ acts in an autocrine fashion to sensitize neutrophils towards the primary chemoattractant, and in a paracrine fashion to mediate the recruitment of neighboring neutrophils in trans. We envision that this mechanism is used by other signals to foster communication between cells in harsh extracellular environments.

## Author’s note

Concerns have emerged about the immunoelectron microscopy results originally reported in the article by Majumdar and colleagues [1]. In addition, errors were made in the scale bars reported in Figs 2H and 3D of the same article. Accordingly, this article has been retracted. We are grateful for the opportunity to republish a version of this article in which the electron microscopy data have been removed. None of the major conclusions attained in the original article are affected by the removal of the contentious data. We sincerely apologize to *PLOS Biology* and the scientific community at large for this occurrence.

## Introduction

Chemotaxis, the directed movement of cells in response to external chemical gradients, is essential to a wide array of biological processes ranging from developmental processes, wound healing, angiogenesis, and immune responses and is implicated in pathological conditions such as chronic inflammatory diseases and metastasis [2]. Upon exposure to endpoint primary chemoattractants, cells secrete secondary chemoattractants that serve to maintain the robustness and sensitivity to the primary chemoattractant signals [3]. Once secreted, these secondary chemoattractants form a gradient to recruit cells that are farther away, thereby dramatically increasing the range and persistence of detection [4]. Intercellular communication through the release of secondary chemoattractants may be homotypic, where the primary and secondary chemoattractant are the same, or it may be heterotypic, where the secondary chemoattractant is different from the primary chemoattractant and is released following stimulation by primary attractants. Homotypic intercellular communication is remarkably exhibited in the social amoebae *Dictyostelium discoideum*, where collective chemotaxis of cells towards cyclic adenosine monophosphate (cAMP) is regulated by the release of cAMP and the formation of characteristic chains of cells called streams [5]. Unlike cAMP in *Dictyostelium*, the release of the secondary chemoattractants CCL3 and CXCL8 by monocytes and dendritic cells in response to the primary chemoattractant Serum Amyloid A represents an example of heterotypic intercellular communication [6]. This ability of chemotaxing cells to transduce autocrine and paracrine chemical signals in response to primary signals has been termed signal relay.

Heterotypic signal relay occurs in neutrophils migrating towards primary chemoattractant gradients through the release of leukotriene B_4_ (LTB_4_) [7]. Following stimulation, cytoplasmic phospholipase A_2_α translocates to the nuclear envelope, where it is poised to hydrolyze membrane bound lipids to form arachidonic acid (AA) [8]. Simultaneously, 5-lipoxygenase (5-LO) is mobilized to the nuclear envelope where it associates with the 5-LO activating protein (FLAP) and acts on AA to generate leukotriene A_4_ (LTA_4)_. LTA_4_, by means of LTA_4_ hydrolase (LTA_4_H), is finally converted into LTB_4_ [8], which is then secreted from cells by an unknown mechanism. Initially thought to be released at the site of infection to mediate neutrophil recruitment and proinflammatory processes [9] [10], LTB_4_ was later shown to be a central secondary chemoattractant in vivo [11]. More recently, we showed that LTB_4_ preferentially mediates neutrophil swarming towards tissue injury sites [12] and established that the secreted LTB_4_ in physiological gradients of primary chemoattractants acts as an amplifier of neutrophil chemotaxis and mediates signal relay between migrating neutrophils [7].

In order for secondary chemoattractants to act as bona fide signal relay molecules, they must be released in a form that enables the generation of stable gradients during chemotaxis. It has been established that the release and subsequent diffusion of LTB_4_ creates extremely transient gradients due to the small size of the molecule [13]. The self-diffusion coefficient of a typical formyl chemoattractant is approximately 10^−5^ cm^2^/s, which is a log order of magnitude higher than typical unsaturated fatty acids [14]. This implies extremely shallow LTB_4_ concentration profiles. Indeed, assessment of the diffusive properties of AA, the structurally similar precursor of LTB_4_, shows shallow and transient gradients compared to a primary chemoattractant such as N-formylMethionyl-Leucyl-Phenylalanine (fMLP) [13]. As a plausible mechanism to generate stable secondary gradients, one may argue in favor of a carrier-based mechanism for passive LTB_4_ transport, such as binding to serum albumin, and this may indeed be true for systemic transport [15]. This, however, would not account for short-range gradients in migrating cells owing to protracted and uncontrolled LTB_4_ release. Moreover, this mechanism would not explain how hydrophobic molecules such as LTB_4_ are protected from the aqueous environment of the extracellular milieu.

The mechanisms by which LTB_4_ is secreted and how LTB_4_ gradients are formed or mediate signal relay of primary chemotactic signals have yet to be determined. However, studies on gradient formation of lipid-modified *Drosophila* morphogens [16], or the formation of palmityolated-Wnt gradients during *Drosophila* embryogenesis [17] and cAMP gradient propagation in *Dictyostelium* [18], point towards vesicular packaging as an effective way of signal dissemination in the extracellular milieu. In the present study, we investigated whether a similar vesicular packaging of LTB_4_ is involved in the creation of a stable gradient during neutrophil chemotaxis. To do so, we assessed whether LTB_4_ is secreted though extracellular vesicles and if its synthesis and transport involve intracellular vesicular trafficking. Most importantly, we also determined if vesicles released during chemotaxis are indeed capable of mediating the LTB_4_-dependent signal relay response during neutrophil chemotaxis.

## Results

### 5-LO translocates to CD63 and LAMP1 positive fractions upon fMLP addition

**Fig 1:**
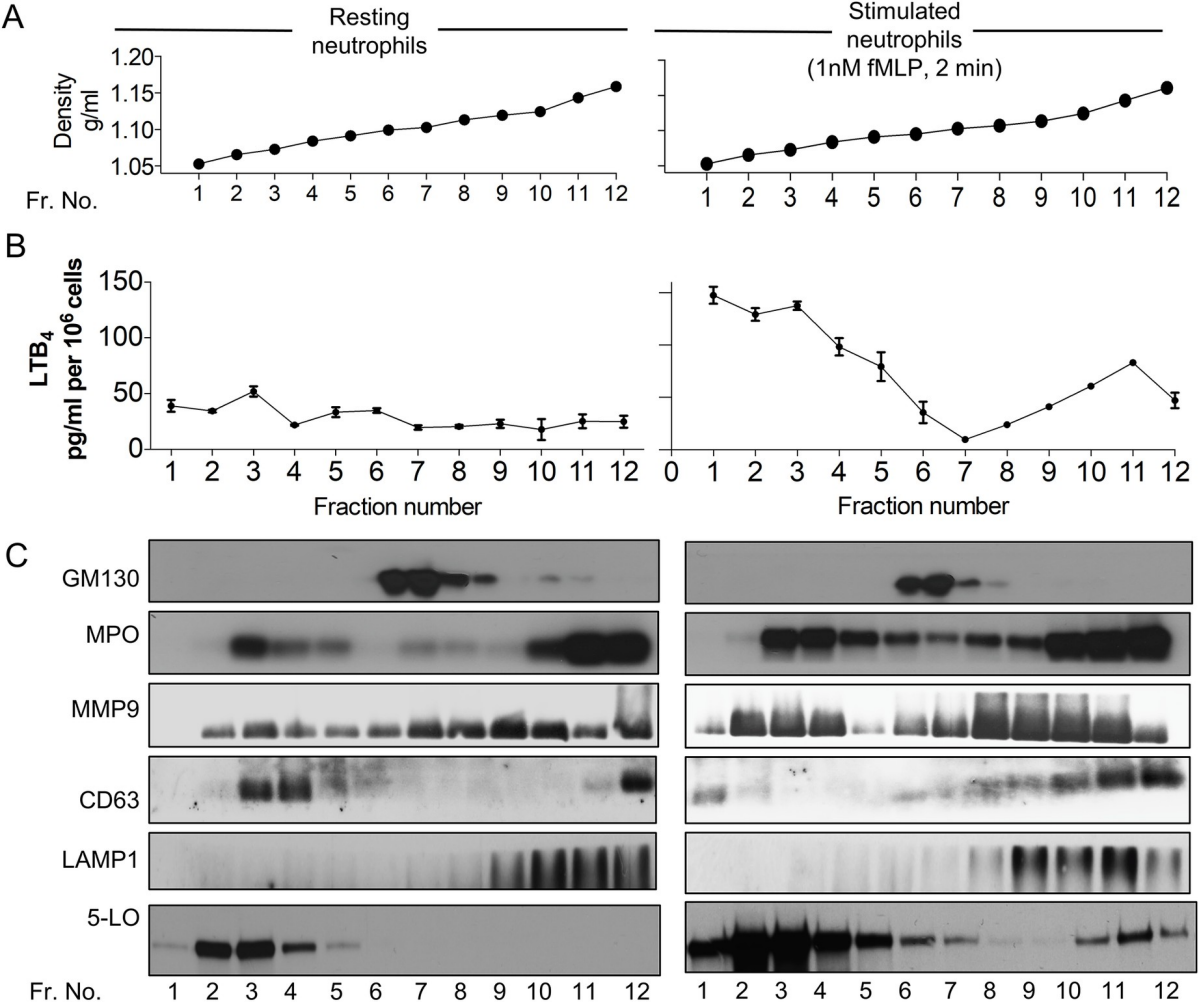
LTB_4_ and 5-LO partition with CD63 and LAMP1 positive fractions in continuous iodixanol gradients. (**A)** Mock-loaded continuous iodixanol gradients were fractionated and the density of each fraction determined as described. (**B)** Nitrogen cavitates of freshly isolated primary human neutrophils were density fractionated on iodixanol gradients, and LTB_4_ content from each fraction was extracted and measured by EIA. **(C)** Total protein from each fraction of iodixanol gradient described in panel B was TCA precipitated, and western analysis was performed using specific antibodies. Data are representative of 2–6 independent experiments. Raw data for panels A and B can be found in the Supporting information section S1 Data file, and uncropped blots for panel C can be found in the S1 Raw images file. fMLP, N-formylMethionyl-Leucyl-Phenylalanine; LTB_4_, Leukotriene B_4_; MMP9, matrix metallopeptidase 9; MPO, myleoperoxidase; TCA, trichloroacetic acid; 5-LO, 5-lipoxygenase.

To begin identifying the mechanisms that underlie LTB_4_ secretion, we measured LTB_4_ content as well as the distribution of 5-LO in resting and activated neutrophils. We fractionated unstimulated and fMLP-stimulated primary human neutrophils using nitrogen cavitation, differential centrifugation, and separation on iodixanol density gradients (Fig 1A). In resting neutrophils, LTB_4_ content was primarily low across the different fractions with a small peak observed in fraction 3 (Fig 1B). On the other hand, in fMLP-stimulated neutrophils, a nonuniform asymmetric increase of LTB_4_ levels was observed, where LTB_4_ levels were elevated in both low- (fractions 1 to 5; density approximately 1.05 to 1.08 g/ml) and high-density fractions (fractions 10 to 12; density approximately 1.17 to 1.19 g/ml), but not in intermediate density fractions (fractions 6 to 9; approximately 1.09 to 1.11 g/ml) that contained the *cis*-Golgi markers GM130 (Fig 1B and 1C). This fMLP-induced asymmetric partitioning of LTB_4_ across different densities was unlike other canonical secretory proteins such as myleoperoxidase (MPO—a marker for the azurophilic granules) and matrix metallopeptidase 9 (MMP9—a marker for gelatinase granules) [19], although total MPO and MMP9 levels appeared to increase poststimulation (Fig 1C). These findings suggest that canonical secretory pathways used by MMP9 and MPO are not involved in LTB_4_ trafficking. On the other hand, fMLP stimulation did induce the redistribution of the tetraspannin CD63 to higher density fractions. As CD63 is known to traffic between late endosomal and secretory compartments such as multivesicular bodies (MVBs) [20], we reasoned that CD63 redistributes to MVBs upon fMLP addition. This was confirmed by the presence of LAMP1, a high-density lysosomal marker shown to be present in MVBs of neutrophils [21], in fractions 9 to 12 (Fig 1C). Remarkably, 5-LO was also found in CD63- and LAMP1-positive high-density fractions upon fMLP stimulation, and the distribution pattern of LTB_4_ was similar to that of 5-LO in both resting and activated conditions, suggesting active 5-LO in this cellular compartment. Together, the presence of LTB_4_ and 5-LO in CD63- and LAMP1-positive fractions suggest the involvement of MVBs in LTB_4_/5-LO transport.

### Neutrophils release exosomes containing LTB_4_ and LTB_4_ synthesizing enzymes upon fMLP addition

**Fig 2:**
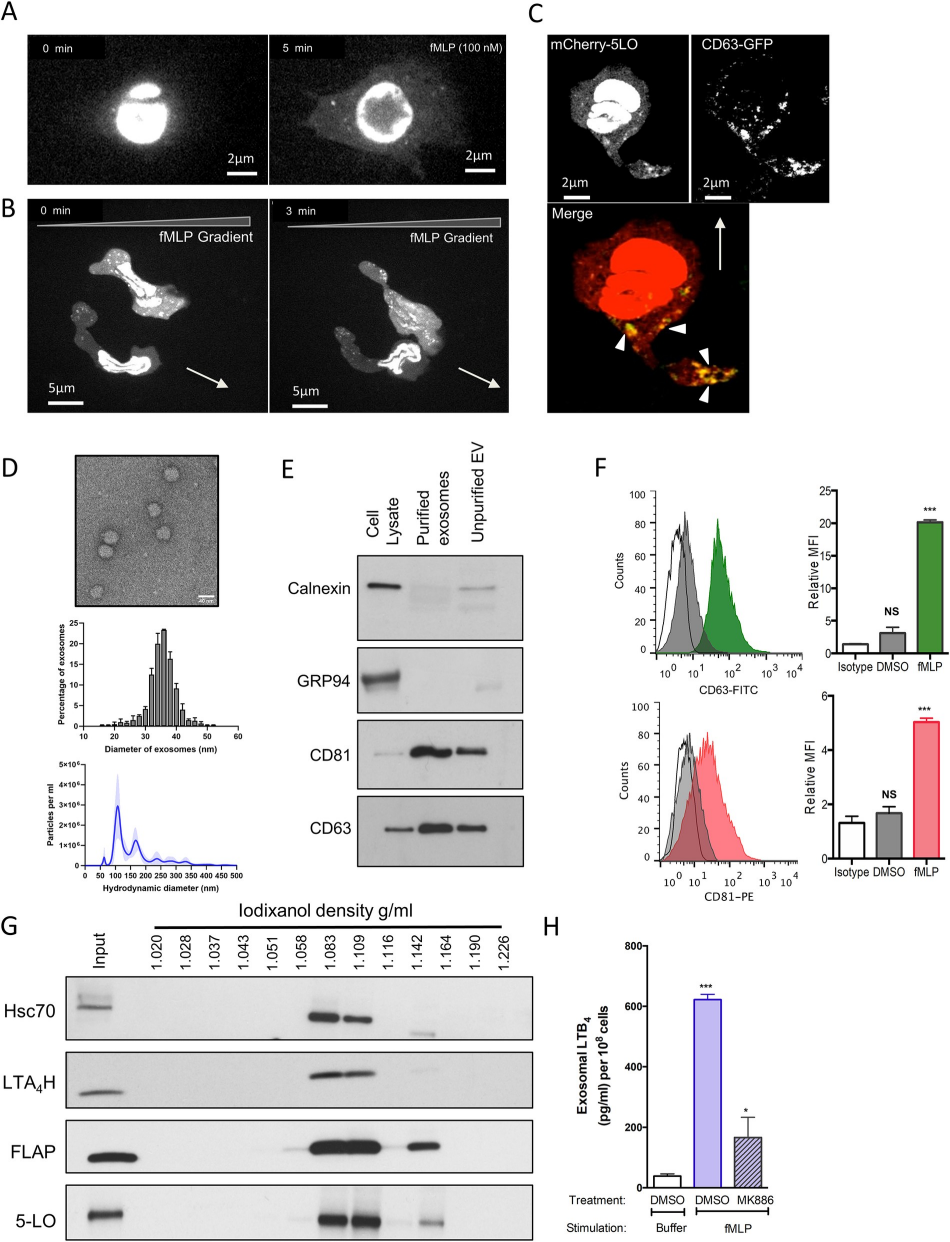
Neutrophils release exosomes containing LTB_4_ and LTB_4_ synthesizing enzymes upon fMLP addition. **(A)** Differentiated mCherry-5LO cells were plated on fibronectin-coated plates and time-lapse images were captured before and after a uniform stimulation with 10 nM fMLP. Fluorescent images are representative of 6 independent experiments. Also see S1 and S2 Movies. **(B)** Differentiated mCherry-5LO cells were allowed to chemotax under agarose towards an fMLP gradient. Time-lapse images were captured 30 min after the addition of fMLP, 700 μm away from the chemoattractant well. Gradient slope approximately 50 pM/μm (see Materials and methods). Fluorescent images are representative of 20 independent experiments. Also see S3 Movie. **(C)** Differentiated CD63-GFP/mCherry-5LO co-expressing cells were allowed to chemotax as described in B. Fluorescent images are representative of 8 independent experiments. Also see S1 Fig and S4 Movie. **(D)** Upper panel: Representative negatively stained EM image of vesicles after purification on a discontinuous iodixanol gradient. Middle panel: Plot depicting the vesicle sizes determined from 600 vesicles from 3 independent experiments (plotted as mean +/− SEM). Bottom panel: Representative nanoparticle tracking analysis of 3 independent experiments (plotted as mean +/− SEM of 5 technical replicates). **(E)** Equal amounts of protein from purified exosomes, crude ultracentrifugation pellets, and total cell lysates (10 μg) were subjected to western analysis using Calnexin, GRP94, CD81, and CD63 specific antibodies. Results are representative of 3 independent experiments. **(F)** Detection of CD63 (upper panel) or CD81 (lower panel) on exosomes obtained from fMLP-stimulated or DMSO-treated neutrophils in a bead-based flow cytometry assay. Relative median fluorescence intensities obtained from 3 independent experiments are shown as mean ± SD. *** and NS indicate *p* < 0.0001 and *p* > 0.05, respectively, compared to the isotype control. Also see S2 Fig. **(G)** Crude ultracentrifugation pellets were loaded on a discontinuous iodixanol gradient, total protein from each fraction precipitated and subjected to western analysis using antibodies for the exosomal marker HSC70 or LTB_4_ synthesizing enzymes. Approximately 5% of the total input was analyzed. Results are representative of 3 independent experiments. **(H)** Purified exosomes from fMLP-stimulated neutrophils treated with DMSO or MK886 (1 μM, 30 min) were lysed and their LTB_4_ content measured using EIA. Results from 4 independent experiments are shown as mean ± SD. *** and NS indicate *p* < 0.0001 and *p* > 0.05, respectively, compared to the LTB_4_ content of exosomes purified from vehicle treated nonstimulated cells. Raw data for panels F and H can be found in the Supporting information section S1 Data file. Uncropped blots for panels E and G can be found in the S1 Raw images file. CD63-GFP, GFP-tagged CD63; EM, electron microscopy; EV, extracellular vesicle; FLAP, 5-LO activating protein; fMLP, N-formylMethionyl-Leucyl-Phenylalanine; LTA_4_H, LTA_4_ hydrolase; LTB_4_, leukotriene B_4_; mCherry-5LO, mCherry-tagged 5-LO; MFI, mean fluorescence intensity; NS, not significant; 5-LO, 5-lipoxygenase.

We next assessed the cellular distribution of 5-LO and CD63 in live chemotaxing cells. We expressed mCherry-tagged 5-LO (mCherry-5LO) and/or GFP-tagged CD63 (CD63-GFP) in the pluripotent hematopoietic cell line PLB-985, which can be differentiated into neutrophil-like cells [22]. We found that 5-LO localizes to the nucleus in resting mCherry-5LO cells (S1A Fig, S1 Movie). However, upon uniform fMLP addition, we readily observed the redistribution of mCherry-5LO to the cytoplasm in a punctate pattern (Fig 2A, S2 Movie). This vesicular localization was even more apparent in cells chemotaxing in a gradient of fMLP and was found to be more prominent at the trailing edge compared to the leading edge of chemotaxing cells (Fig 2B, S3 Movie). The trailing edge localization of 5-LO containing vesicles was further substantiated through phalloidin counterstaining, which marks the leading edge F-actin (S1B Fig). To determine the nature of the 5-LO positive vesicles, we visualized the dynamic distribution of CD63-GFP. As expected, we observed a punctate localization of the MVB marker in both resting (S1A Fig) and stimulated (S1C Fig) cells expressing CD63-GFP. Remarkably, in mCherry-5LO/CD63-GFP co-expressing cells chemotaxing towards fMLP, we observed a clear co-localization of both proteins (Fig 2C, S4 Movie), which again occurred mainly at the trailing edge of the chemotaxing cells.

As we observed co-localization of mCherry-5LO with CD63-GFP, we next set out to determine whether neutrophils secrete exosomes that contain LTB_4_ and LTB_4_ synthesizing enzymes. Supernatants from fMLP-stimulated neutrophils fractionated on a discontinuous gradient of iodixanol yielded a uniform vesicle population with an average inner diameter size of 36.4 nm as measured from electron microscopy images (Fig 2D). The hydrodynamic diameter, determined using nanoparticle tracking analysis, gave rise to a major peak diameter of 108 nm (Fig 2D)—a characteristic size for exosomes [23]. The difference observed in particle size between these 2 methods is likely related to the dehydration that takes place when preparing membrane-bound vesicles for negative staining and electron microscopy. We found that the purified vesicles were enriched in the tetraspannins CD63 and CD81, also reported in exosomes [24], and had very low amounts of the endoplasmic reticulum (ER) integral protein Calnexin or the ER lumen marker GRP94 (Fig 2E). Furthermore, the possibility of contamination from ectosomes and other plasma membrane-derived vesicles was excluded by the absence of the ectosome marker CD11b in purified vesicles [25] (S2A Fig). We also found that the release of exosomes from neutrophils increased upon fMLP addition, as the detection of the exosomal markers CD63 and CD81 increased with fMLP treatment (Fig 2F). This increase was dependent on the concentration of fMLP added to cells (S2B Fig). Moreover, the release of exosomes from cells was also observed following ionomycin addition but not with granulocyte macrophage–colony-stimulating factor (GM–CSF) treatment (S2C and S2D Fig). Most importantly, we determined that the enzymes responsible for the synthesis of LTB_4_, namely 5-LO, FLAP, and LTA_4_H, were all present in fractions containing HSC70 (another known exosome marker [26]) (Fig 2G). The exosomes isolated from fMLP-stimulated neutrophils showed a high LTB_4_ content, which was dramatically reduced by pretreating neutrophils with the FLAP inhibitor MK886 (Fig 2H). Together, these findings show that upon fMLP addition, neutrophils release exosomes containing LTB_4_ and the enzymes required for its synthesis.

### Exosomes activate neutrophils in an LTB_4_-dependent manner

**Fig 3:**
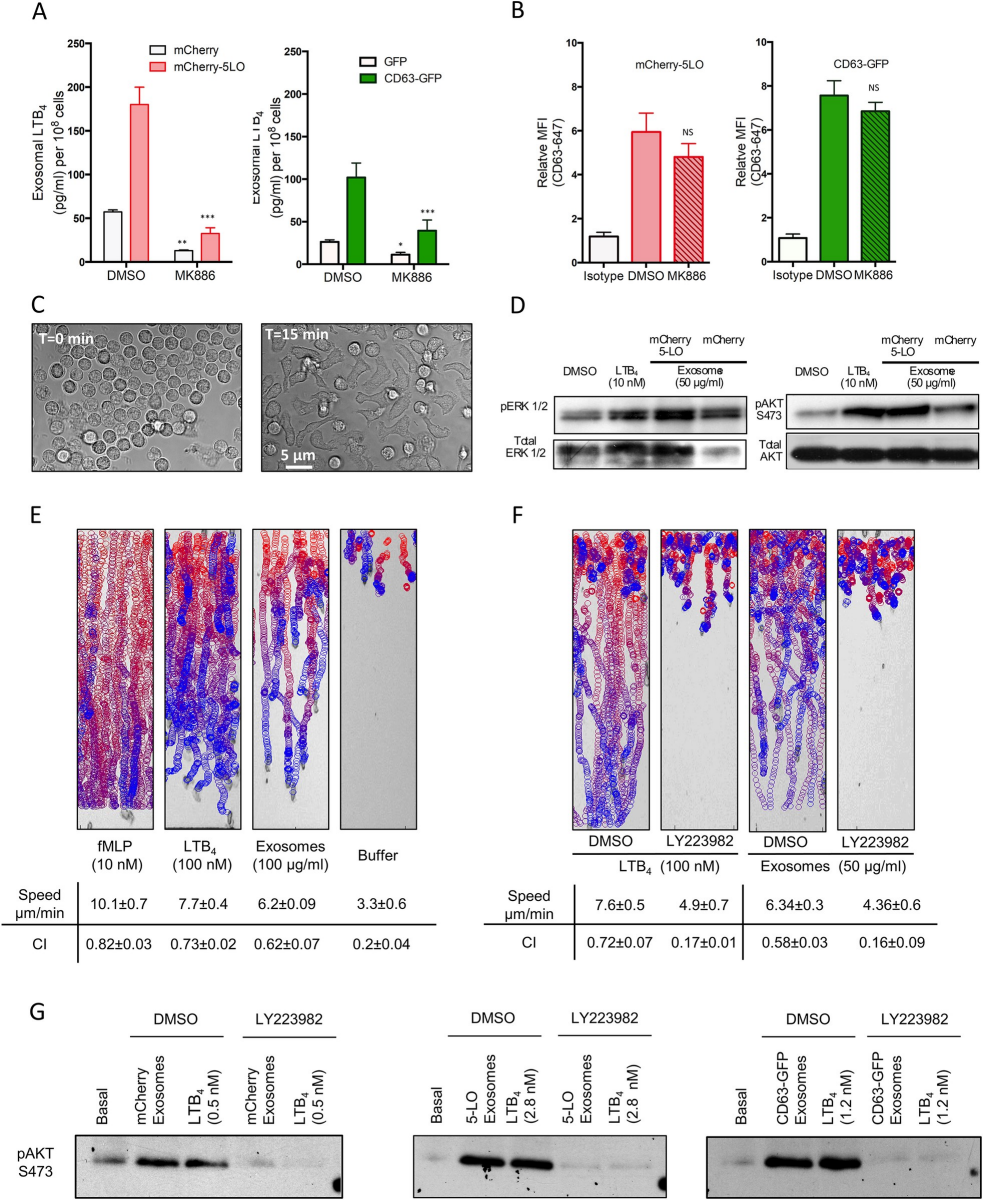
Exosomes activate neutrophils in an LTB_4_-dependent manner. **(A)** Exosomes were purified from differentiated mCherry-5LO or CD63-GFP cells pretreated with either DMSO or MK886 (1 μM, 30 min) and subsequently stimulated with 2 nM fMLP. The exosomes were lysed and their LTB_4_ content measured by EIA. Results from 4 independent experiments are shown as mean ± SD. The symbols ***, **, *, and NS indicate *p* < 0.0001, *p* < 0.001, *p* < 0.01, and *p* > 0.05, respectively, compared to corresponding DMSO-treated controls. **(B)** Detection of CD63 on exosomes purified from differentiated mCherry-5LO or CD63-GFP cells that were pretreated with either DMSO or MK886 (1 μM, 30 min) and subsequently stimulated with 2 nM fMLP in a bead-based flow cytometry assay. Relative median fluorescence intensities obtained from 4 independent experiments are shown as mean ± SD. NS indicates statistical insignificance (*p* > 0.05) of the number of exosomes derived from MK886-treated cells compared to DMSO controls. **(C)** Neutrophil adhesion to fibronectin-coated plates was observed before and 15 min after the addition of 10 μg exosomes derived from CD63-GFP cells. Differential interference contrast images are representative of 3 independent experiments. **(D)** LTB_4_ (10 nM) or exosomes (50 μg/ml) isolated from mCherry or mCherry-5LO cells was added to neutrophils for 15 min, and pAkt S473 and p44/42 MAPK (Erk1/2) (T202/Y204) were determined by western analysis using specific antibodies. Data are representative of 4 independent experiments. See S3A Fig for quantitation. **(E)** EZ-Taxiscan chemotaxis towards fMLP, LTB_4_, exosomes derived from mCherry-5LO expressing cells, or control buffer. The images show paths of individual cells migrating as circles (from red to blue with increasing time) overlaid onto the final frame. Corresponding migration speeds and CI were calculated from 3 different experiments and represented as mean ± SD. Also see S5 Movie. **(F)** Neutrophils were treated with DMSO or LY223982 (10 μM for 30 min) and allowed to migrate towards LTB_4_ or exosomes derived from mCherry-5LO expressing cells. Corresponding migration speeds and CI are calculated from 3 different experiments and represented as mean ± SD. Also see S3B Fig and S6 and S7 Movies. **(G)** Exosomes derived from mCherry, mCherry-5LO, or CD63-GFP expressing cells were divided into 2 parts. One part was used to measure LTB_4_ levels to be added exogenously to neutrophils (control or LY223982 treated). The other part was added to neutrophils and pAkt (S473) levels were assessed after 15 min incubation. Data are representative of 3 independent experiments. See S3C Fig for quantification. Raw data for panels A and B can be found in the Supporting information section S1 Data file. Uncropped blots for panels D and G can be found in the S1 Raw images file. CD63-GFP, GFP-tagged CD63; CI, chemotaxis index; fMLP, N-formylMethionyl-Leucyl-Phenylalanine; LTB_4_, leukotriene B_4_; mCherry-5LO, mCherry-tagged 5-LO; MFI, mean fluorescence intensity; NS, not significant; 5-LO, 5-lipoxygenase.

We next studied the extent by which exosomes mediate the effects of LTB_4_ on neutrophil function. We first purified exosomes from mCherry-5LO and CD63-GFP expressing cells following fMLP treatment in the presence and absence of MK886 and measured their LTB_4_ content. We found that the exogenous expression of either mCherry-5LO or CD63-GFP increases exosomal LTB_4_ (Fig 3A) and, as we observed with neutrophils (Fig 2H), LTB_4_ content was inhibited by pretreatment with MK886 (Fig 3A). Quantification of the released exosomes showed that the decrease in LTB_4_ content in MK886-treated cells was not a result of a decrease in exosome release by these cells (Fig 3B). More importantly, the addition of exosomes derived from mCherry-5LO or CD63-GFP expressing cells to neutrophils rapidly induced cellular polarization and adhesion, indicating that the exosomes readily activate resting neutrophils (Fig 3C). These observations were further strengthened by the increase of both pErk1/2 and pAkt levels upon the exogenous addition of exosomes from mCherry-5LO or mCherry expressing cells to resting neutrophils (Figs 3D and S3A). Of note, the extent by which the exosome preparations increased pErk1/2 and pAkt levels was greater in cells exposed to mCherry-5LO exosomes versus mCherry exosomes (S3A Fig), which could reflect the higher LTB_4_ content of exosomes derived from mCherry-5LO expressing cells, compared to mCherry expressing cells (Fig 3A).

The EZ-Taxiscan microfluidic device was used to assess whether exosomes are capable of inducing a chemotactic response. As seen in Fig 3E and S5 Movie, neutrophils were able to migrate towards exosomes derived from mCherry-5LO expressing cells with speeds and chemotactic indices (CIs) comparable to those observed in the presence of LTB_4_ alone. To determine whether the chemotactic response was mediated by LTB_4_, we exposed neutrophils treated with the LTB_4_ receptor-1 antagonist LY223982 to exosomes. We found that LY223982 treatment dramatically reduced the chemotactic response of neutrophils to both LTB_4_ and exosomes derived from mCherry-5LO expressing cells (Fig 3F, S6 Movie). The antagonist-treated cells, however, did not show migration defects to saturating concentrations of fMLP, showing that LY223982 treatment did not impede chemotaxis due to nonspecific effects (S3B Fig). We also observed that exosomes derived from MK886-treated mCherry-5LO cells displayed weak chemotactic activity compared to exosomes from control treated cells (S7 Movie), further showing that the LTB_4_ present in exosomes is responsible for the chemotactic behavior. These results were confirmed biochemically by assessing the effects of the exogenous addition of LTB_4_ or exosomes on pAkt in neutrophils pretreated with LY223982. As shown in Fig 3G, LTB_4_ or exosome addition gave rise to equal levels of pAkt for each exosome preparation and LTB_4_ amounts used and, most importantly, the pAkt response was blocked by pretreating the cells with LY223982 (see S3C Fig, for quantification). Together, these findings establish the central role of LTB_4_ in exosome-mediated neutrophil activation.

### Reduced exosome production leads to a decrease in exosomal LTB_4_ release and a loss in directional migration

**Fig 4:**
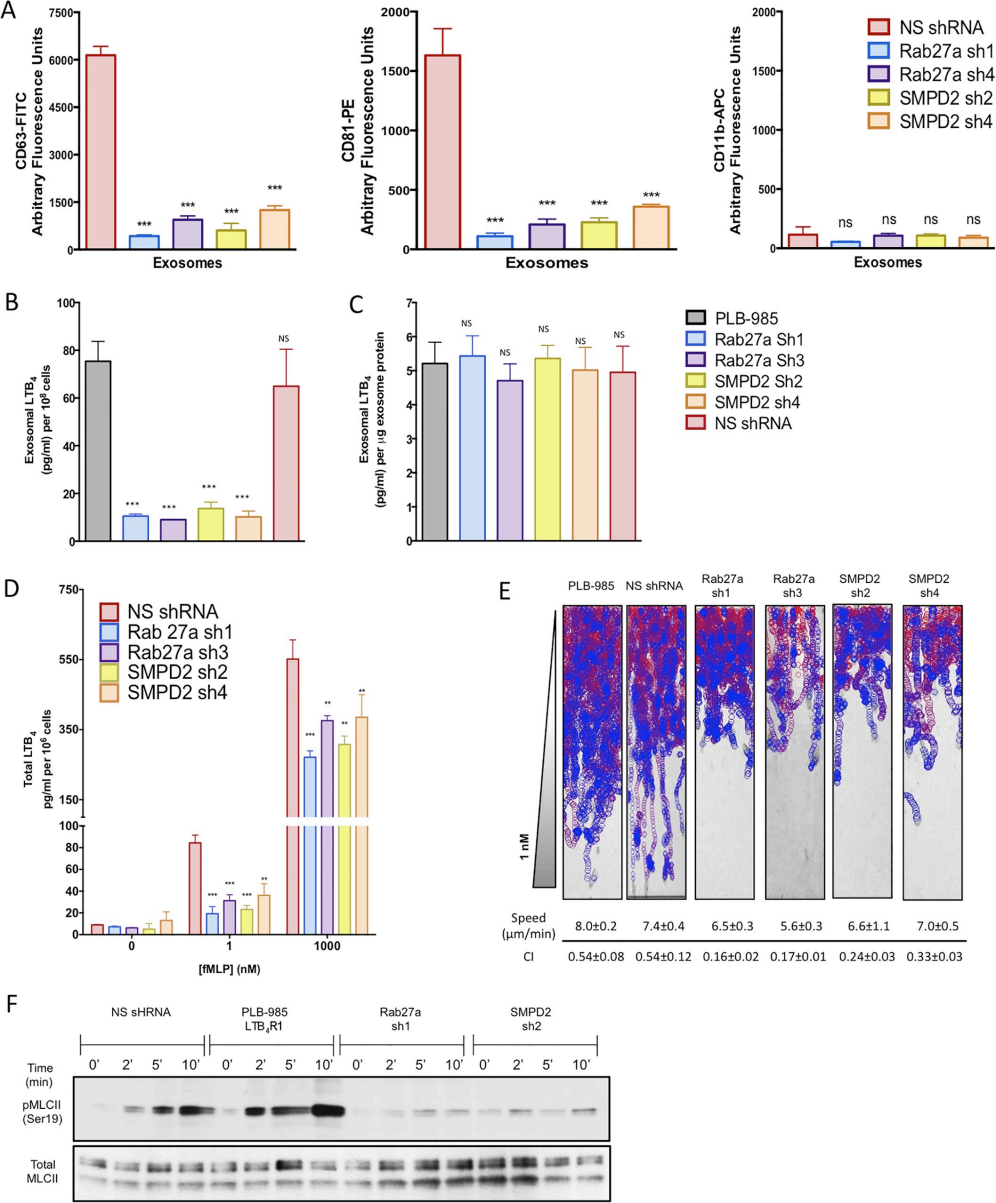
Rab27a and SMPD2 KD cells release less exosomes and show a loss in directional migration. **(A)** Exosomes were purified from differentiated control (NSshRNA), Rab27a shRNA (sh1; sh3), or SMPD2 shRNA (sh2; sh4) KD cells after treatment with fMLP (2 nM, 30 min) and analyzed using a bead-based flow cytometry assay with CD63-FITC, CD81-PE, and CD11b-APC conjugated antibodies. Panels show quantitative analysis from 3 independent experiments as mean ± SD. Evaluation of exosome flow cytometry was done as described in the legend of S2A Fig. See S4B Fig for flow cytometry graphs. **(B, C)** Exosomes were purified from differentiated control and shRNA KD cells stimulated with 2 nM fMLP. The exosomes were lysed and their LTB_4_ content measured by EIA. Results from 3 independent experiments are shown as mean ± SD in pg/ml/10^8^ cells (**B**) or pg/ml/μg of exosome protein (**C**). *** and NS indicate *p* < 0.0001 and *p* > 0.05, respectively, compared to corresponding control PLB-985 cells. **(D)** Differentiated control and shRNA KD cells were stimulated with subsaturating (1 nM) or saturating (1 μM) fMLP for 10 min, and the amount of LTB_4_ in the supernatant was assessed by EIA. Results from 3 independent experiments are shown as mean ± SD. The symbols ** and *** indicate *p* < 0.001 and *p* < 0.0001, respectively, compared to corresponding NSshRNA controls. (**E)** EZ-Taxiscan chemotaxis towards 1 nM of control and KD cell lines. Corresponding migration speeds and CI were calculated from 4 different experiments and represented as mean ± SD. See legend of Fig 3E for details. Also see S8 Movie. (**F)** Differentiated NSshRNA, Rab27a, or SMPD2 KD cells or PLB-985 cells overexpressing LTB_4_R1 were plated on fibronectin-coated plates for 10 min and uniformly stimulated with 1 nM fMLP. At specific time points, samples were subjected to western analyses using an antibody against pMLCII and total MLCII. Data are representative of 3 independent experiments. See S4C Fig for quantification. Raw data for panels A–D can be found in the Supporting information section S1 Data file. Uncropped blots for panel F can be found in the S1 Raw images file. CI, chemotaxis index; fMLP, N-formylMethionyl-Leucyl-Phenylalanine; KD, knockdown; LTB_4_, leukotriene B_4_; MLCII, myosin light chain II; NS, not significant; NSshRNA, nonspecific shRNA; pMLCII, phosphorylated MLCII; shRNA, small hairpin RNA.

To specifically assess the role of exosome formation and release for LTB_4_ secretion, we knocked down Rab27a or neutral sphingomyelinase 1 (nSmase1; SMPD2 gene) using small hairpin RNAs (shRNAs) in PLB-985 cells. Rab27a is critical in MVB docking to the plasma membrane, and its depletion was shown to reduce exosome secretion [27,28]. nSmase is important in exosome secretion by mediating the budding of exosomes into MVBs [29]. We achieved an efficient knockdown (KD) of Rab27a and nSmase1 expression in both undifferentiated and differentiated PLB-985, and the KD of one gene did not alter the expression of the other (S4A Fig). Of the 6 shRNA sequences screened for each gene, we selected Rab27a shRNA 1 (sh1) and Rab27a shRNA 3 (sh3) for further studies (presenting 75% ± 4% and 70% ± 8% reduction of protein levels, respectively), whereas shRNA 2 (sh2) and shRNA 4 (sh4) were selected for SMPD2 (presenting 82% ± 8% and 85% ± 6% reduction in protein levels, respectively). Using CD63 and CD81 as markers, we found that both SMPD2 and Rab27a KD cells show reduced exosome production upon treatment with 2 nM fMLP compared to control nonspecific shRNA (NSshRNA) cells (Figs 4A and S4B). The purity of the exosome preparations was assessed using the ectosome marker CD11b. Similarly, LTB_4_ content of purified exosomes from both KD cell lines was markedly lower than in control cell lines (Fig 4B), although LTB_4_ levels across the different cell types were not different when normalized to the total exosomal protein content (Fig 4C). These findings indicate that depletion of either nSmase1 or Rab27a does not affect LTB_4_ synthesis. When we measured the total amount of LTB_4_ secreted from each cell line in response to 1 nM fMLP, a physiological relevant concentration, we also observed a 75% to 85% reduction in KD cells compared to control (Fig 4D). Interestingly, stimulating cells with a saturating concentration of fMLP (1 μM) reduced total LTB_4_ secretion by 40% to 50%. Although this could result from residual Rab27a or nSmase1 activity in the KD cell lines, nonexosomal sources of LTB_4_ could become dominant under bulk activation conditions.

We next set out to assess the chemotactic behavior of the KD cell lines. We found that KD of either SMPD2 or Rab27a specifically reduced the directional motility (or CI) of cells towards a subsaturating concentration of fMLP (effective concentration 1 nM) (Fig 4E and S8 Movie), as the speed of migration remained unchanged for both KD cell lines (Fig 4E). To further investigate whether exosomes play a role in the relay of primary chemotactic signals, we measured myosin light chain II (MLCII) phosphorylation following a subsaturating fMLP stimulation, a process acutely affected by the disruption of LTB_4_ signaling in neutrophils [7]. As we previously reported, we measured an increase in pMLCII levels in cells expressing NSshRNA in response to 2 nM fMLP (Fig 4F). We also found that the increase of pMLCII levels was further accentuated in PLB-985 cells over expressing the receptor for LTB_4_ (LTB_4_R1), suggesting higher sensitivity of LTB_4_R1 expressing cells towards signal relay processes and the pivotal role that LTB_4_ plays in this response (Fig 4F). In sharp contrast, and as observed in neutrophils where LTB_4_ synthesis is inhibited [7], no fMLP-mediated pMLCII increase was measured in either Rab27a or SMPD2 KD cells (Figs 4F and S4C). Together, these findings show that exosome release regulates LTB_4_ secretion and signal relay during neutrophil chemotaxis.

Importantly, the defects of the KD cells appeared to be highly specific. Both SMPD2 and Rab27a KD cells did not show defects in their ability to adhere upon fMLP stimulation (S5A Fig), nor did they show any defect in their ability to increase pERK1/2 upon fMLP stimulation (S5B Fig). Furthermore, as we found with MK8886 treatment [7], the defects in directional migration were absent when a saturating concentration of fMLP (effective concentration 100 nM) was used (S5C Fig and S9 Movie). Together, these findings indicate that neither Rab27a nor nSmase1 regulate the ability of cells to respond to fMLP and rule out KD specific bystander effects.

### Exosomal LTB_4_ acts in an autocrine and paracrine fashion during neutrophil chemotaxis

**Fig 5:**
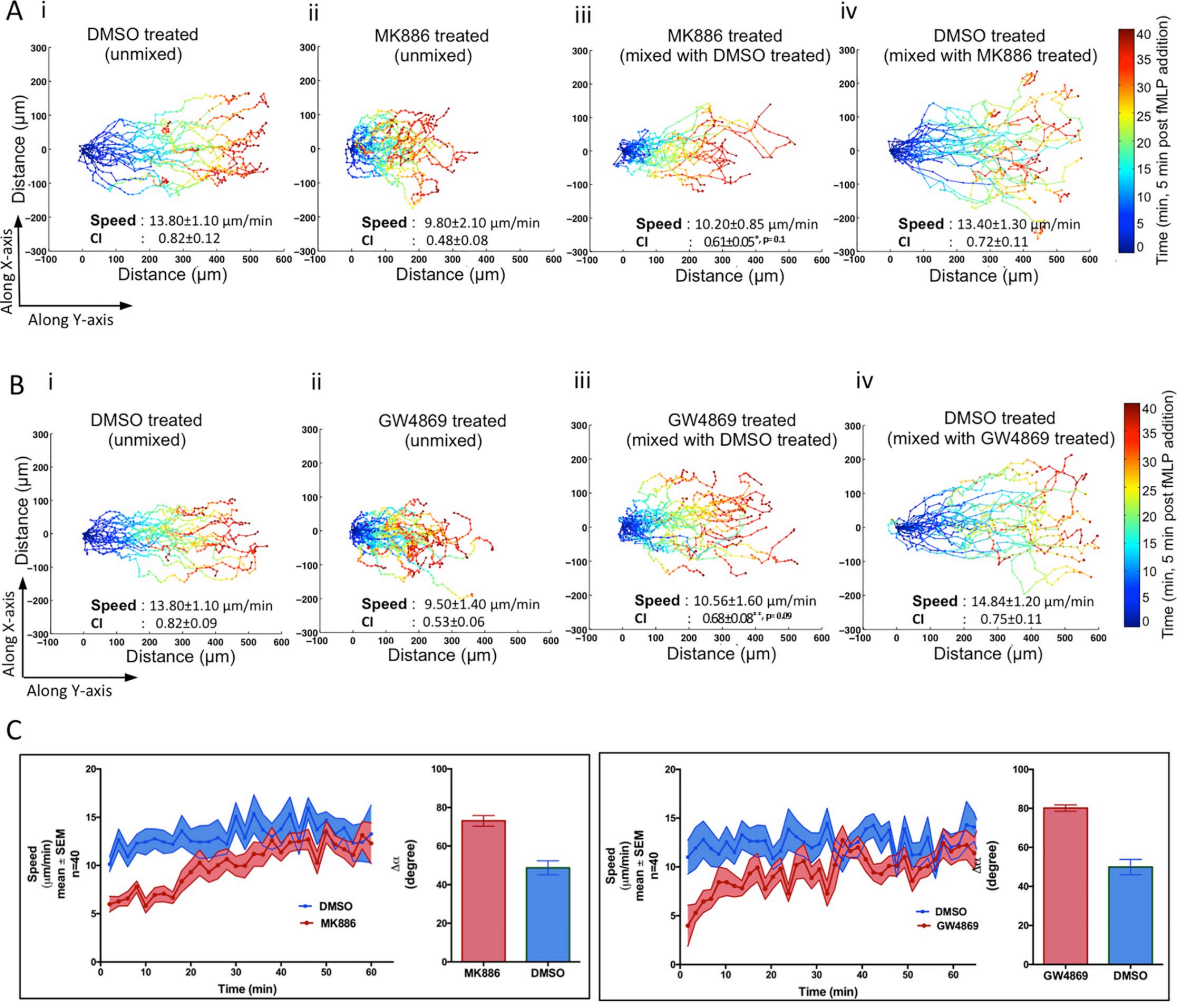
Exosomes mediate the paracrine and autocrine effects of LTB_4_. **(A)** Cell tracks of neutrophils chemotaxing towards an approximately 50 pM/μm gradient of fMLP under agarose (see Materials and methods). **(i)** DMSO-treated cells stained with cytotracker red. **(ii)** MK886-treated cells stained with cytotracker red. **(iii, iv)** MK886-treated cells stained with cytotracker red mixed with DMSO-treated cells stained with cytotracker green: **(iii)** shows the tracks of MK886-treated red cells in the mixture, and **(iv)** shows tracks of DMSO-treated green cells in the mixture. Speed and CI were calculated from the tracks of 40 cells and averaging over 6 independent movies. The temporal location of cells in the X or Y direction is coded according to the color map shown. Also see S10 Movie. **(B)** Cell tracks of neutrophils chemotaxing towards fMLP as described in legends of A. In this case, neutrophils were treated with GW4869. Red cells were tracked in panels i–iii. In panel iv, DMSO-treated cells stained with cytotracker green were tracked. Speed and CI were calculated from the tracks of 40 cells and averaging over 6 independent movies. Also see S6B Fig and S11 Movie. **(C)** Graphs depicting the change in the speed of control- (labeled blue) or inhibitor- (labeled red) treated cells as a function of time. The mean average change in the angular deviation (Δα) between 2 consequent tracks in presented in the bar graph. Data were calculated by from the tracks of 40 cells and averaging over 6 independent movies. *** indicates *p* < 0.0001 compared to DMSO treated cells. Raw data for panels A–C can be found in the Supporting information section S1 Data file. CI, chemotaxis index; fMLP, N-formylMethionyl-Leucyl-Phenylalanine; LTB_4_, leukotriene B_4_.

We next sought to determine whether exosomal LTB_4_ acts in an autocrine and/or paracrine fashion during neutrophil chemotaxis. To do so, we labeled neutrophils with cytotracker red and tracked their movement as they chemotaxed towards fMLP using the under agarose assay. To quantify the data, we color-coded the displacement tracks as a function of the imaging time; blue representing the cell’s position during initial periods of migration and red the final. We found that compared to control cells (Fig 5Ai; S10 Movie), MK886-treated cells migrated shorter distances and with less direction, although speed of migration was not affected (Fig 5Aii; S10 Movie). We also found that treatment with GW4869, an nSmase inhibitor and a known inhibitor of exosome production [29] (S6A Fig), similarly affected chemotactic motion (Fig 5Bi and 5Bii; S11 Movie). Furthermore, and consistent with the chemotaxis defect of SMPD2 KD cells using the EZ-Taxiscan system (Fig 4E), we observed a similar loss in CI and distance migrated in SMPD2 shRNA KD cells (S6B Fig). We then asked if these defects could be rescued by the paracrine action of an exogenous source of exosomes. For this purpose, we mixed untreated neutrophils (labeled green) and treated (MK886 or GW4869) neutrophils (labeled red) in equal proportion and recorded their motility towards fMLP. We observed a dramatic improvement of both directionality and total distance migrated in MK886- (Fig 5Aiii; S10 Movie) and GW4869-treated neutrophils (Fig 5Biii; S11 Movie). No difference was found in the motility of untreated cells labeled either with the green or red dyes (Fig 5Ai, 5Aiv, 5Bi and 5Biv), excluding any dye-specific effects. Furthermore, NSshRNA cells similarly rescued the directionality defect of SMPD2 shRNA KD cells (S6B Fig).

While we observed a dramatic improvement in the CI of GW4869- or MK886-treated cells through the paracrine effects of exosomal LTB_4_ (Fig 5A and 5B), we noticed that the treated cells showed defects in the time required to migrate towards fMLP (Fig 5C). It required up to 35 min for cells treated with either drug to traverse 300 μm, compared with 20 min for untreated cells (Fig 5A and 5B). In addition, the treated cells exhibited slower speeds as well as a loss of directional persistence in the initial phases of migration (Fig 5C). These defects in migration initiation and sensitization to a chemoattractant cue highlight a key role for exosomal LTB_4_ in autocrine signaling.

**Fig 6:**
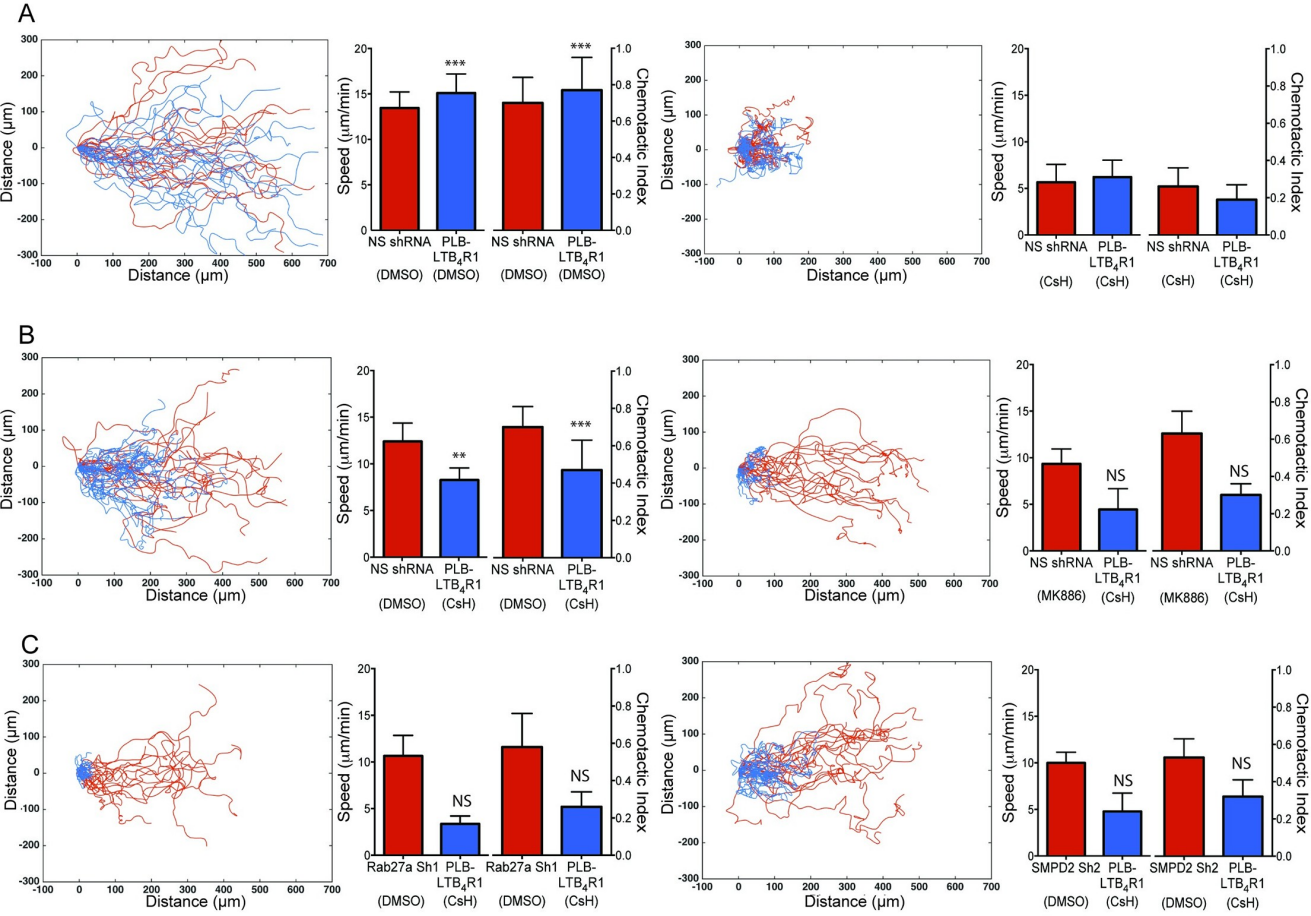
Exosomes mediate signal relay during neutrophil chemotaxis. Tracks, speed, and CI of mixtures of cytotracker-labeled cell lines migrating under agarose towards an approximately 50 pM/μm gradient of fMLP. Also see S7 Fig and S12 Movie. **(A)** Differentiated cells overexpressing the LTB_4_R1 (labeled with cytotracker green, shown as blue tracks) in a mixture with NSshRNA cells (labeled with cytotracker red, shown as red tracks) treated with DMSO (left) or CsH (right). Composite graph shows displacement tracks of both types of cells. Speed and CI were calculated from the tracks of 40 cells and averaging over 8 independent movies**. (B)** Differentiated cells overexpressing the LTB_4_R1 (labeled with cytotracker green, shown as blue tracks) treated with CsH in a mixture with NSshRNA cells (labeled with cytotracker red, shown as red tracks) treated with DMSO (left) or MK866 (right). Composite graph shows displacement tracks of both types of cells. Speed and CI were calculated from the tracks of 40 cells and averaging over 4 independent movies**. (C)** Differentiated cells overexpressing the LTB_4_R1 (labeled with cytotracker green, shown as blue tracks) in a mixture with Rab27a sh1 (labeled with cytotracker red, shown as red tracks) or SMPD2 sh2 (right) cells treated with DMSO. Composite graph shows displacement tracks of both types of cells. Speed and CI were calculated from the tracks of 40 cells and averaging over 4 independent movies. The symbols ***, **, *, and NS indicate *p* < 0.0001, *p* < 0.001, *p* < 0.01, and *p* > 0.05, respectively, compared to CsH-treated PLB-LTB_4_R1 cells. Raw data for panels A–C can be found in the Supporting information section S1 Data file. CI, chemotaxis index; CsH, cyclosporin H; fMLP, N-formylMethionyl-Leucyl-Phenylalanine; LTB_4_R1, receptor for LTB_4_; NS, not significant; NSshRNA, nonspecific shRNA.

One may argue that exosomal LTB_4_ merely increases the robustness of the chemotactic response by regulating the cellular machinery as opposed to acting as a chemotactic beacon. We tested this by mixing cells that cannot detect fMLP with cells defective in exosome release and observed their migration towards fMLP. Due to their higher sensitivity to LTB_4_, we used LTB_4_R1 overexpressing cells as receiving cells in these experiments (Fig 4F). We treated green-labeled LTB_4_R1 cells with the FPR-1 antagonist cyclosporin H (CsH) [30] and mixed them with red-labeled cells expressing control shRNA (NSshRNA), which were also treated with CsH. These cells did not migrate towards fMLP, confirming the efficacy of the antagonist treatment, and no such defects were observed when control labeled cells were mixed (Fig 6A).

However, the motility defect of CsH-treated LTB_4_R1 cells was readily rescued when mixed with untreated NSshRNA cells, but not with MK886-treated NSshRNA cells (Fig 6B). These findings reiterate that LTB_4_ released by NSshRNA cells is responsible for the *trans*-recruitment of CsH-treated cells. Importantly, no rescue in motility was observed in CsH-treated LTB_4_R1 cells when they were mixed with Rab27a or SMPD2 KD cells (Fig 6C) and in GW4869- or MK866-treated neutrophils (S7 Fig). Together, these observations indicate that exosomal LTB_4_ relays chemotactic signals in response to primary attractants during neutrophil chemotaxis.

## Discussion

**Fig 7:**
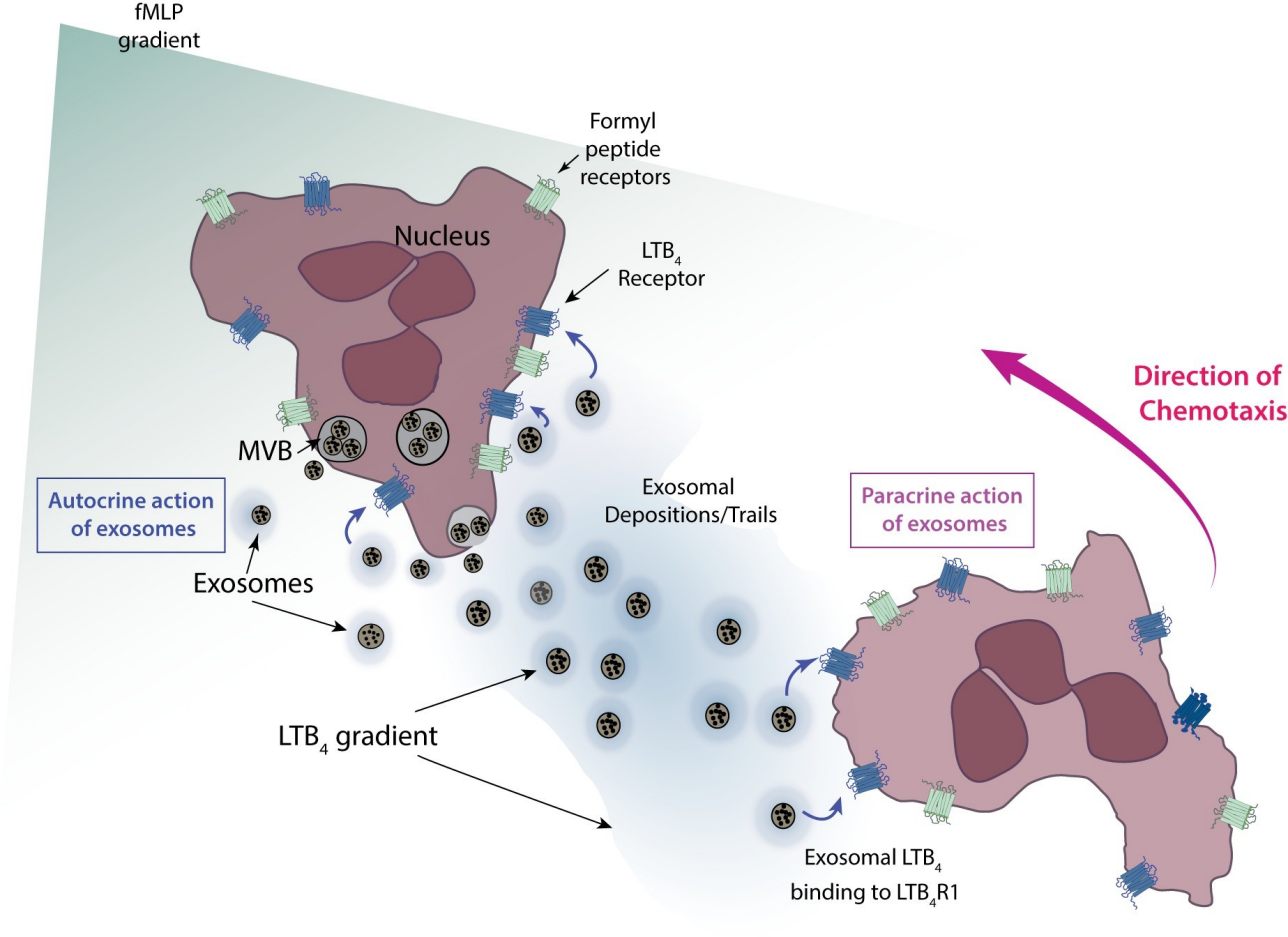
Illustration depicting exosome mediated LTB_4_ relay in neutrophils chemotaxing to fMLP. Cartoon depicting 2 neutrophils chemotaxing toward an fMLP gradient (green). LTB_4_ released from exosomes are shown in blue. See text for details. fMLP, N-formylMethionyl-Leucyl-Phenylalanine; LTB_4_, leukotriene B_4_; LTB_4_R1, receptor for LTB_4_; MVB, multivesicular body.

Our prior studies on neutrophil migrating in chemotactic gradients identified LTB_4_ as an important signal relay molecule that increases the recruitment range of neutrophils to sites of inflammation [7,12]. This observation led us to study the mode of LTB_4_ release from cells and its effective dissemination. In this study, we show that LTB_4_ is packaged in MVBs that are released as exosomes during neutrophil chemotaxis. We present this as a mechanism through which hydrophobic low-diffusible lipid molecules, like LTB_4_, mediate the signal relay process. Our study proposes that neutrophils migrating in primary chemoattractant gradients release exosomes containing LTB_4_ and LTB_4_ synthesizing enzymes. These exosomes subsequently act in an autocrine manner—by sensitizing cells towards the primary chemoattractant—and in a paracrine manner—by acting as molecular beacons for following cells (Fig 7).

We found that both LTB_4_ and 5-LO are present in exosomes, which also contain LTA_4_H and FLAP. The presence of LTB_4_ synthesizing enzymes has previously been reported in exosomes derived from macrophages and dendritic cells [31]. However, unlike dendritic cells that constitutively produce exosomes [32], we detected little or no release of exosomes in unstimulated neutrophils. Instead, we observed exosome release upon treatment with ionomycin as well as following the addition of fMLP, but not GM–CSF, indicating that exosome release is a result of primary stimulation and not a priming event. fMLP is also known to increase the release of plasma membrane–derived secretory vesicles and ectosomes [33] that have considerable overlap with exosomes in terms of size (50 to 200 nm). However, owing to their differences in lipid composition [34], we were able to separate exosomes from ectosomes using iodixanol density gradients. Moreover, the purified exosomes were able to activate resting neutrophils, indicating proinflammatory responses compared to the reported anti-inflammatory responses of ectosomes [33]. Finally, using Rab27a and SMPD2 KD cells, we established that exosomes represent the primary pathway for LTB_4_ release in chemotaxing neutrophils under physiological stimulation conditions. Impaired neutrophil recruitment has been observed in Rab27a knockout (KO) mice under in vivo neutrophil recruitment models [35]. Furthermore, neutrophils deficient in vesicle fusion show less migration in vitro and in an in vivo model of gout [36]. The migration defects we observed in Rab27a and SMPD2 KD cells therefore provide valuable mechanistic insights into the migration defects observed in vivo. Furthermore, the docking of 5-LO containing MVBs at the back of cells and the subsequent release of exosomes are reminiscent of the molecular beacon model first shown in chemotaxing *Dictyostelium* cells [18]. This is similar to recent in vivo observations made by Sung and colleagues [37], where exosome released by HT1080 cells bind to integrins and act as adhesion trails for cellular guidance.

Under uniform stimulation conditions, exosomal LTB_4_ levels range from 2 to 10 nM and are sufficient to elicit neutrophil migration in chemotaxis assays. This, however, may not reflect the actual amount of LTB_4_ being released from exosomes during chemotaxis for the following reasons: (i) exosome release is fMLP dose-dependent and hence a function of the primary chemoattractant gradient; (ii) purified exosomes were prepared by uniformly stimulating neutrophils with subsaturating fMLP concentrations and losses incurred during the purification processes could lead to underestimated amounts of exosomes recovered; (iii) the rate of LTB_4_ release from exosomes and the effective LTB_4_ gradient across and between cells cannot be reliably measured in real time due to the lack of sensitive methodologies; and (iv) the presence of the LTB_4_ synthesizing enzymes, 5-LO, LTA_4_H, and FLAP in neutrophil exosomes along with the presence the primary substrate AA [38], suggest active exosomal LTB_4_ synthesis. Indeed, exosomes isolated from dendritic cells are capable of synthesizing AA metabolites [31]. Although our study elucidates the trafficking and secretion of LTB_4_ during neutrophil chemotaxis, the mechanisms regulating the release of exosomal LTB_4_ remain to be determined. These may include membrane diffusion, passive or active lysis through released neutrophil proteases, or through docking to cell surface heparan sulfate proteoglycans [39]. Alternatively, active LTB_4_ release may be mediated through ABC transporters [40] or other types of active processes. The availability of methods that are sensitive and, most importantly, capable of measuring LTB_4_ in real time and space will help elucidate how LTB_4_ gradients are established and propagated during neutrophil chemotaxis.

Our findings show that exosomal LTB_4_ regulates neutrophil chemotaxis in an autocrine and paracrine fashion. First, we found that Rab27a and SMPD2 KD cells as well as neutrophils treated with an nSmase inhibitor exhibit profound defects in directionality and recruitment range towards fMLP. Similar defects were observed in neutrophils derived from Rab27a KO mice [35] and were primarily attributed to granule exocytosis, a process that is closely related to exosome biogenesis and release. Chemotaxis has not been studied in neutrophils isolated from SMPD2 KO mice, although its role may be inferred through the effects of GW4869 on neutrophil migration. GW4869 did not inhibit neutrophil speed but abrogated directional migration towards fMLP [41], an observation that mirrors our own observation with GW4869 and SMPD2 KD cells. Second, we found that the defects in directionality and recruitment range of SMPD2 KD as well as GW4869-treated cells are rescued by the presence of control cells that provide exosomes to relay signals. One could argue that the rescue was simply due to supplementation of ceramide from the released exosomes and not related to LTB_4_ content [41]. However, we observed a very similar phenotypical recovery by inhibiting LTB_4_ synthesis alone. More importantly, unlike control cells, the KD cells were unable to send a paracrine signal to neutrophils with blocked FPR1, clearly establishing that exosomes represent a critical bearer of the signal relay message.

Exosomes are well-established mediators of intercellular communication [42] and have been known to mediate cell migration in various systems [37]. This work establishes that exosomal communication is extremely efficient and critical in fast moving cells and occurs at a faster rate compared to the slow constitutive release of exosomes reported in the literature [28]. Moreover, our findings add valuable insight into the mechanisms that underlie chronic inflammatory conditions, such as asthma and rheumatoid arthritis [43], as well as lung cancer progression [44], where LTB_4_ plays a central role. We envision that the secretion of other signals that foster communication between cells in harsh extracellular environments are similarly processed through exosome packaging.

## Materials and methods

### Ethics statement

Heparinized whole blood from anonymous healthy human donors was obtained by venipuncture from the NIH Blood Bank Research Program (https://clinicalcenter.nih.gov/blooddonor/donationtypes/lab_research.html) or from the Platelet Pharmacology and Physiology Core at the University of Michigan (IRB#HUM00107120). In both cases, the samples were deidentified, and we did not have access to the HIPAA information.

### Materials

OptiPrep, Histopaque 1077, fMLP, IL8, and LY294002 were obtained from Sigma-Aldrich (St. Louis, Missouri). LTB_4_, MK886, and LY223982 were purchased from Cayman Chemical (Ann Arbor, Michigan). Anti-5-LO, anti-p-Akt (clone C31E5E for S473), anti-AKT, anti-MLCII, anti-pMLCII (Ser19), anti-total ERK1/2, and anti-p-Erk1/2 (clone D13.14.4E) rabbit antibodies were all purchased from Cell Signaling Technology (Beverly, Massachusetts). Anti-MMP9, anti-myeloperoxidase, anti-CD63, anti-LAMP1, and anti-GM130 were obtained from Abcam (Cambridge, Massachusetts). CD11b APC antibody was purchase from BD Biosciences (San Jose, California).

### Isolation of human peripheral blood neutrophils

Heparinized whole blood was obtained by venipuncture from healthy donors. Neutrophils were isolated using dextran sedimentation (3% dextran/0.9% NaCl) coupled to differential centrifugation over Histopaque 1077 [45]. Residual erythrocytes were removed using of hypotonic lysis with 0.2% and 1.6% saline solutions.

### Cell lines

HEK293T cells (ATCC, Manassas, Virginia, United States of America) and Phoenix cells (Orbigen, San Diego, California) were maintained as previously described [46]. For virus packaging, 80% confluent cells were used for transient transfection using Lipofectamine reagent according to manufacturer’s protocol (Life Technologies). PLB-985 cells were maintained in an undifferentiated state and differentiated as described [46]. The status of differentiation was monitored by Mac-1 staining.

### Plasmid constructs and infections of PLB-985 cells

PLB985 cells expressing mCherry-5LO and CD63-GFP as well as co-expressing both CD63-GFP and mCherry-5LO were created using a retroviral approach. Rab27a and SMPD2 KD were achieved using the pGIPZ lentiviral system (GE Dharmacon).

### Constructs and infections of PLB-985 cells

#### Retroviral approach

The mCherry gene was amplified from pCDNA3.1-mCherry, cloned at the 5′ end of the 5-LO cDNA and introduced in pMSCV-Puro. Similarly, eGFP was amplified from pEGFP-N1, cloned at the 3′ end of the CD63 cDNA (kindly donated by Dr. Paul Roche (NIH)), and introduced in pMSCV-Neo. Stable cell lines were created as described [46]. Briefly, expression plasmids were transfected along with the helper plasmid pVSV-G into the packaging Phoenix cell line using Lipofectamine 2000. Transiently produced viruses were harvested after 48 or 72 h. PLB-985 cells were infected with the virus with fresh RPMI1640 culture medium containing 4 μg/ml polybrene and incubated for an additional 48 h. Cells stably expressing the genes were selected in media containing 1.6 mg/ml G-418 or 0.8 μg/ml Puromycin. Stable clonal populations were generated after 14 to 21 d and maintained in the selection media. mCherry-5LO containing pMSCVneo was similarly infected into PLB-985 cells expressing CD63-GFP to create cells co-expressing CD63-GFP and mCherry-5LO.

#### Lentiviral approach

Undifferentiated PLB-985 cells were infected with pGIPZ shRNA lentiviruses (GE Dharmacon) carrying the following hairpin sequence using the manufacturer’s recommendations:

**Table:** 

Accession	shRNA Name	Hairpin
XM_005267109	SMPD2 shRNA1	TGCTGTTGACAGTGAGCGCCGCCGTTGATGTGTGTGCTAATAGTGAAGCCACAGATGTATTAGCACACACATCAACGGCGATGCCTACTGCCTCGGA
XM_005267109	SMPD2 shRNA2	TGCTGTTGACAGTGAGCGCAGGCAACACAATGGTACCCAATAGTGAAGCCACAGATGTATTGGGTACCATTGTGTTGCCTTTGCCTACTGCCTCGGA
NM_003080	SMPD2 shRNA3	TGCTGTTGACAGTGAGCGAGGGGACAGAACTAAAGAACAATAGTGAAGCCACAGATGTATTGTTCTTTAGTTCTGTCCCCCTGCCTACTGCCTCGGA
XM_005267109	SMPD2 shRNA4	TGCTGTTGACAGTGAGCGCCCGCATTGACTACGTGCTTTATAGTGAAGCCACAGATGTATAAAGCACGTAGTCAATGCGGATGCCTACTGCCTCGGA
NM_003080	SMPD2 shRNA 5	TGCTGTTGACAGTGAGCGATGGGTTTTACATCTCCTGTAATAGTGAAGCCACAGATGTATTACAGGAGATGTAAAACCCAGTGCCTACTGCCTCGGA
NM_004580	Rab27a shRNA1	TGCTGTTGACAGTGAGCGCGCTCAATGTCTTTGAGTATTATAGTGAAGCCACAGATGTATAATACTCAAAGACATTGAGCTTGCCTACTGCCTCGGA
NM_183236	Rab27a shRNA2	TGCTGTTGACAGTGAGCGAGCCCAGAGTCTTACATTTAAGTAGTGAAGCCACAGATGTACTTAAATGTAAGACTCTGGGCCTGCCTACTGCCTCGGA
NM_004580	Rab27a shRNA3	TGCTGTTGACAGTGAGCGCGGCTGCAGCTTTATTAGCTTATAGTGAAGCCACAGATGTATAAGCTAATAAAGCTGCAGCCTTGCCTACTGCCTCGGA
XM_005254577	Rab27a shRNA4	TGCTGTTGACAGTGAGCGCCCAGTGTACTTTACCAATATATAGTGAAGCCACAGATGTATATATTGGTAAAGTACACTGGTTGCCTACTGCCTCGGA
NM_183234	Rab27a shRNA5	TGCTGTTGACAGTGAGCGCAGGGAAGACCAGTGTACTTTATAGTGAAGCCACAGATGTATAAAGTACACTGGTCTTCCCTATGCCTACTGCCTCGGA
NM_183236	Rab27a shRNA6	TGCTGTTGACAGTGAGCGAATGCCTCTACGGATCAGTTAATAGTGAAGCCACAGATGTATTAACTGATCCGTAGAGGCATGTGCCTACTGCCTCGGA

Rab27a shRNA1 (sh1), Rab27a shRNA3 (sh3), SMPD2 shRNA2 (sh2), and SMPD2 shRNA 3 (sh4) were selected for further studies based on their ability to KD both mRNA and protein levels of their respective target genes. pGIPZ nonsilencing lentiviral shRNA control was obtained from GE Dhramacon.

### Exosome isolation

A total of 5 × 10^8^ cells were primed with GM–CSF (5 ng/ml; R&D Systems) for 30 min in RPMI without phenol red containing 5% FBS and 10 mM HEPES, centrifuged and resuspended in ice-cold PBS for 30 min to reduce basal exocytosis levels. Cells were harvested and resuspended in modified HBSS (mHBSS) with or without 2 nM fMLP for 30 min at 37°C and centrifuged at 500x*g* for 5 min. To assess the exosome producing ability of various stimulants, unprimed cells were resuspended in basal buffer (25 mM HEPES (pH 7.4), 140 mM NaCl, 4.7 mM KCl, 1.4 mM MgCl_2_, and 10 mM glucose) and stimulated for 30 min with either ionomycin (1 μM) supplemented with CaCl_2_ (2 mM), fMLP (1 μM), GM–CSF (5 ng/ml), or DMSO (0.1%). The supernatant was further centrifuged at 2,000x*g* for 20 min. The resulting supernatants were filtered twice through low protein binding 0.45 μm filters and subsequently centrifuged at 100,000x*g* for 60 min at 4°C. The recovered pellet, containing a heterogeneous mixture of crude vesicles, was resuspended in 500 μl PBS. Further purification of the exosomes was performed as previously reported [47]. Briefly, the resuspended vesicles were loaded onto a discontinuous gradient of iodixanol (40%, 20%, 10%, and 5%, w/v solutions in 0.25 M sucrose/10 mM Tris (pH 7.5)) and centrifuged at 100,000x*g* for 18 h at 4°C. Fractions were collected, diluted in PBS, and centrifuged at 100,000x*g*. The pellet was washed in PBS, and the recovered fractions were used as described.

### Flow cytometric analysis of exosomes

Purified exosomes were incubated with 3.9 μm diameter aldehyde/sulfate latex beads (Life Technologies) for 15 min at room temperature (RT) followed by the addition of 10 μg BSA for an additional 15 min. The incubation volume as increased to 250 μl in PBS and incubated overnight on a turnover rocker. The incubation was stopped the following day by adding glycine to a final concentration of 100 mM. The exosomes-coated beads were washed twice in FACS wash (3% FCS and 0.1% NaN_3_ in PBS) and resuspended in 400 μl FACS wash. The beads were incubated for 30 min with each conjugated primary antibody, washed and analyzed on a FACSCalibur flow cytometer (BD Biosciences). Bead-based flow cytometry was also performed with CD63 antibody-coated dynabeads according to manufacturer’s protocol (Invitrogen).

### Subcellular fractionation through nitrogen cavitation

Freshly isolated neutrophils (50 × 10^6^) were incubated with DFP for 15 min at 37°C, pelleted and resuspended in 1X disruption buffer (DB; 100 mM KCl, 3 mM NaCl, 3.5 mM MgCl_2_, 1.5 mM EGTA, and 10 mM 1,4-piperazinediethanesulfonic acid (PIPES) (pH 7.2), including 1 mM sodium salt of ATP and 0.5 M PMSF) with or without 2 nM fMLP. The reaction was stopped after 2 min by adding an equal volume of ice-cold DB. Cavitation was carried out for 5 min at 500 psi on ice. Cavitates were collected and EGTA was added to the final concentration of 1.5 mM. Lysed cells were centrifuged at 400x*g* for 20 min to remove cell debris and nuclei and 15,000x*g* for 15 min to remove mitochondria. The supernatant was then top loaded onto a 5% to 30% continuous OptiPrep gradient prepared by appropriately diluting a 60% optiprep stock with 3X DB. The gradient was centrifuged at 34,000x*g* for 18.5 h, and 1 ml fractions were collected. Fractionates <1.04 g/l containing primarily cytosolic fraction was discarded and the rest analyzed by immunoblotting after trichloroacetic acid (TCA) precipitation. To determine the density of each fraction, a control OptiPrep gradient containing a blank containing DB was run in parallel. Fractions were collected as described, serially diluted 1:10,000 with water, and the iodixanol concentration determined by absorbance at 244 nm using a molar extinction coefficient of 320 L g^−1^cm^−1^.

### Chemotaxis assays

Chemotaxis was assessed using the under-agarose assay or the EZ-Taxiscan as previously described [46,48].

#### Under-agarose assay

Cell culture dishes were coated with 0.2% gelatin in PBS for 1 h at 37°C. Agarose (0.5%) in 50% PBS and 50% mHBSS was poured and allowed to solidify for 40 min. Three 1 mm diameter wells were carved at 2 mm distance from each other. A chemoattractant was placed in the middle well 15 min before plating neutrophils. The chemoattractant concentration achieved using this method was assessed as described by Afonso and colleagues [7]. Typically, a gradient of 50 pM/μm fMLP was used for under-agarose assays. A total of 5 × 10^5^ neutrophils in 5 μL mHBSS were plated in the outer wells and incubated at 37°C. Cells were allowed to chemotax for 30 min to 2 h depending on the experimental requirement. Cells migrating under agarose were imaged using Axiovert 200 (Carl Zeiss) equipped with a confocal system (Ultraview ERS; PerkinElmer) with a spinning disk head (Yokogawa) Single plane images and z stacks were taken using 63 and 100× plan neofluor objectives (Carl Zeiss).

#### EZ-Taxiscan assay

The EZ-Taxiscan chamber (Effector Cell Institute, Tokyo, Japan) was assembled as described by the manufacturer. Cell migration was recorded every 15 s for 45 min at 37°C in a humidified environmental chamber. Coverslips and chips used in the chamber were coated with 1% bovine serum albumin at RT for 1 h. All glass coverslips were ultrasonicated and washed before use. Cell migration analysis was conducted using MATLAB software as previously described [46].

#### Adhesion assay

Cells (5 × 10^5^ cells/well) were plated on 96-well plates coated with fibronectin, stimulated for 30 min at 37°C, shaken at 2,000 RPM (radius = 1 cm) for 10 s on an orbital shaker, and unbound cells were removed. The remaining cells were fixed (4% PFA) and stained with crystal violet 5 mg/mL in 2% ethanol. Crystal violet was extracted by the addition of 2% SDS, and absorbance was measured at 570 nm.

### Negative stain preparation and electron microscopy

Exosome preparations were prepared for negative stain EM using established methods [49]. In brief, 200-mesh copper grids covered with carbon-coated collodion film (EMS, Hatfield, Pennsylvania, USA) were glow discharged for 30 s at 10 mA in a PELCO easiGlow glow discharge unit (Fresno, California, USA). Aliquots (3.5 μl) of the exosome preparations were adsorbed to the grids and incubated for 1 min at RT. Samples were then washed with 2 drops of water and stained with 2 successive drops of 0.7% (w/v) uranyl formate (EMS, Hatfield, Pennsylvania, USA) followed by blotting until dry. Samples were visualized on a Morgagni 100 kV transmission electron microscope (Thermo Fisher Scientific, Waltham, Massachusetts, USA) at −1.5 μm defocus value.

### Data analysis and diameter measurements

Images were downloaded in JPG format and opened in ImageJ [50]. To perform diameter measurements, a spatial scale of the active image was defined by measuring a 50-nm scale bar on a single micrograph with the *set scale* function. Diameter measurements for each dataset were made using a line tool followed by the *measure* function. A total of 200 measurements were made for each dataset.

### Nanoparticle tracking analysis

The Malvern Nanosight NS300 equipped with 488 nm laser, a high sensitivity sCMOS camera, and syringe pump was used to capture the data for nanoparticle tracking analyses, which were analyzed using the NTA 3.3 Dev Build 3.3.301 software. The purified exosomes were resuspended in 10 mM Tris-Cl (pH 7.4) with 250 mM sucrose, mixed by vortexing, and diluted to 1:100 in particle-free water (0.22 μm filtered) to obtain a concentration within the recommended measurement range (1 to 10 × 10^8^ particles/mL). Using a syringe pump speed of 100/AU, the videos of the particles inflow were captured in script control mode, as 5 videos of 60 s each with 1 s delay and viscosity of water at 25°C. The camera level was kept at 14, capturing 25 frames/s with a total acquisition of 1,500 frames/sample.

### Mixed cell chemotaxis assay, image acquisition, and motility analysis

Cytotracker dyes were added to differentiated PLB-985 cells or freshly isolated neutrophils according to manufacturer’s protocol (Life Technologies), washed and resuspended in mHBSS containing appropriate inhibitors/antagonists. Cells were then washed, and 2.5 × 10^5^ cells of differently labeled cells were mixed. These cells were resuspended in 5 μl mHBSS and loaded onto an under-agarose chamber. Time-lapse imaging was performed using a Plan-Neofluar 10x/0.30 objective (Zeiss Axiovert S100 microscope). Cell tracks were extracted and analyzed using a method developed by McCann and colleagues [51] supplemented by tracking through ImageJ. CI and Δα (persistance) were used to analyze motility behavior. CI was calculated by taking the cosine of the angle between the final displacement vector and the line connecting the chemotactic source to the initial position of the cell. A value of 1 designates perfect chemotaxis. Δα is measured through the angular change between the displacement vector at any given time and the preceding displacement vector. Larger Δα designates lower persistence.

### LTB_4_ measurements

Total LTB_4_ levels were measured using an ELISA kit (R&D Systems). Briefly, neutrophils or PLB-985 cells were resuspended at 1 × 10^6^ cells/ml in PBS and incubated for 30 min on ice. GM–CSF (5 ng/ml; R&D Systems) was added, and cells were further incubated for 1 h at 37°C, centrifuged at 400x*g* for 5 min, resuspended in a volume of 200 μl at 24 × 10^6^ cells/ml in RPMI, and incubated at 37°C until stimulated. The reactions were stopped by adding cold PBS. Cells were centrifuged and LTB_4_ in supernatants were assayed according to manufacturer’s instructions. Exosomal LTB_4_ was measured using the EIA kit according to manufacturer’s protocol (Cayman Chemicals) after disruption by sonication. To measure LTB_4_ in the vesicular fractions of lysed neutrophils, total lipids were first extracted from the different fractions using a method outlined by McColl and colleagues [52]. The extracted lipids were acidified to pH 4.0, loaded onto a C18 SPE column, and eluted with ethyl acetate containing 10% methanol, evaporated under inert nitrogen stream, and resuspended in buffer to obtain a concentrated pool of LTB_4_. LTB_4_ levels were measured using the EIA kit (Cayman Chemicals) according to manufacturer’s recommendations.

### Immunoblotting

Neutrophils were either resuspended in RPMI (1% bovine serum) or plated on fibronectin-coated chambers (for pMLCII) and incubated with inhibitors and diisopropyl fluorophosphates (DFPs) (2 mM) for 10 min at 37°C. Cells were then stimulated with fMLP, collected at specific time points, lysed for 15 min in ice-cold lysis buffer (20 mM Tris (pH 7.5), 10 mN NaCl, 1 mM EDTA, 0.1% NP-40, 3 mM DFP, 2 mM orthovanadate, 10 g/mL aprotinin, 10 g/mL leupeptin, 2.5 mM napyrophosphate, complete protease inhibitor cocktail (Roche Diagnostics, Indianapolis, Indiana)) and processed for SDS-PAGE analyses.

## Supporting information

S1 Fig Characterization of cells expressing CD63-GFP, mCherry-5LO, and CD63/5-LO. (**A)** PLB-985 cells expressing CD63-GFP or mCherry-5LO were plated on fibronectin-coated (50 μg/ml) coverslips, fixed with 4% paraformaldehyde, and imaged in the absence of fMLP. Also see S1 Movie. **(B)** PLB985 cells expressing mCherry-5LO migrating under agarose towards fMLP were fixed with 3.7% paraformaldehyde, 0.1% glutaraldehyde in 0.1 M cacodylate buffer containing 320 mM sucrose, permeablized with 0.2% Triton-X100 for 2 min, and counterstained with Phalloidin FITC. The slope of the gradient is approximately 50 pM/μm, as previously assessed [7]. **(C)** Images of PLB985 cells expressing CD63-GFP migrating under agarose towards fMLP. The slope of the gradient is approximately 50 pM/μm, as previously assessed [7]. Images shown are representative of 6 independent experiments. CD63-GFP, GFP-tagged CD63; fMLP, N-formylMethionyl-Leucyl-Phenylalanine; mCherry-5LO, mCherry-tagged 5-LO.
(PDF)

S2 Fig Characterization of exosomes released from resting and activated neutrophils. **(A)** Exosomes were purified from neutrophils treated with increasing concentrations of fMLP and their surface levels of CD11b assessed by bead-based flow cytometry. Percentage positivity shown is based on the gated exosome fraction derived from nonstimulated cells. Inset: Amount of purified exosomes is quantified by multiplying the percentage positivity of each fraction from 4 independent experiments with corresponding relative median fluorescence intensity values. **(B)** CD81 levels in exosomes purified from neutrophils treated with increasing concentrations of fMLP assessed as mentioned in A. **(C)** CD81 levels in exosomes purified from neutrophils treated with DMSO, Ionomycin, fMLP, and GM–CSF. **(D)** Quantitation of exosome amounts were done as descried in A, using values from 3 independent experiments. Raw data for panels A, B, and D can be found in the Supporting information section S2 Data file. fMLP, N-formylMethionyl-Leucyl-Phenylalanine; GM–CSF, granulocyte macrophage–colony-stimulating factor.
(PDF)

S3 Fig Bioactivity of purified exosomes. **(A)** LTB_4_ (10 nM) or exosomes isolated from PLB-985 cells expressing either mCherry or mCherry-5LO (50 μg/ml) was added to neutrophils for 15 min, and pAkt (S473) and p44/42 MAPK (Erk1/2; T202/Y204) levels were measured using specific antibodies. Quantification of 3 independent experiments is presented as the amount of phosphorylated protein relative to that of DMSO-treated cells (mean ± SD). The amount of pAkt or pErk1/2 at each point was standardized by dividing its value with the value of total Akt or Erk1/2 at the same time point. **(B)** Neutrophils were treated with or without 10 μM LY223982 for 30 min and allowed to migrate towards 1 μM fMLP. Data are representative of 3 independent experiments. See legend of Fig 3E for details. **(C)** Exosomal LTB_4_ (see legends of Fig 3G for details) derived from PLB-985 cells expressing mCherry, mCherry-5LO, or CD63-GFP was added to neutrophils (pretreated or not with LY223982) for 15 min, and pAkt (S473) levels were measured using specific antibodies. Quantification of 3 independent experiments is presented as the amount of pAkt S473 after stimulation relative to that of unstimulated cells (mean ± SD). The amount of pAkt S473 at each time point was standardized by dividing its value with the value of total Akt of the same time point. Raw data for panels A–C can be found in the Supporting information section S2 Data file. CD63-GFP, GFP-tagged CD63; CI, chemotaxis index; fMLP, N-formylMethionyl-Leucyl-Phenylalanine; LTB_4_, leukotriene B_4_; mCherry-5LO, mCherry-tagged 5-LO.
(PDF)

S4 Fig Characterization of Rab27a and SMPD2 KD cells. **(A)** Differentiated and undifferentiated PLB-985 cells were lysed and subjected to western analyses using antibodies specific for Rab27a and nSmase1. GAPDH levels were used as loading controls. Results are representative of 3 independent experiments. **(B)** Exosomes were purified from differentiated control (NSshRNA), Rab27a shRNA (sh1; sh3), or SMPD2 shRNA (sh2; sh4) KD cells after treatment with fMLP (2 nM, 30 min) and analyzed using a bead-based flow cytometry assay with CD63-FITC, CD81-PE, and CD11b-APC conjugated antibodies. See Fig 4A for quantification and additional details. **(C)** Differentiated NSshRNA, Rab27a or SMPD2 KD cells, or PLB-985 cells overexpressing LTB_4_R1 were plated on fibronectin-coated plates for 10 min and uniformly stimulated uniformly with 1 nM fMLP. At specific time points, samples were subjected to western analyses using an antibody against pMLCII and total MLCII. Quantification of 3 independent experiments is presented as the amount of pMLCII after fMLP stimulation relative to that at time 0 (mean ± SD). Raw data for panel C can be found in the Supporting information section S2 Data file. Uncropped blots for panel A can be found in the S1 Raw images file. fMLP, N-formylMethionyl-Leucyl-Phenylalanine; GAPDH, glyceraldehyde 3-phosphate dehydrogenase; KD, knockdown; LTB_4_R1, receptor for LTB_4_; MLCII, myosin light chain II; NSshRNA, nonspecific shRNA; pMLCII, phosphorylated MLCII; shRNA, small hairpin RNA.
(PDF)

S5 Fig Response of Rab27a and SMPD2 KD cells to fMLP. **(A)** Differentiated PLB-985 Rab27a and SMPD2 KD cells were plated on fibronectin-coated (50 μg/ml) plates for 10 min and uniformly stimulated with 1 μM fMLP. The plates were then shaken, and the number of remaining cells attached to the plates was estimated by crystal violet staining. Results represent the percent average ± SD compared to PLB-985 control of 3 independent experiments. **(B)** Differentiated PLB-985, NSshRNA, Rab27a, and SMPD2 KD cells were uniformly stimulated with 100 nM fMLP, and pERK1/2 levels were assessed by immunoblotting. **(C)** EZ-Taxiscan chemotaxis towards 1 μM of control and KD cell lines. Corresponding migration speeds and CI were calculated from 4 different experiments and represented as mean ± SD. See legend of Fig 3E for details. Also see S9 Movie. Raw data for panel A can be found in the Supporting information section S2 Data file. Uncropped blots for panel B can be found in the S1 Raw images file. CI, chemotaxis index; fMLP, N-formylMethionyl-Leucyl-Phenylalanine; GAPDH, glyceraldehyde 3-phosphate dehydrogenase; KD, knockdown; NSshRNA, nonspecific shRNA.
(PDF)

S6 Fig Effect of SMPD2 KD on exosome production and neutrophil chemotaxis. **(A)** Exosomes purified from DMSO- or GW4869-treated neutrophils were incubated with anti-CD63 antibody-coated Dynabeads (Invitrogen) and detected using CD81-PE antibody in a bead-based flow cytometry assay. Inset: Inhibition was quantified using the relative median fluorescence intensity values from 3 independent experiments. **(B)** Representative tracks of chemotaxing differentiated PLB-985 cells under agarose towards a 50-pM/μm gradient of fMLP. Top left: NSshRNA cells stained with cytotracker red. Bottom left: SMPD2 sh1 cells stained with cytotracker red. Top right: NSshRNA cells stained with cytotracker green in a mixture of SMPD2 sh1 and NSshRNA cells. Bottom right: SMPD2 sh1 cells stained with cytotracker red in a mixture of SMPD2 sh1 and NSshRNA cells. The temporal location of cells in the X or Y direction is coded according to the colormap. Also see Fig 5A and 5B for further details. Raw data for panels A and B can be found in the Supporting Information section S2 Data file. fMLP, N-formylMethionyl-Leucyl-Phenylalanine; KD, knockdown; NSshRNA, nonspecific shRNA.
(PDF)

S7 Fig Paracrine action of exosomes on neutrophil chemotaxis. Representative tracks, speed, and CI of mixtures of cytotracker-labeled neutrophils treated with CsH, MK886, or GW4869 migrating under agarose towards an approximately 50 pM/μm gradient of fMLP. Speed and CI were calculated from the tracks of 40 cells and averaging over 4 independent movies. Raw data for panels A and B can be found in the Supporting information section S2 Data file. CI, chemotaxis index; CsH, cyclosporin H; fMLP, N-formylMethionyl-Leucyl-Phenylalanine.
(PDF)

S1 Movie Localization of 5-LO in unstimulated mCherry-5LO cells. Differentiated cells expressing mCherry-5LO were plated on fibronectin-coated coverslips. Images were acquired every 30 s for 18 min. mCherry-5LO, mCherry-tagged 5-LO; 5-LO, 5-lipoxygenase.
(AVI)

S2 Movie Localization of 5-LO in mCherry-5LO cells uniformly stimulated with fMLP. Differentiated PLB-985 cells expressing mCherry-5LO were plated on fibronectin-coated coverslips and uniformly stimulated with 10 nM fMLP. Images were acquired every 30 s for 5 min. fMLP, N-formylMethionyl-Leucyl-Phenylalanine; mCherry-5LO, mCherry-tagged 5-LO; 5-LO, 5-lipoxygenase.
(AVI)

S3 Movie Redistribution of 5-LO in mCherry-5LO cells chemotaxing under agarose towards fMLP. Differentiated PLB-985 cells expressing mCherry-5LO were allowed to chemotax under agarose towards fMLP gradient on a coverslip coated with 0.2% gelatin. Time-lapse images were acquired every 30 s for 5 min, 700 μm away from the chemoattractant well. The movie represents a single Z-slice at 1.5 μm. Gradient slope approximately 50 pM/μm. fMLP, N-formylMethionyl-Leucyl-Phenylalanine; mCherry-5LO, mCherry-tagged 5-LO; 5-LO, 5-lipoxygenase.
(AVI)

S4 Movie Co-localization of mCherry-5LO and CD63-GFP in cells chemotaxing under agarose towards fMLP. Differentiated PLB-985 cells were allowed to chemotax under agarose towards fMLP gradient on a coverslip coated with 0.2% gelatin. Time-lapse images were acquired every 10 s for 4 min, 700 μm away from the chemoattractant well. The movie represents a single Z-slice at 1.5 μm above coverslip. Gradient slope approximately 50 pM/μm. CD63-GFP, GFP-tagged CD63; fMLP, N-formylMethionyl-Leucyl-Phenylalanine; mCherry-5LO, mCherry-tagged 5-LO.
(AVI)

S5 Movie Neutrophil chemotaxis towards fMLP, LTB_4_, or exosomes. EZ-Taxiscan chemotaxis of neutrophils moving towards fMLP (10 nM), LTB_4_ (100 nM), exosomes (100 μg/ml), or a buffer control. Images were acquired every 15 s for 60 min. fMLP, N-formylMethionyl-Leucyl-Phenylalanine; LTB_4_, leukotriene B_4_.
(MP4)

S6 Movie Neutrophil chemotaxis towards LTB_4_ or exosomes in the presence or absence of an LTB_4_R1 antagonist. EZ-Taxiscan chemotaxis of neutrophils moving towards LTB_4_ (100 nM) or exosomes (50 μg/ml). Cells were treated with either DMSO or the LTB_4_R1 antagonist LY223982. Images were acquired every 15 s for 60 min. LTB_4_, leukotriene B_4_; LTB_4_R1, receptor for LTB_4_.
(MP4)

S7 Movie Neutrophils chemotaxis towards exosomes purified from MK886-treated cells. EZ-Taxiscan chemotaxis of neutrophils chemotaxing towards 100 μg/ml exosomes purified from DMSO- or MK886-treated cells expressing mCherry-5LO. Images were acquired every 15 s for 50 min. mCherry-5LO, mCherry-tagged 5-LO.
(MP4)

S8 Movie Chemotaxis of Rab27a and SMPD2 KD cells towards fMLP. EZ-Taxiscan chemotaxis of differentiated PLB-985 NSshRNA, Rab27ash1, Rab27ash3, SMPD2sh2, or SMPD2sh4 cells chemotaxing towards 1 nM fMLP. Images were acquired every 15 s for 30 min. fMLP, N-formylMethionyl-Leucyl-Phenylalanine; KD, knockdown; NSshRNA, nonspecific shRNA.
(MP4)

S9 Movie Chemotaxis of Rab27a and SMPD2 KD cells towards fMLP. EZ-Taxiscan chemotaxis of differentiated PLB-985 NSshRNA, Rab27ash1, Rab27ash3, SMPD2sh2, and SMPD2sh4 cells moving towards 1 μM fMLP. Images were acquired every 15 s for 45 min. fMLP, N-formylMethionyl-Leucyl-Phenylalanine; KD, knockdown; NSshRNA, nonspecific shRNA.
(MP4)

S10 Movie Chemotaxis of MK886-treated neutrophils in the presence of untreated neutrophils. DMSO-treated neutrophils stained with cytotracker red (left panel), MK886-treated neutrophils stained with cytotracker red (middle panel) and mixed populations of DMSO-treated neutrophils stained green and MK886-treated neutrophils stained red (right panel) were allowed to migrate under agarose towards fMLP gradient on a coverslip coated with 0.2% gelatin. Gradient slope approximately 50 pM/μm. Images were acquired every 60 s for 40 min. fMLP, N-formylMethionyl-Leucyl-Phenylalanine.
(MP4)

S11 Movie Chemotaxis of GW4869-treated neutrophils in the presence of untreated neutrophils. DMSO-treated neutrophils stained with cytotracker red (left panel), GW4869-treated neutrophils stained with cytotracker red (middle panel) and mixed populations of DMSO-treated neutrophils stained green and GW4869-treated neutrophils stained red (right panel) were allowed to migrate under agarose towards fMLP gradient on a coverslip coated with 0.2% gelatin. Gradient slope approximately 50 pM/μm. Images were acquired every 60 s for 40 min. fMLP, N-formylMethionyl-Leucyl-Phenylalanine.
(MP4)

S12 Movie Paracrine action of exosomes during neutrophil chemotaxis. Neutrophil stained with cytotracker green and treated with DMSO or CsH were mixed with inhibitor- or control-treated neutrophils stained with cytotracker red. The mixed cells were allowed to chemotax under agarose towards fMLP on a coverslip coated with 0.2% gelatin. Images were acquired every 60 s for 60 min. CsH, cyclosporin H; fMLP, N-formylMethionyl-Leucyl-Phenylalanine.
(MP4)

S1 Data Raw data for all quantitative analyses in the main figures. Raw values for main Figs 1A and 1B, 2F and 2H, 3A and 3B, 4A–4D, 5A–5C, and 6A–6C are presented. The data are shown for each figure panel in a separate worksheet.
(XLSX)

S2 Data Raw data for all quantitative analyses in the Supporting information figures. Raw values for S2A, S2B, S2D, S3A, S3B, S3C, S4C, S5A, S6A and S6B, S7A and S7B Figs are presented. The data are shown for each figure panel in a separate worksheet.
(XLSX)

S1 Raw images Raw images for all western gels in the main and Supporting information figures. Raw images of western gels for Figs 1C, 2E and 2G, 3D and 3G, 4F, and S4A and S5B are presented.
(PDF)

## Abbreviations

AA: arachidonic acid
cAMP: cyclic adenosine monophosphate
CD63-GFP: GFP-tagged CD63
CI: chemotaxis index
CsH: cyclosporin H
ER: endoplasmic reticulum
FLAP: 5-LO activating protein
fMLP: N-formylMethionyl-Leucyl-Phenylalanine
GM–CSF: granulocyte macrophage–colony-stimulating factor
KD: knockdown
KO: knockout
LTA_4_: leukotriene A_4_
LTA_4_H: LTA_4_ hydrolase
LTB_4_: leukotriene B_4_
LTB_4_R1: receptor for LTB_4_
mCherry-5LO: mCherry-tagged 5-LO
MLCII: myosin light chain II
MMP9: matrix metallopeptidase 9
MPO: myleoperoxidase
MVB: multivesicular body
NSshRNA: nonspecific shRNA
shRNA: small hairpin RNA
5-LO: 5-lipoxygenase

## References

[1] Majumdar R, Tavakoli Tameh A, Parent CA. Exosomes Mediate LTB4 Release during Neutrophil Chemotaxis. PLoS Biol. 2016;14(1):e1002336. Epub 2016/01/08. doi: 10.1371/journal.pbio.1002336; PubMed Central PMCID: PMC4704783. 26741884 PMC4704783

[2] Jin T, Xu X, Hereld D. Chemotaxis, chemokine receptors and human disease. Cytokine. 2008;44(1):1–8. doi: 10.1016/j.cyto.2008.06.017 ; PubMed Central PMCID: PMC2613022. 18722135 PMC2613022

[3] Kolaczkowska E, Kubes P. Neutrophil recruitment and function in health and inflammation. Nat Rev Immunol. 2013;13(3):159–75. doi: 10.1038/nri3399 23435331

[4] Majumdar R, Sixt M, Parent CA. New paradigms in the establishment and maintenance of gradients during directed cell migration. Curr Opin Cell Biol. 2014;30(0):33–40. doi: 10.1016/j.ceb.2014.05.010 ; PubMed Central PMCID: PMC4177954. 24959970 PMC4177954

[5] Mahadeo DC, Parent CA. Signal relay during the life cycle of Dictyostelium. Curr Top Dev Biol. 2006;73:115–40. doi: 10.1016/S0070-2153(05)73004-0 . 16782457

[6] Gouwy M, De Buck M, Portner N, Opdenakker G, Proost P, Struyf S, et al. Serum amyloid A chemoattracts immature dendritic cells and indirectly provokes monocyte chemotaxis by induction of cooperating CC and CXC chemokines. Eur J Immunol. 2015;45(1):101–12. doi: 10.1002/eji.201444818 . 25345597

[7] Afonso PV, Janka-Junttila M, Lee YJ, McCann CP, Oliver CM, Aamer KA, et al. LTB4 is a signal-relay molecule during neutrophil chemotaxis. Dev Cell. 2012;22(5):1079–91. doi: 10.1016/j.devcel.2012.02.003 ; PubMed Central PMCID: PMC4141281. 22542839 PMC4141281

[8] Needleman P, Turk J, Jakschik BA, Morrison AR, Lefkowith JB. Arachidonic acid metabolism. Annu Rev Biochem. 1986;55:69–102. doi: 10.1146/annurev.bi.55.070186.000441 . 3017195

[9] Chou RC, Kim ND, Sadik CD, Seung E, Lan Y, Byrne MH, et al. Lipid-cytokine-chemokine cascade drives neutrophil recruitment in a murine model of inflammatory arthritis. Immunity. 2010;33(2):266–78. doi: 10.1016/j.immuni.2010.07.018 ; PubMed Central PMCID: PMC3155777. 20727790 PMC3155777

[10] McDonald B, Kubes P. Chemokines: sirens of neutrophil recruitment-but is it just one song? Immunity. 2010;33(2):148–9. doi: 10.1016/j.immuni.2010.08.006 . 20732637

[11] Oyoshi MK, He R, Li Y, Mondal S, Yoon J, Afshar R, et al. Leukotriene B4-driven neutrophil recruitment to the skin is essential for allergic skin inflammation. Immunity. 2012;37(4):747–58. doi: 10.1016/j.immuni.2012.06.018 ; PubMed Central PMCID: PMC3478399. 23063331 PMC3478399

[12] Lammermann T, Afonso PV, Angermann BR, Wang JM, Kastenmuller W, Parent CA, et al. Neutrophil swarms require LTB4 and integrins at sites of cell death in vivo. Nature. 2013;498(7454):371–5. doi: 10.1038/nature12175 ; PubMed Central PMCID: PMC3879961. 23708969 PMC3879961

[13] Uden AM, Hafstrom I, Palmblad J. Relation to chemotactic factor gradients to neutrophil migration and orientation under agarose. J Leukoc Biol. 1986;39(1):27–35. doi: 10.1002/jlb.39.1.27 . 3001211

[14] Iwahashi M, Kasahara Y, Matsuzawa H, Yagi K, Nomura K, Terauchi H, et al. Self-diffusion, dynamical molecular conformation, and liquid structures of n-saturated and unsaturated fatty acids. J Phys Chem B. 2000;104(26):6186–94. doi: 10.1021/Jp000610l PubMed PMID: WOS:000088057100016.

[15] Zsila F, Bikadi Z, Lockwood SF. In vitro binding of leukotriene B4 (LTB4) to human serum albumin: evidence from spectroscopic, molecular modeling, and competitive displacement studies. Bioorg Med Chem Lett. 2005;15(16):3725–31. doi: 10.1016/j.bmcl.2005.05.125 . 15993588

[16] Greco V, Hannus M, Eaton S. Argosomes: a potential vehicle for the spread of morphogens through epithelia. Cell. 2001;106(5):633–45. doi: 10.1016/s0092-8674(01)00484-6 . 11551510

[17] Entchev EV, Gonzalez-Gaitan MA. Morphogen gradient formation and vesicular trafficking. Traffic. 2002;3(2):98–109. doi: 10.1034/j.1600-0854.2002.030203.x . 11929600

[18] Kriebel PW, Barr VA, Rericha EC, Zhang G, Parent CA. Collective cell migration requires vesicular trafficking for chemoattractant delivery at the trailing edge. J Cell Biol. 2008;183(5):949–61. doi: 10.1083/jcb.200808105 ; PubMed Central PMCID: PMC2592838. 19047467 PMC2592838

[19] Faurschou M, Borregaard N. Neutrophil granules and secretory vesicles in inflammation. Microbes Infect. 2003;5(14):1317–27. doi: 10.1016/j.micinf.2003.09.008 . 14613775

[20] Kobayashi T, Vischer UM, Rosnoblet C, Lebrand C, Lindsay M, Parton RG, et al. The tetraspanin CD63/lamp3 cycles between endocytic and secretory compartments in human endothelial cells. Mol Biol Cell. 2000;11(5):1829–43. doi: 10.1091/mbc.11.5.1829 ; PubMed Central PMCID: PMC14887. 10793155 PMC14887

[21] Cieutat AM, Lobel P, August JT, Kjeldsen L, Sengelov H, Borregaard N, et al. Azurophilic granules of human neutrophilic leukocytes are deficient in lysosome-associated membrane proteins but retain the mannose 6-phosphate recognition marker. Blood. 1998;91(3):1044–58. . 9446668

[22] Tucker KA, Lilly MB, Heck L Jr., Rado TA. Characterization of a new human diploid myeloid leukemia cell line (PLB-985) with granulocytic and monocytic differentiating capacity. Blood. 1987;70(2):372–8. . 3475136

[23] Raposo G, Stoorvogel W. Extracellular vesicles: exosomes, microvesicles, and friends. J Cell Biol. 2013;200(4):373–83. doi: 10.1083/jcb.201211138 ; PubMed Central PMCID: PMC3575529. 23420871 PMC3575529

[24] Andreu Z, Yanez-Mo M. Tetraspanins in extracellular vesicle formation and function. Front Immunol. 2014;5:442. doi: 10.3389/fimmu.2014.00442; PubMed Central PMCID: PMC4165315. 25278937 PMC4165315

[25] Hess C, Sadallah S, Hefti A, Landmann R, Schifferli JA. Ectosomes released by human neutrophils are specialized functional units. J Immunol. 1999;163(8):4564–73. . 10510400

[26] Lancaster GI, Febbraio MA. Exosome-dependent trafficking of HSP70: a novel secretory pathway for cellular stress proteins. J Biol Chem. 2005;280(24):23349–55. doi: 10.1074/jbc.M502017200 . 15826944

[27] Ostrowski M, Carmo NB, Krumeich S, Fanget I, Raposo G, Savina A, et al. Rab27a and Rab27b control different steps of the exosome secretion pathway. Nat Cell Biol. 2010;12(1):19–30; sup pp 1–13. doi: 10.1038/ncb2000 . 19966785

[28] Bobrie A, Krumeich S, Reyal F, Recchi C, Moita LF, Seabra MC, et al. Rab27a supports exosome-dependent and -independent mechanisms that modify the tumor microenvironment and can promote tumor progression. Cancer Res. 2012;72(19):4920–30. doi: 10.1158/0008-5472.CAN-12-0925 . 22865453

[29] Trajkovic K, Hsu C, Chiantia S, Rajendran L, Wenzel D, Wieland F, et al. Ceramide triggers budding of exosome vesicles into multivesicular endosomes. Science. 2008;319(5867):1244–7. doi: 10.1126/science.1153124 . 18309083

[30] Stenfeldt A-L, Karlsson J, Wennerås C, Bylund J, Fu H, Dahlgren C. Cyclosporin H. Boc-MLF and Boc-FLFLF are Antagonists that Preferentially Inhibit Activity Triggered Through the Formyl Peptide Receptor. Inflammation. 2007;30(6):224–9. doi: 10.1007/s10753-007-9040-4 17687636

[31] Esser J, Gehrmann U, D’Alexandri FL, Hidalgo-Estevez AM, Wheelock CE, Scheynius A, et al. Exosomes from human macrophages and dendritic cells contain enzymes for leukotriene biosynthesis and promote granulocyte migration. J Allergy Clin Immunol. 2010;126(5):1032–40, 40 e1-4. doi: 10.1016/j.jaci.2010.06.039 . 20728205

[32] Schorey JS, Cheng Y, Singh PP, Smith VL. Exosomes and other extracellular vesicles in host-pathogen interactions. EMBO Rep. 2015;16(1):24–43. doi: 10.15252/embr.201439363 ; PubMed Central PMCID: PMC4304727. 25488940 PMC4304727

[33] Gasser O, Schifferli JA. Activated polymorphonuclear neutrophils disseminate anti-inflammatory microparticles by ectocytosis. Blood. 2004;104(8):2543–8. doi: 10.1182/blood-2004-01-0361 . 15213101

[34] Sadallah S, Eken C, Schifferli JA. Ectosomes as modulators of inflammation and immunity. Clin Exp Immunol. 2011;163(1):26–32. doi: 10.1111/j.1365-2249.2010.04271.x ; PubMed Central PMCID: PMC3010909. 21039423 PMC3010909

[35] Singh RK, Liao W, Tracey-White D, Recchi C, Tolmachova T, Rankin SM, et al. Rab27a-mediated protease release regulates neutrophil recruitment by allowing uropod detachment. J Cell Sci. 2012;125(Pt 7):1652–6. doi: 10.1242/jcs.100438 ; PubMed Central PMCID: PMC3346826. 22375060 PMC3346826

[36] Colvin RA, Means TK, Diefenbach TJ, Moita LF, Friday RP, Sever S, et al. Synaptotagmin-mediated vesicle fusion regulates cell migration. Nat Immunol. 2010;11(6):495–502. doi: 10.1038/ni.1878 ; PubMed Central PMCID: PMC2951881. 20473299 PMC2951881

[37] Sung BH, Ketova T, Hoshino D, Zijlstra A, Weaver AM. Directional cell movement through tissues is controlled by exosome secretion. Nat Commun. 2015;6:7164. doi: 10.1038/ncomms8164; PubMed Central PMCID: PMC4435734. 25968605 PMC4435734

[38] Subra C, Grand D, Laulagnier K, Stella A, Lambeau G, Paillasse M, et al. Exosomes account for vesicle-mediated transcellular transport of activatable phospholipases and prostaglandins. J Lipid Res. 2010;51(8):2105–20. doi: 10.1194/jlr.M003657 ; PubMed Central PMCID: PMC2903822. 20424270 PMC2903822

[39] Christianson HC, Svensson KJ, van Kuppevelt TH, Li JP, Belting M. Cancer cell exosomes depend on cell-surface heparan sulfate proteoglycans for their internalization and functional activity. Proc Natl Acad Sci U S A. 2013;110(43):17380–5. doi: 10.1073/pnas.1304266110 ; PubMed Central PMCID: PMC3808637. 24101524 PMC3808637

[40] Rius M, Hummel-Eisenbeiss J, Keppler D. ATP-dependent transport of leukotrienes B4 and C4 by the multidrug resistance protein ABCC4 (MRP4). J Pharmacol Exp Ther. 2008;324(1):86–94. doi: 10.1124/jpet.107.131342 . 17959747

[41] Sitrin RG, Sassanella TM, Petty HR. An obligate role for membrane-associated neutral sphingomyelinase activity in orienting chemotactic migration of human neutrophils. Am J Respir Cell Mol Biol. 2011;44(2):205–12. doi: 10.1165/rcmb.2010-0019OC ; PubMed Central PMCID: PMC3049232. 20378749 PMC3049232

[42] Simons M, Raposo G. Exosomes–vesicular carriers for intercellular communication. Curr Opin Cell Biol. 2009;21(4):575–81. doi: 10.1016/j.ceb.2009.03.007 19442504

[43] Sadik CD, Luster AD. Lipid-cytokine-chemokine cascades orchestrate leukocyte recruitment in inflammation. J Leukoc Biol. 2012;91(2):207–15. doi: 10.1189/jlb.0811402 ; PubMed Central PMCID: PMC3290425. 22058421 PMC3290425

[44] Satpathy SR, Jala VR, Bodduluri SR, Krishnan E, Hegde B, Hoyle GW, et al. Crystalline silica-induced leukotriene B4-dependent inflammation promotes lung tumour growth. Nat Commun. 2015;6:7064. doi: 10.1038/ncomms8064; PubMed Central PMCID: PMC4418220. 25923988 PMC4418220

[45] Mahadeo DC, Janka-Junttila M, Smoot RL, Roselova P, Parent CA. A chemoattractant-mediated Gi-coupled pathway activates adenylyl cyclase in human neutrophils. Mol Biol Cell. 2007;18(2):512–22. doi: 10.1091/mbc.e06-05-0418 ; PubMed Central PMCID: PMC1783842. 17135293 PMC1783842

[46] Liu L, Das S, Losert W, Parent CA. mTORC2 regulates neutrophil chemotaxis in a cAMP- and RhoA-dependent fashion. Dev Cell. 2010;19(6):845–57. doi: 10.1016/j.devcel.2010.11.004 ; PubMed Central PMCID: PMC3071587. 21145500 PMC3071587

[47] Tauro BJ, Greening DW, Mathias RA, Ji H, Mathivanan S, Scott AM, et al. Comparison of ultracentrifugation, density gradient separation, and immunoaffinity capture methods for isolating human colon cancer cell line LIM1863-derived exosomes. Methods. 2012;56(2):293–304. doi: 10.1016/j.ymeth.2012.01.002 . 22285593

[48] Comer FI, Parent CA. Phosphoinositide 3-kinase activity controls the chemoattractant-mediated activation and adaptation of adenylyl cyclase. Mol Biol Cell. 2006;17(1):357–66. doi: 10.1091/mbc.e05-08-0781 ; PubMed Central PMCID: PMC1345673. 16267269 PMC1345673

[49] Ohi M, Li Y, Cheng Y, Walz T. Negative Staining and Image Classification—Powerful Tools in Modern Electron Microscopy. Biol Proced Online. 2004;6:23–34. Epub 2004/04/23. doi: 10.1251/bpo70 ; PubMed Central PMCID: PMC389902. 15103397 PMC389902

[50] Schneider CA, Rasband WS, Eliceiri KW. NIH Image to ImageJ: 25 years of image analysis. Nat Methods. 2012;9(7):671–5. Epub 2012/08/30. doi: 10.1038/nmeth.2089 ; PubMed Central PMCID: PMC5554542. 22930834 PMC5554542

[51] McCann CP, Kriebel PW, Parent CA, Losert W. Cell speed, persistence and information transmission during signal relay and collective migration. J Cell Sci. 2010;123(Pt 10):1724–31. doi: 10.1242/jcs.060137 ; PubMed Central PMCID: PMC2864714. 20427323 PMC2864714

[52] McColl SR, Betts WH, Murphy GA, Cleland LG. Determination of 5-lipoxygenase activity in human polymorphonuclear leukocytes using high-performance liquid chromatography. J Chromatogr. 1986;378(2):444–9. doi: 10.1016/s0378-4347(00)80740-9 3016012

