# Supplementary figures and images for: Exosomes mediate LTB4 release during neutrophil chemotaxis
Source: PLoS Biol. 2021 Jul 7;19(7):e3001271. doi: 10.1371/journal.pbio.3001271 (PMC8262914; doi:10.1371/journal.pbio.3001271)

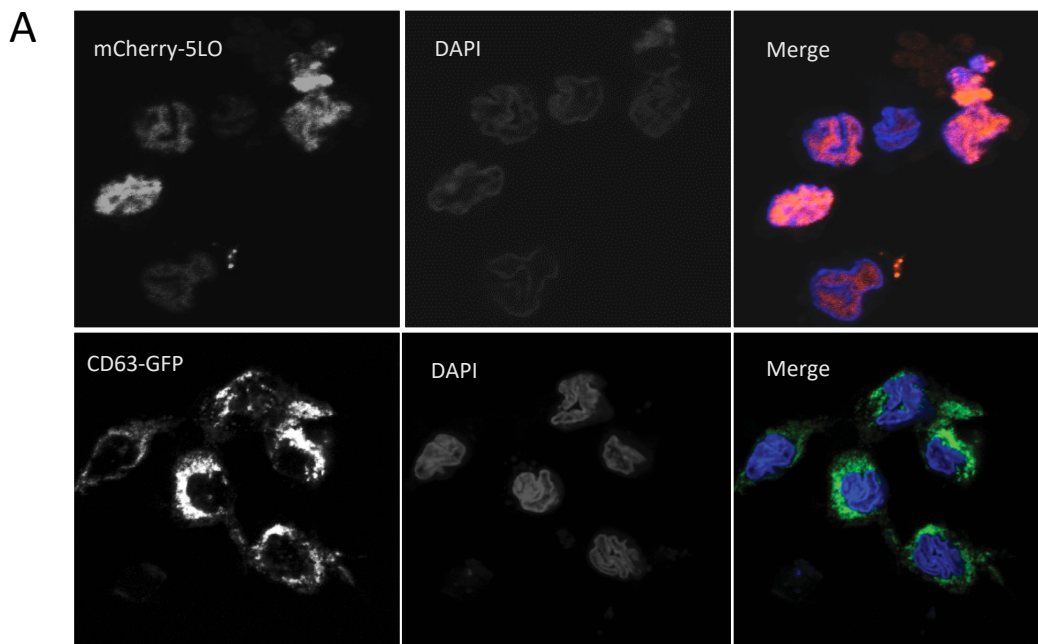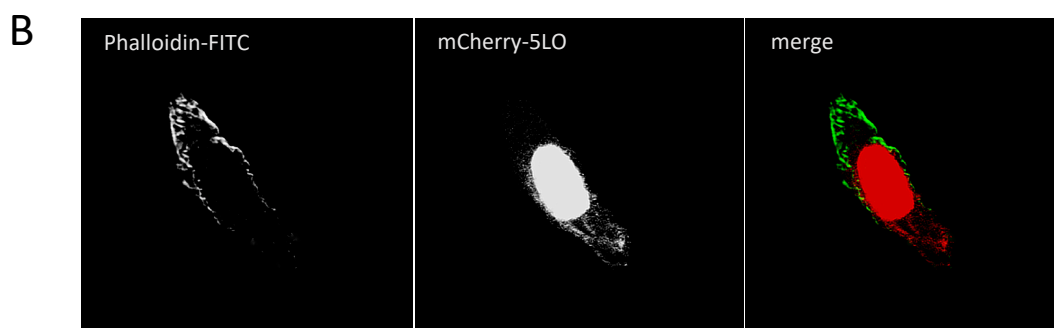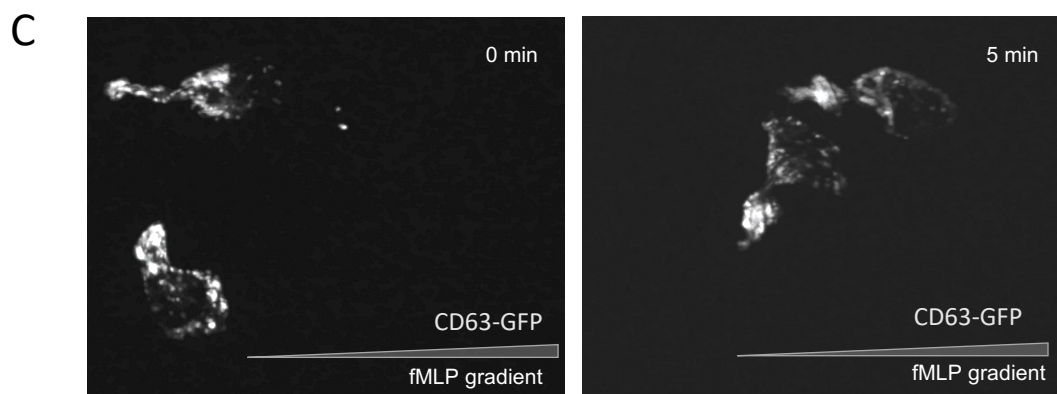

Figure S1

Supplement: S1 Fig — (A) PLB-985 cells expressing CD63-GFP or mCherry-5LO were plated on fibronectin-coated (50 μg/ml) coverslips, fixed with 4% paraformaldehyde, and imaged in the absence of fMLP. Also see S1 Movie. (B) PLB985 cells expressing mCherry-5LO migrating under agarose towards fMLP were fixed with 3.7% paraformaldehyde, 0.1% glutaraldehyde in 0.1 M cacodylate buffer containing 320 mM sucrose, permeablized with 0.2% Triton-X100 for 2 min, and counterstained with Phalloidin FITC. The slope of the gradient is approximately 50 pM/μm, as previously assessed [7]. (C) Images of PLB985 cells expressing CD63-GFP migrating under agarose towards fMLP. The slope of the gradient is approximately 50 pM/μm, as previously assessed [7]. Images shown are representative of 6 independent experiments. CD63-GFP, GFP-tagged CD63; fMLP, N-formylMethionyl-Leucyl-Phenylalanine; mCherry-5LO, mCherry-tagged 5-LO. (PDF) [file pbio.3001271.s001.pdf]

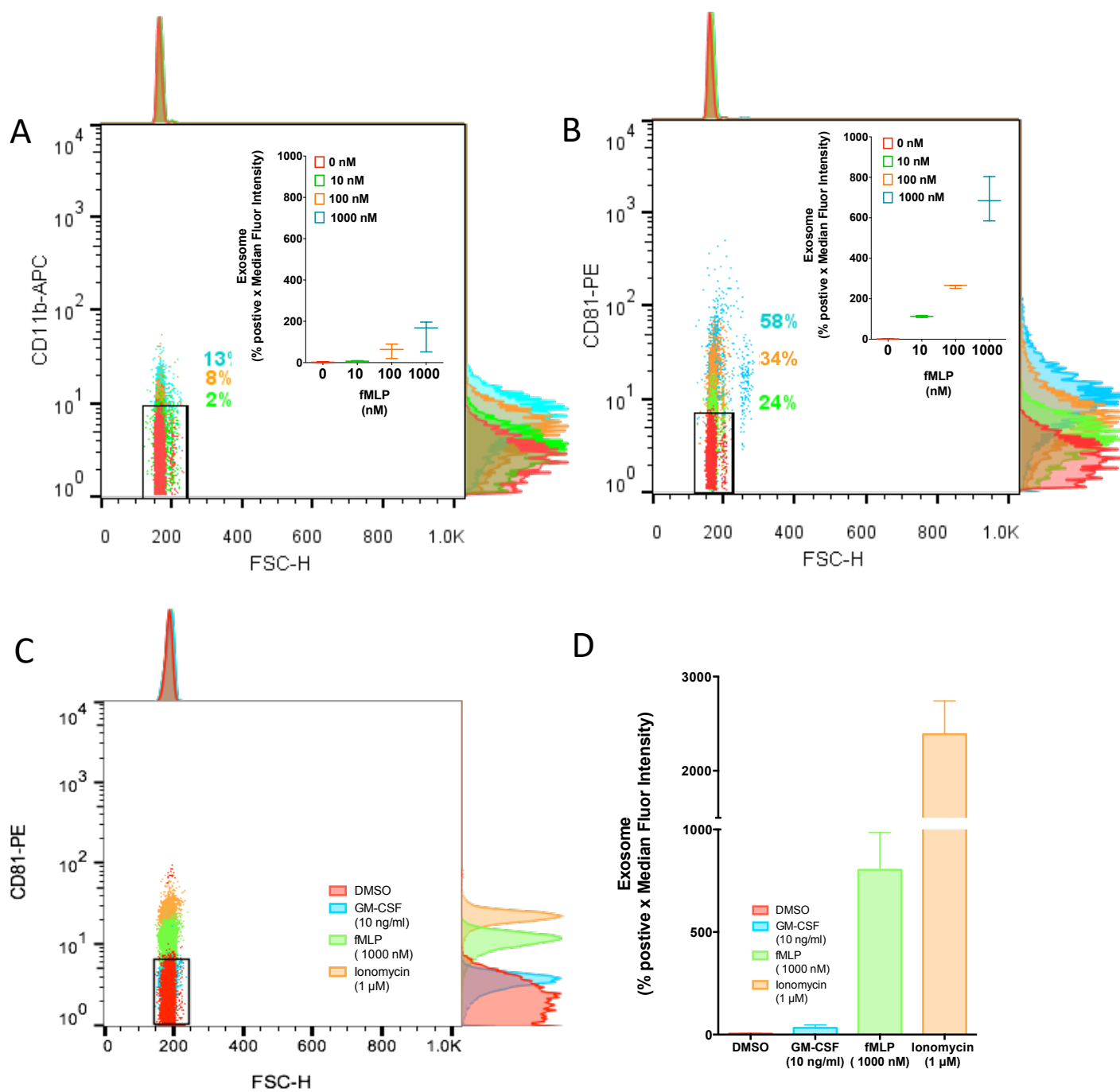

Figure S2

Supplement: S2 Fig — (A) Exosomes were purified from neutrophils treated with increasing concentrations of fMLP and their surface levels of CD11b assessed by bead-based flow cytometry. Percentage positivity shown is based on the gated exosome fraction derived from nonstimulated cells. Inset: Amount of purified exosomes is quantified by multiplying the percentage positivity of each fraction from 4 independent experiments with corresponding relative median fluorescence intensity values. (B) CD81 levels in exosomes purified from neutrophils treated with increasing concentrations of fMLP assessed as mentioned in A. (C) CD81 levels in exosomes purified from neutrophils treated with DMSO, Ionomycin, fMLP, and GM–CSF. (D) Quantitation of exosome amounts were done as descried in A, using values from 3 independent experiments. Raw data for panels A, B, and D can be found in the Supporting information section S2 Data file. fMLP, N-formylMethionyl-Leucyl-Phenylalanine; GM–CSF, granulocyte macrophage–colony-stimulating factor. (PDF) [file pbio.3001271.s002.pdf]

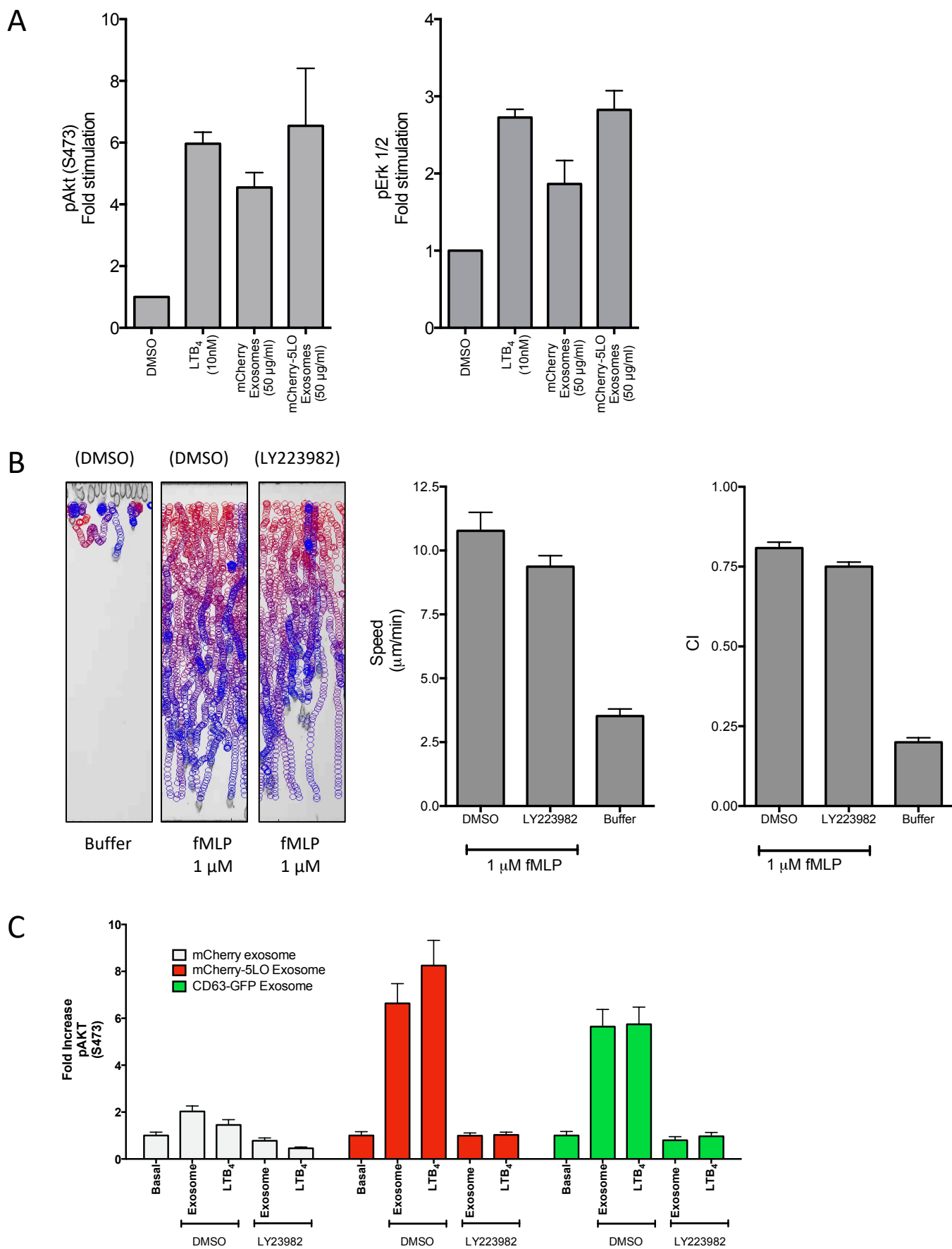

Figure S3

Supplement: S3 Fig — (A) LTB4 (10 nM) or exosomes isolated from PLB-985 cells expressing either mCherry or mCherry-5LO (50 μg/ml) was added to neutrophils for 15 min, and pAkt (S473) and p44/42 MAPK (Erk1/2; T202/Y204) levels were measured using specific antibodies. Quantification of 3 independent experiments is presented as the amount of phosphorylated protein relative to that of DMSO-treated cells (mean ± SD). The amount of pAkt or pErk1/2 at each point was standardized by dividing its value with the value of total Akt or Erk1/2 at the same time point. (B) Neutrophils were treated with or without 10 μM LY223982 for 30 min and allowed to migrate towards 1 μM fMLP. Data are representative of 3 independent experiments. See legend of Fig 3E for details. (C) Exosomal LTB4 (see legends of Fig 3G for details) derived from PLB-985 cells expressing mCherry, mCherry-5LO, or CD63-GFP was added to neutrophils (pretreated or not with LY223982) for 15 min, and pAkt (S473) levels were measured using specific antibodies. Quantification of 3 independent experiments is presented as the amount of pAkt S473 after stimulation relative to that of unstimulated cells (mean ± SD). The amount of pAkt S473 at each time point was standardized by dividing its value with the value of total Akt of the same time point. Raw data for panels A–C can be found in the Supporting information section S2 Data file. CD63-GFP, GFP-tagged CD63; CI, chemotaxis index; fMLP, N-formylMethionyl-Leucyl-Phenylalanine; LTB4, leukotriene B4; mCherry-5LO, mCherry-tagged 5-LO. (PDF) [file pbio.3001271.s003.pdf]

**A**

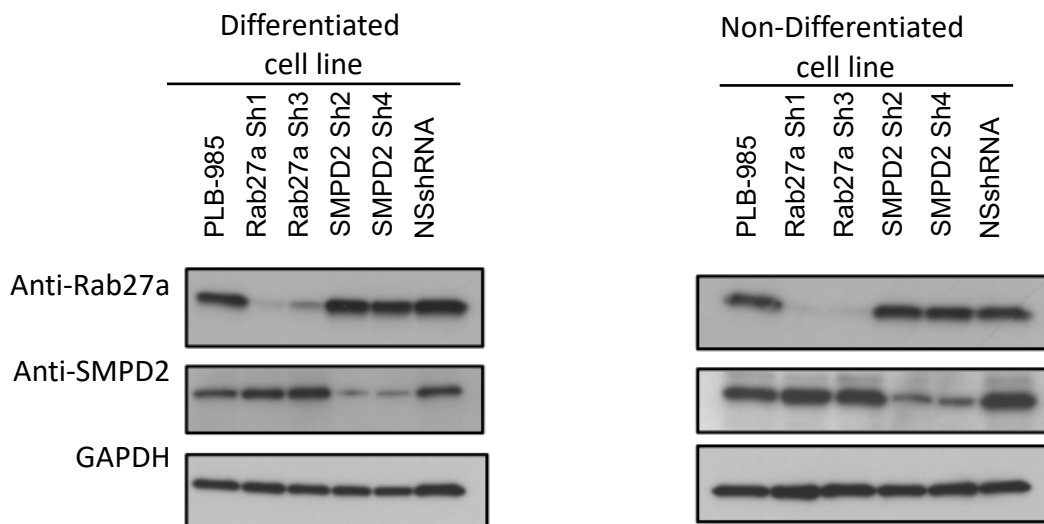

**B**

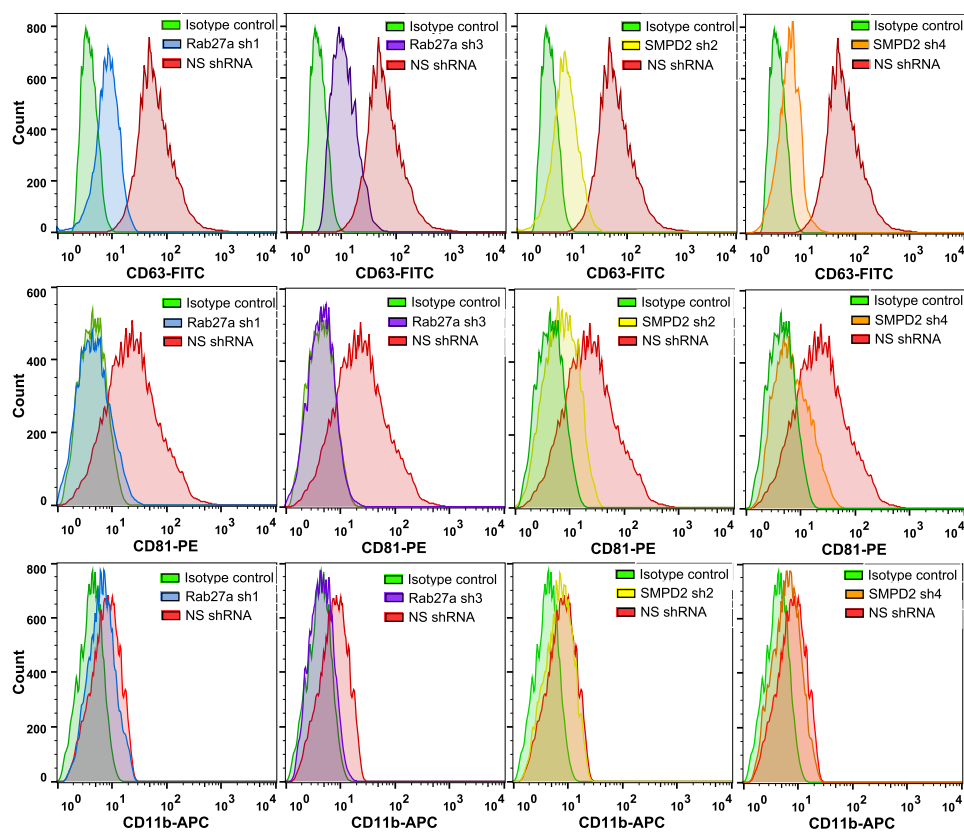

**C**

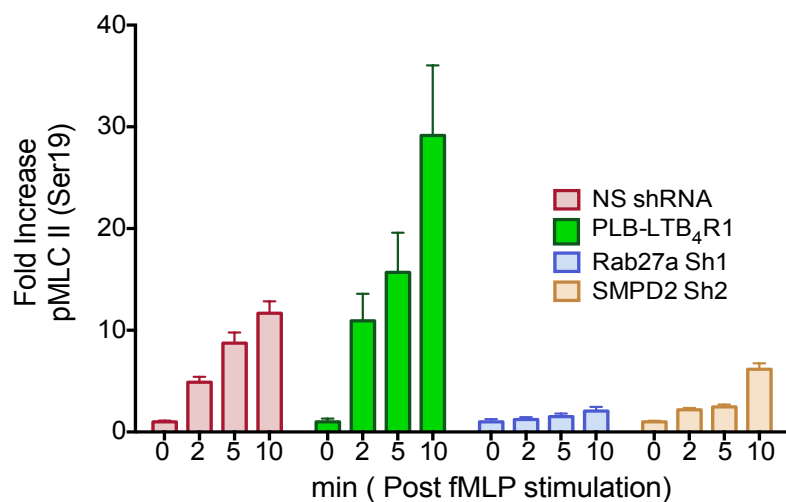

Figure S4

Supplement: S4 Fig — (A) Differentiated and undifferentiated PLB-985 cells were lysed and subjected to western analyses using antibodies specific for Rab27a and nSmase1. GAPDH levels were used as loading controls. Results are representative of 3 independent experiments. (B) Exosomes were purified from differentiated control (NSshRNA), Rab27a shRNA (sh1; sh3), or SMPD2 shRNA (sh2; sh4) KD cells after treatment with fMLP (2 nM, 30 min) and analyzed using a bead-based flow cytometry assay with CD63-FITC, CD81-PE, and CD11b-APC conjugated antibodies. See Fig 4A for quantification and additional details. (C) Differentiated NSshRNA, Rab27a or SMPD2 KD cells, or PLB-985 cells overexpressing LTB4R1 were plated on fibronectin-coated plates for 10 min and uniformly stimulated uniformly with 1 nM fMLP. At specific time points, samples were subjected to western analyses using an antibody against pMLCII and total MLCII. Quantification of 3 independent experiments is presented as the amount of pMLCII after fMLP stimulation relative to that at time 0 (mean ± SD). Raw data for panel C can be found in the Supporting information section S2 Data file. Uncropped blots for panel A can be found in the S1 Raw images file. fMLP, N-formylMethionyl-Leucyl-Phenylalanine; GAPDH, glyceraldehyde 3-phosphate dehydrogenase; KD, knockdown; LTB4R1, receptor for LTB4; MLCII, myosin light chain II; NSshRNA, nonspecific shRNA; pMLCII, phosphorylated MLCII; shRNA, small hairpin RNA. (PDF) [file pbio.3001271.s004.pdf]

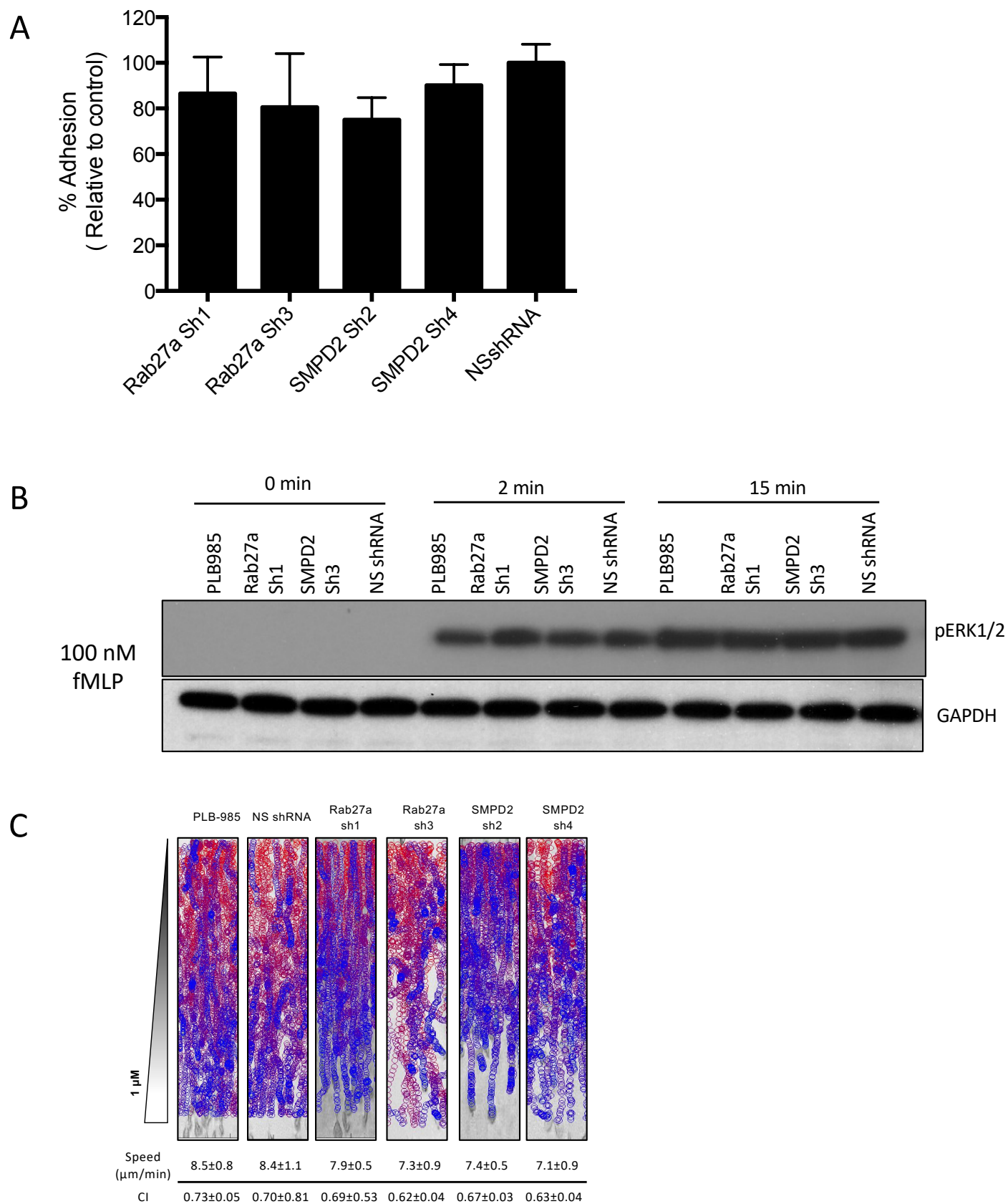

Fig. S5

Supplement: S5 Fig — (A) Differentiated PLB-985 Rab27a and SMPD2 KD cells were plated on fibronectin-coated (50 μg/ml) plates for 10 min and uniformly stimulated with 1 μM fMLP. The plates were then shaken, and the number of remaining cells attached to the plates was estimated by crystal violet staining. Results represent the percent average ± SD compared to PLB-985 control of 3 independent experiments. (B) Differentiated PLB-985, NSshRNA, Rab27a, and SMPD2 KD cells were uniformly stimulated with 100 nM fMLP, and pERK1/2 levels were assessed by immunoblotting. (C) EZ-Taxiscan chemotaxis towards 1 μM of control and KD cell lines. Corresponding migration speeds and CI were calculated from 4 different experiments and represented as mean ± SD. See legend of Fig 3E for details. Also see S9 Movie. Raw data for panel A can be found in the Supporting information section S2 Data file. Uncropped blots for panel B can be found in the S1 Raw images file. CI, chemotaxis index; fMLP, N-formylMethionyl-Leucyl-Phenylalanine; GAPDH, glyceraldehyde 3-phosphate dehydrogenase; KD, knockdown; NSshRNA, nonspecific shRNA. (PDF) [file pbio.3001271.s005.pdf]

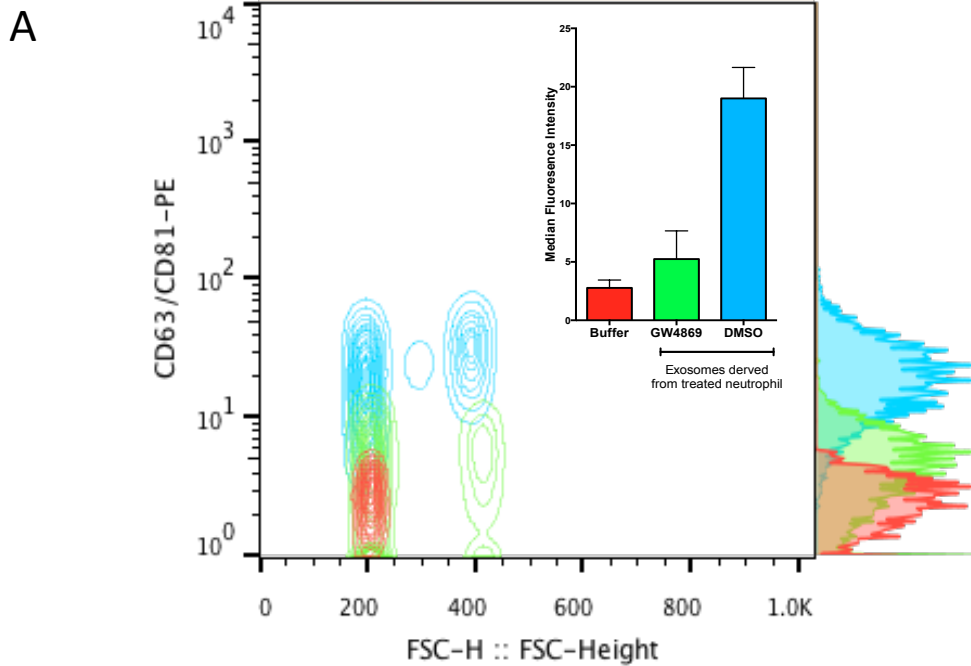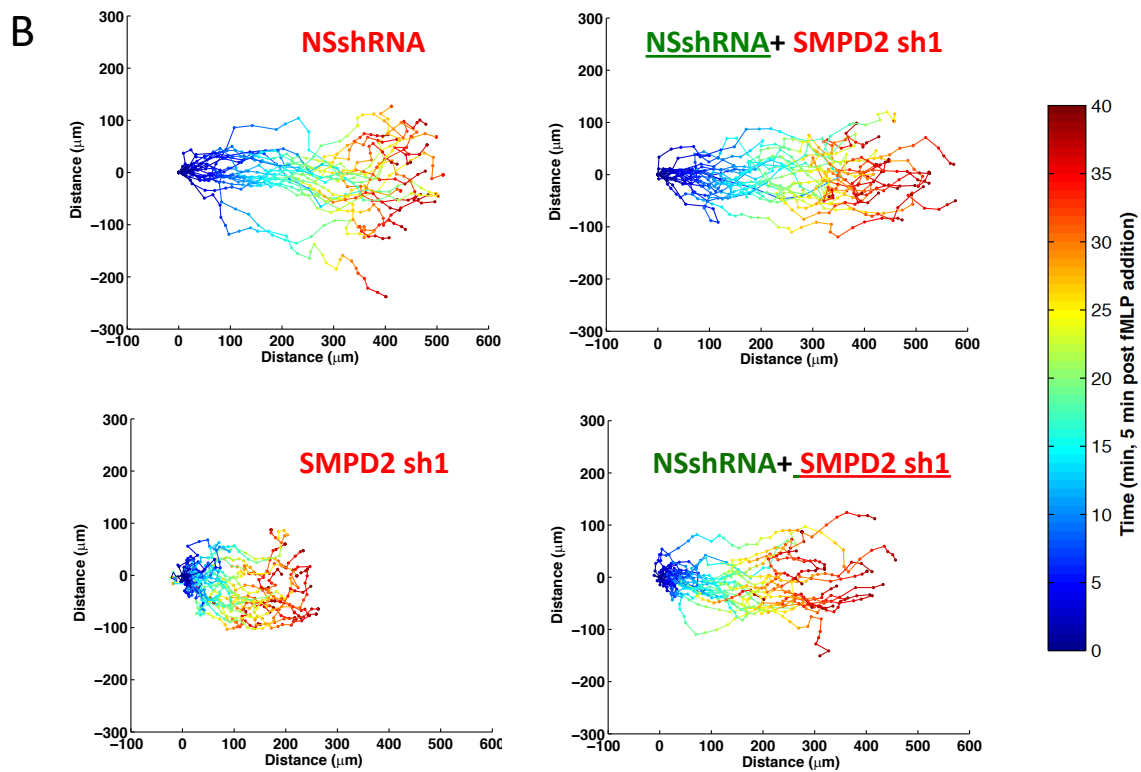

Figure S6

Supplement: S6 Fig — (A) Exosomes purified from DMSO- or GW4869-treated neutrophils were incubated with anti-CD63 antibody-coated Dynabeads (Invitrogen) and detected using CD81-PE antibody in a bead-based flow cytometry assay. Inset: Inhibition was quantified using the relative median fluorescence intensity values from 3 independent experiments. (B) Representative tracks of chemotaxing differentiated PLB-985 cells under agarose towards a 50-pM/μm gradient of fMLP. Top left: NSshRNA cells stained with cytotracker red. Bottom left: SMPD2 sh1 cells stained with cytotracker red. Top right: NSshRNA cells stained with cytotracker green in a mixture of SMPD2 sh1 and NSshRNA cells. Bottom right: SMPD2 sh1 cells stained with cytotracker red in a mixture of SMPD2 sh1 and NSshRNA cells. The temporal location of cells in the X or Y direction is coded according to the colormap. Also see Fig 5A and 5B for further details. Raw data for panels A and B can be found in the Supporting Information section S2 Data file. fMLP, N-formylMethionyl-Leucyl-Phenylalanine; KD, knockdown; NSshRNA, nonspecific shRNA. (PDF) [file pbio.3001271.s006.pdf]

A

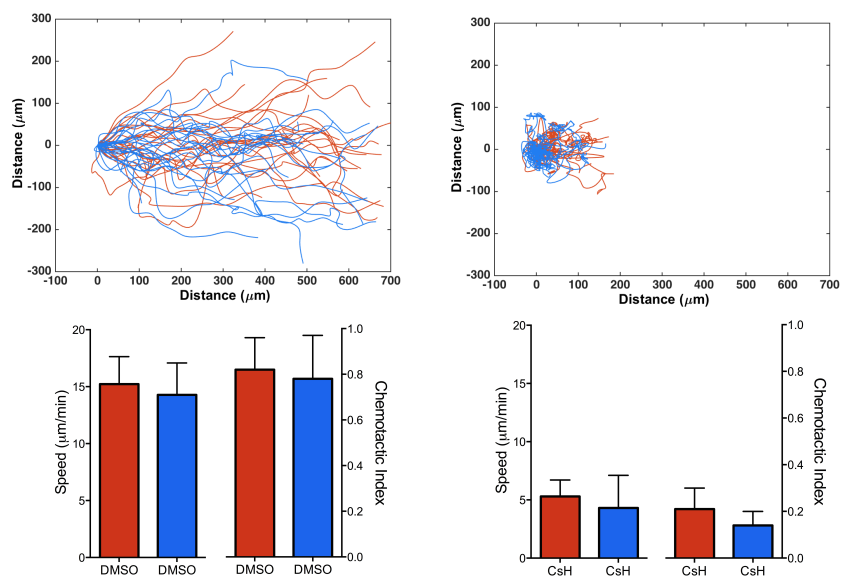

B

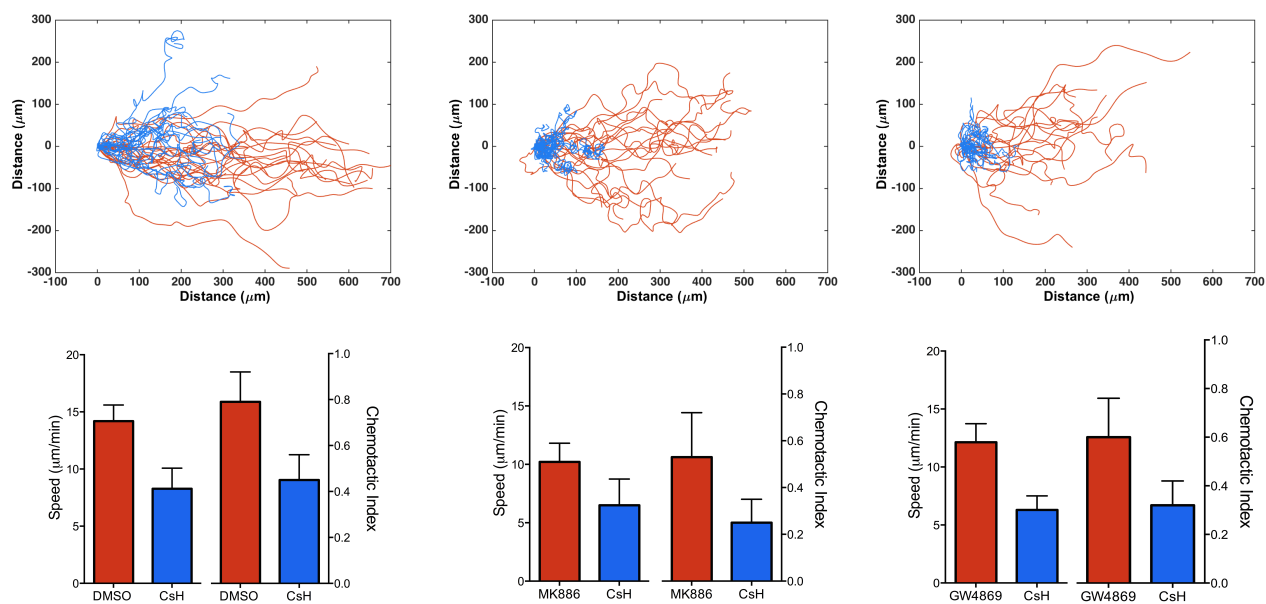

Figure S7

Supplement: S7 Fig — Representative tracks, speed, and CI of mixtures of cytotracker-labeled neutrophils treated with CsH, MK886, or GW4869 migrating under agarose towards an approximately 50 pM/μm gradient of fMLP. Speed and CI were calculated from the tracks of 40 cells and averaging over 4 independent movies. Raw data for panels A and B can be found in the Supporting information section S2 Data file. CI, chemotaxis index; CsH, cyclosporin H; fMLP, N-formylMethionyl-Leucyl-Phenylalanine. (PDF) [file pbio.3001271.s007.pdf]
