# Supplementary material for: Exosomes mediate LTB4 release during neutrophil chemotaxis
Source: PLoS Biol. 2021 Jul 7;19(7):e3001271. doi: 10.1371/journal.pbio.3001271 (PMC8262914; doi:10.1371/journal.pbio.3001271)
Supplement: S1 Raw images — Raw images of western gels for Figs 1C, 2E and 2G, 3D and 3G, 4F, and S4A and S5B are presented. (PDF) [file pbio.3001271.s022.pdf]

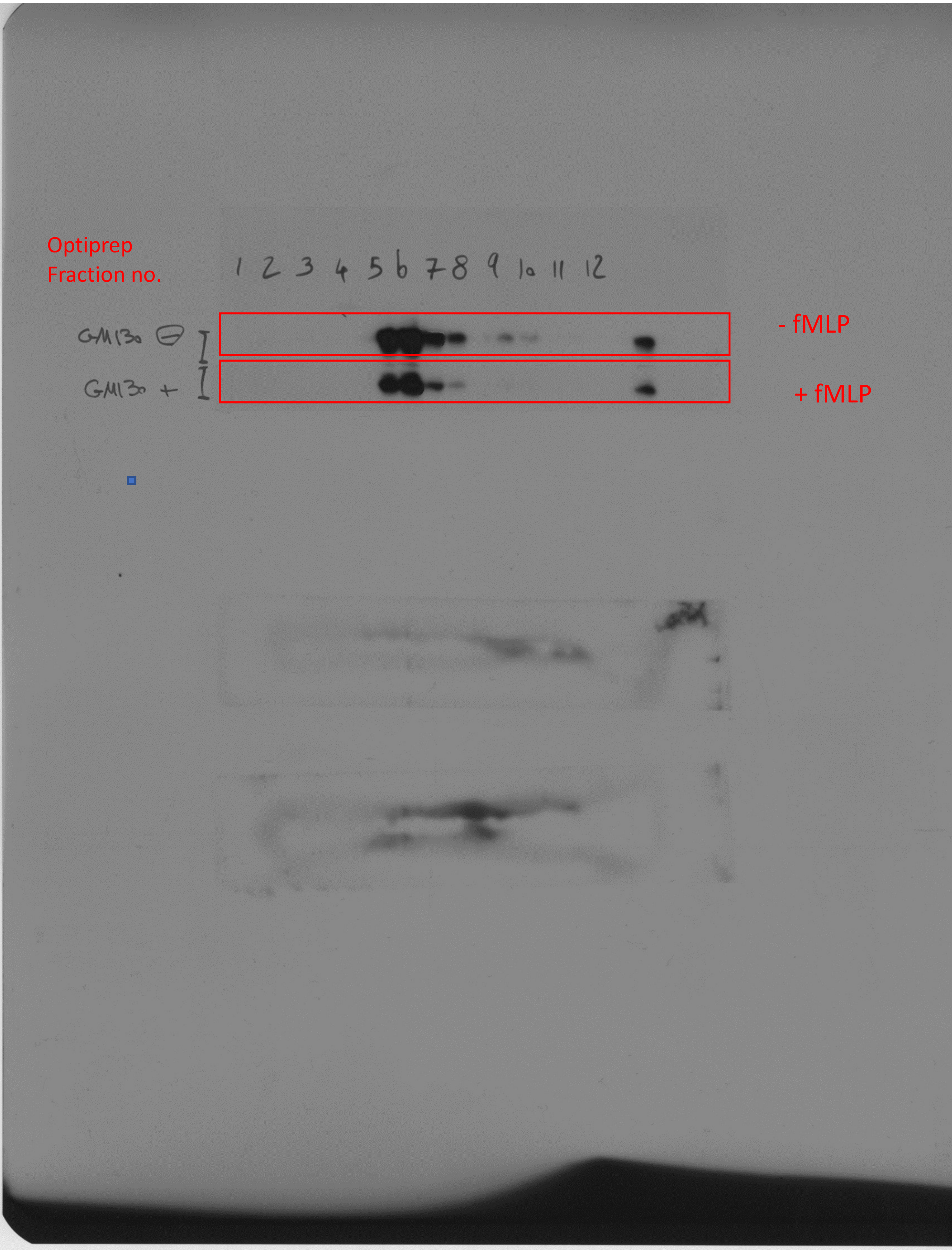


Figure 1C - GM130

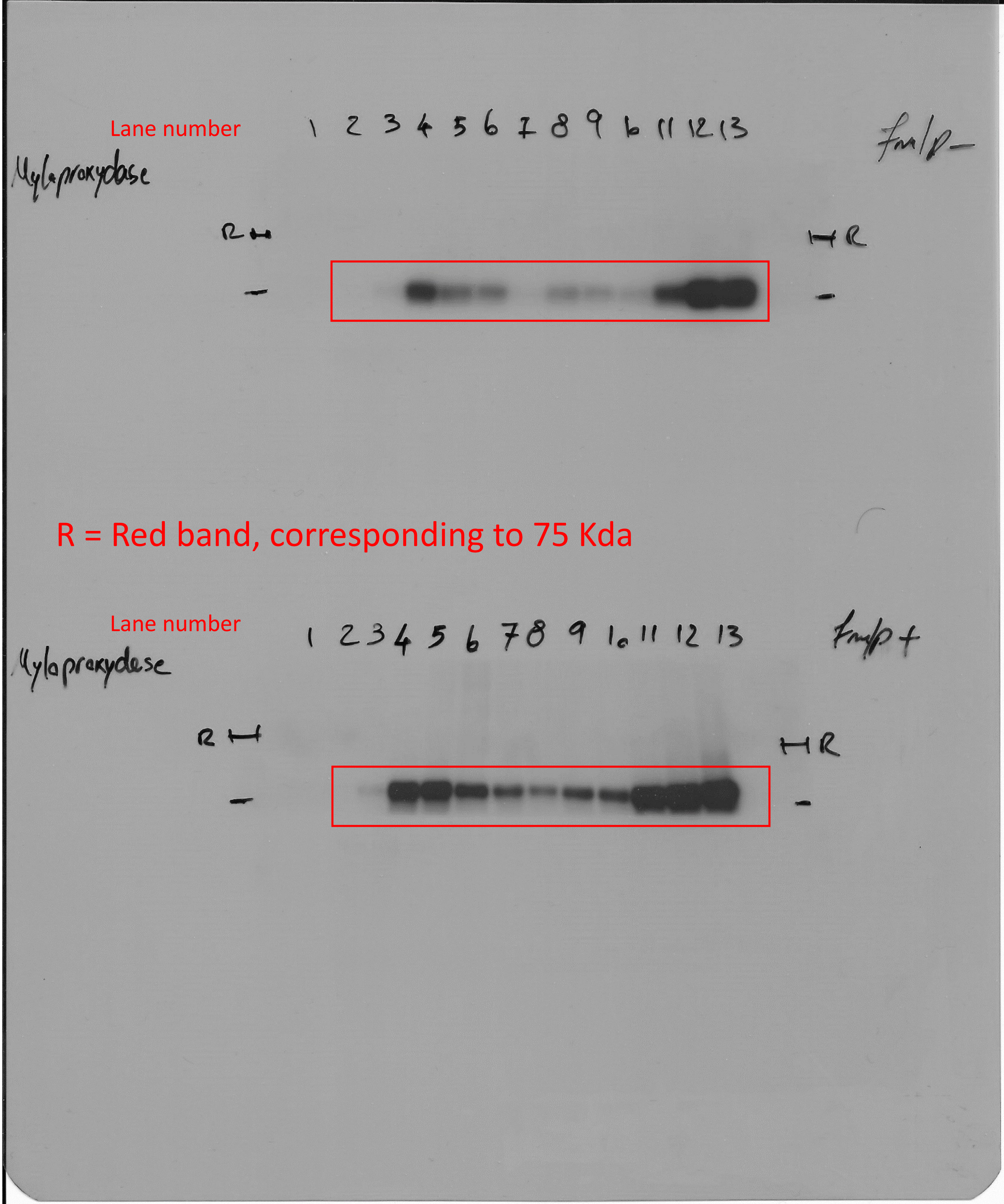


Figure 1C – Myeloperoxidase (MPO)

Note: Lane 1 is fraction 0, Lane 2, Fraction 1 and so forth
Fraction 0 is the supernatant and does not contain gradient media

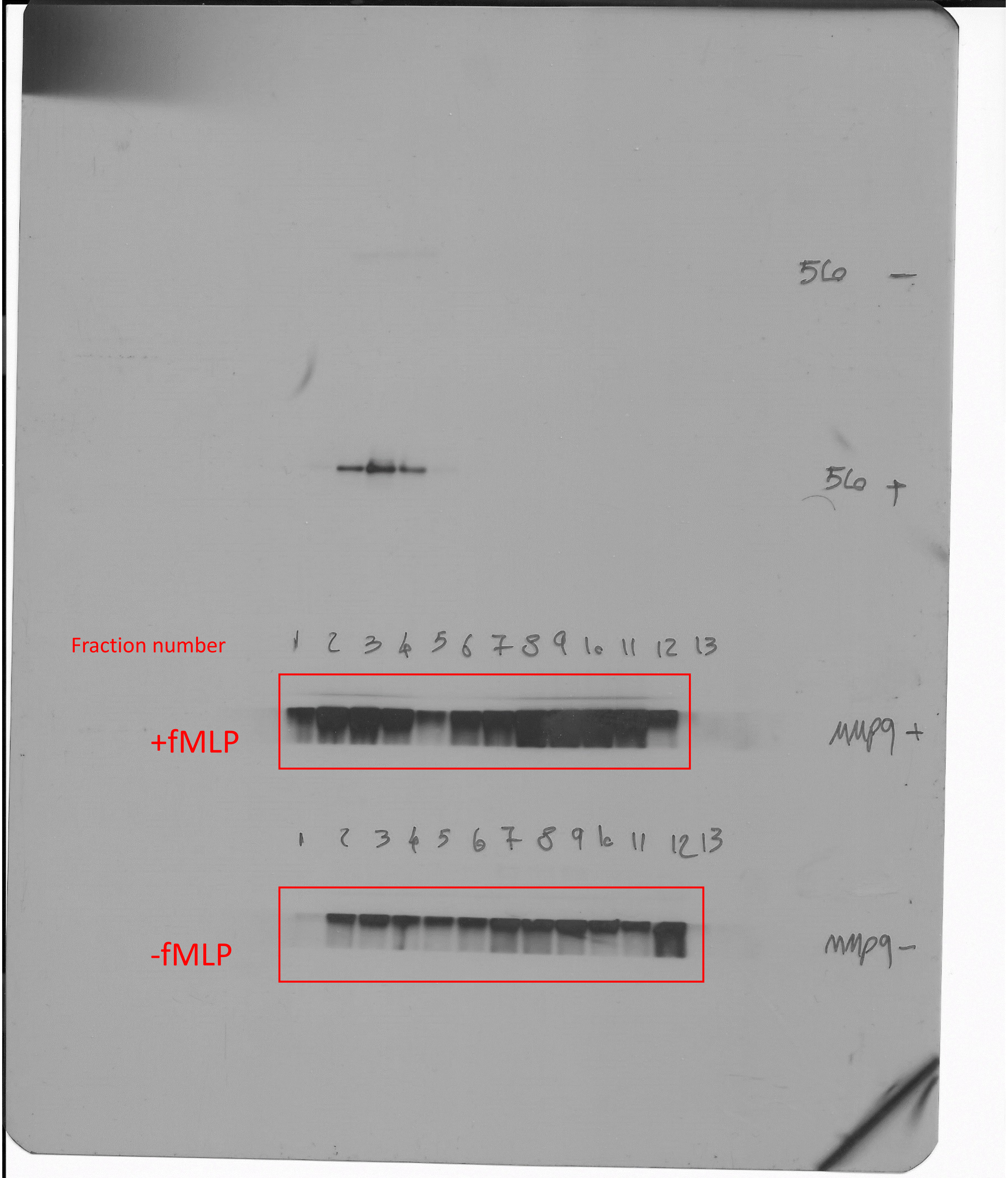


Figure 1C – MMP9

Note: The annotations have been performed on a horizontally flipped western blot. The correct orientations has been included in Fig 1C. The fraction number loading sequence remains unchanged.

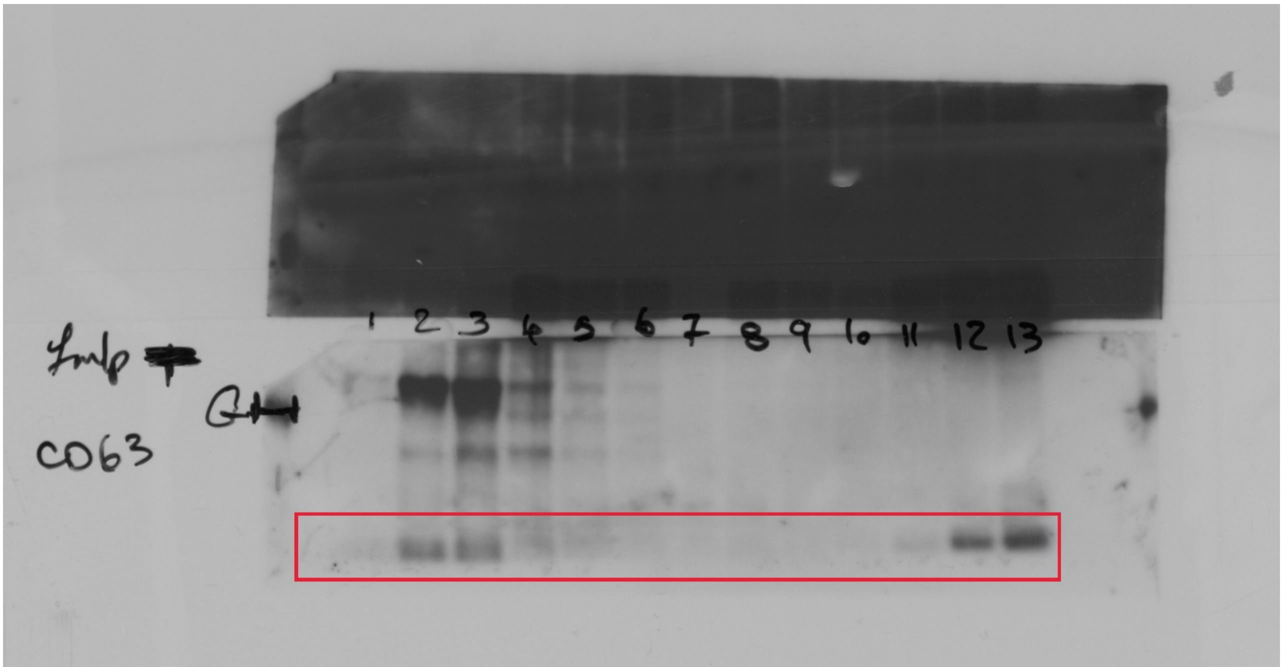


Notes : 1. G- green marker ( 35 Kd, Cd 63 is 25 Kda).
2. Lane 1 = fraction 0, supernatant that does not contain optiprep in the gradient and typically discarded in later experiments

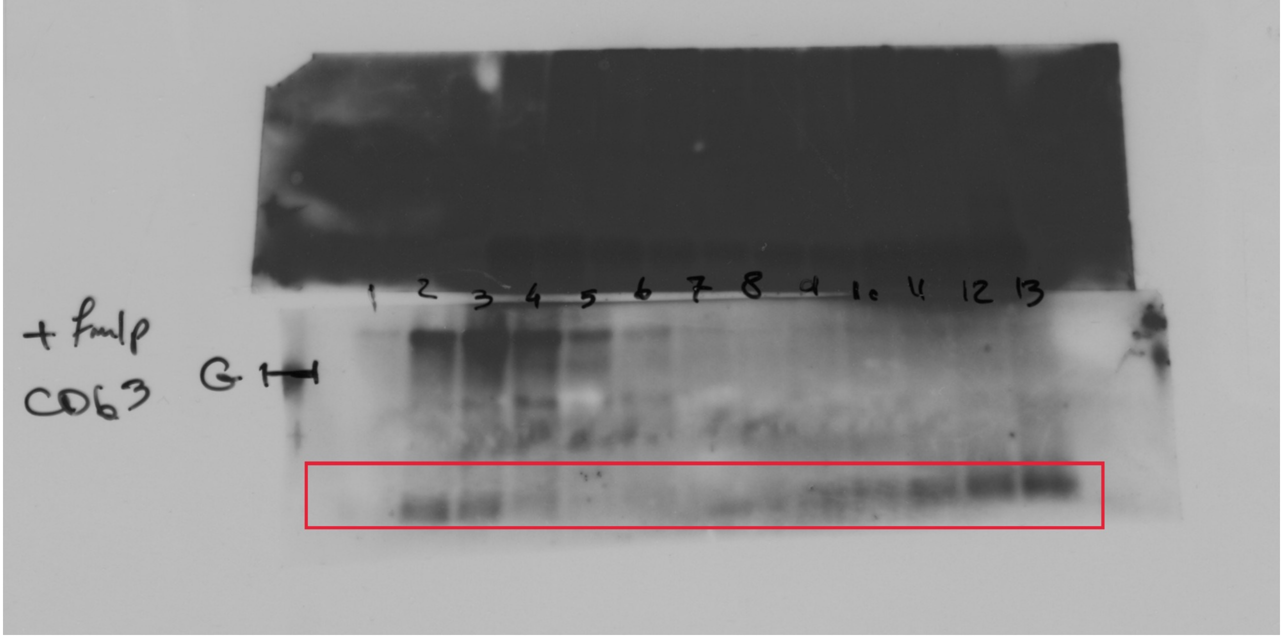


Figure 1C - CD63

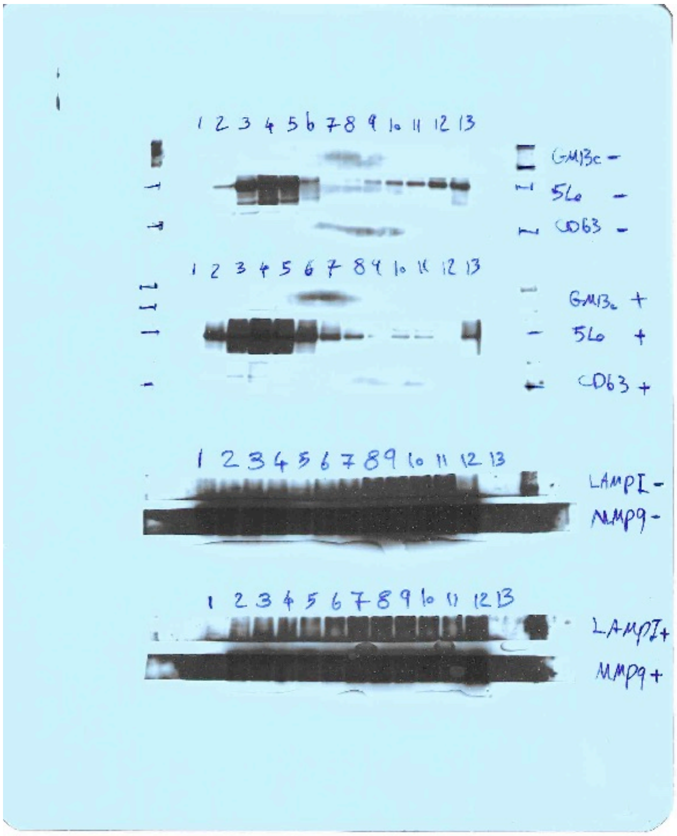


High Exposure

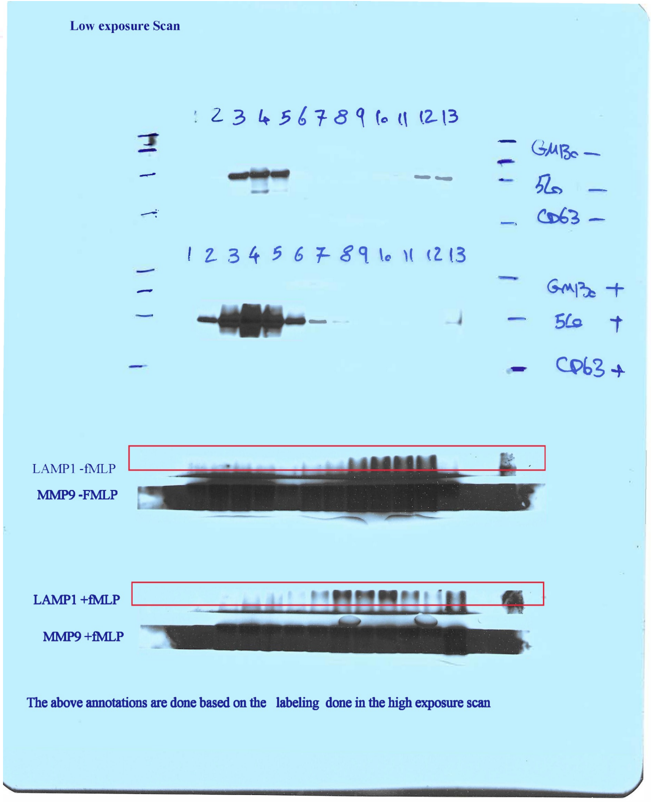


Low Exposure

Figure 1C - LAMP1

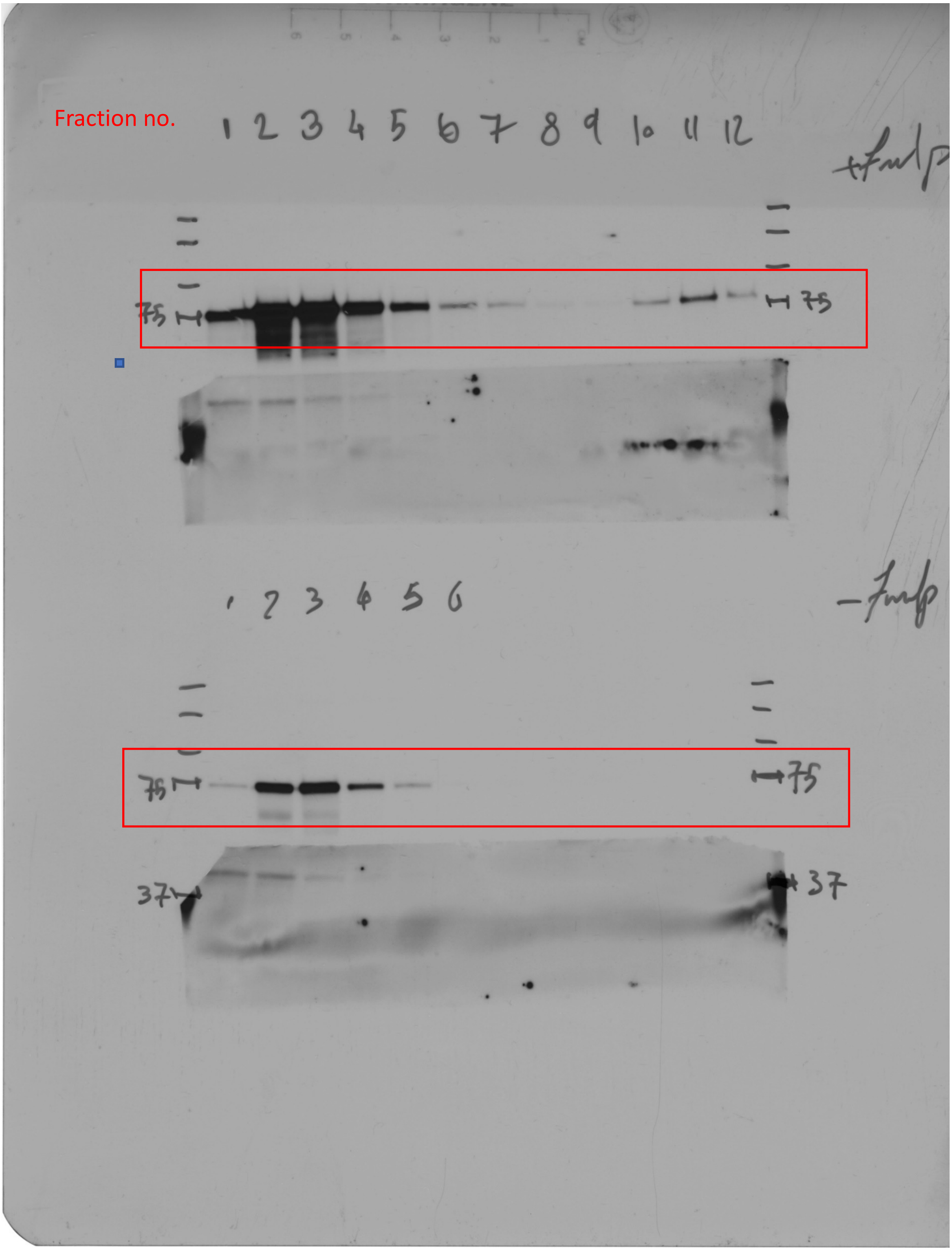


Figure 1C - 5LO

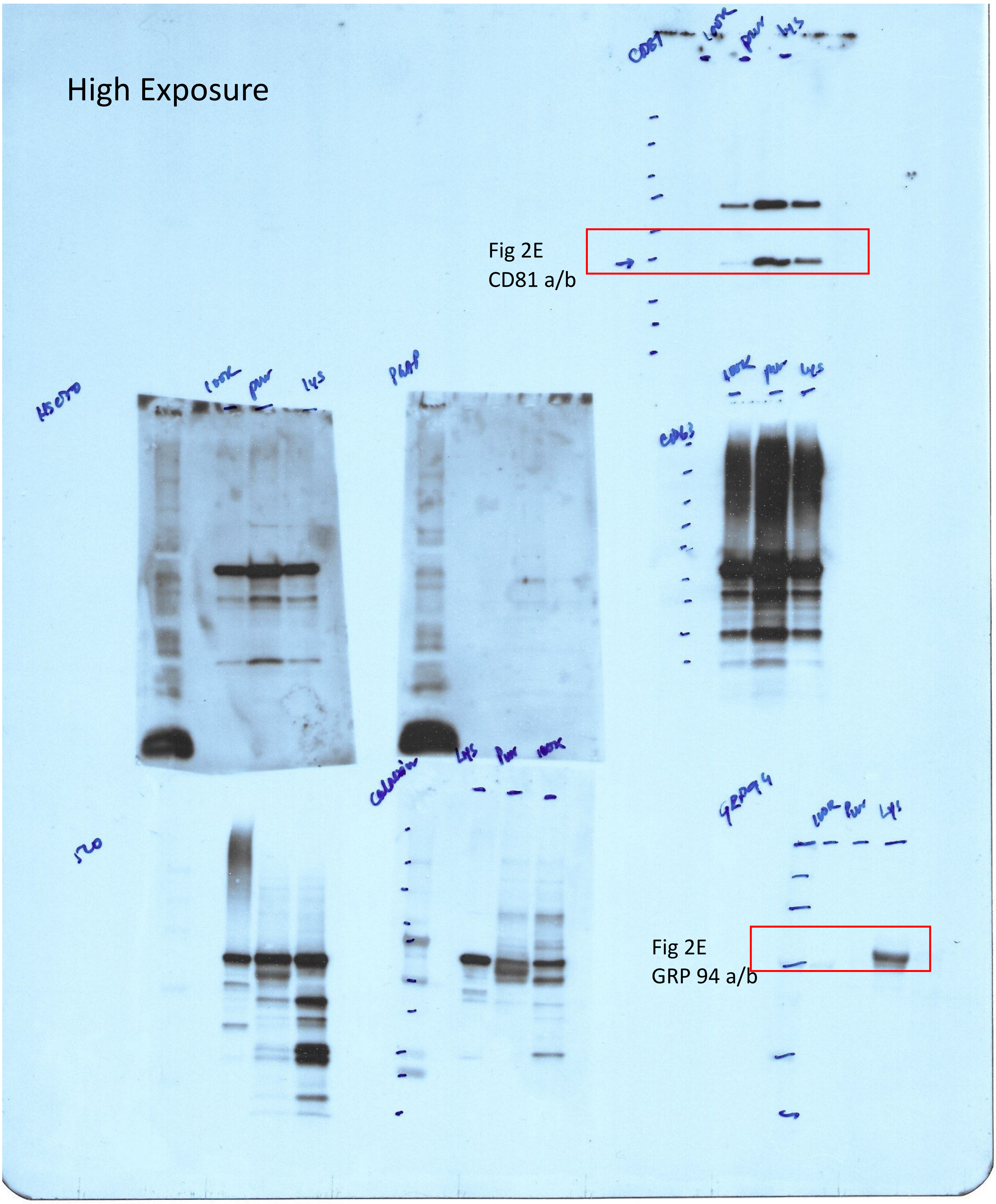


Figure 2E - High exposure Blot
* Also see Fig 2E mid exposure blot

Lys: Lysate
Pur: Purified exosome
100K: Unpurified exosome (100K spin pellet)

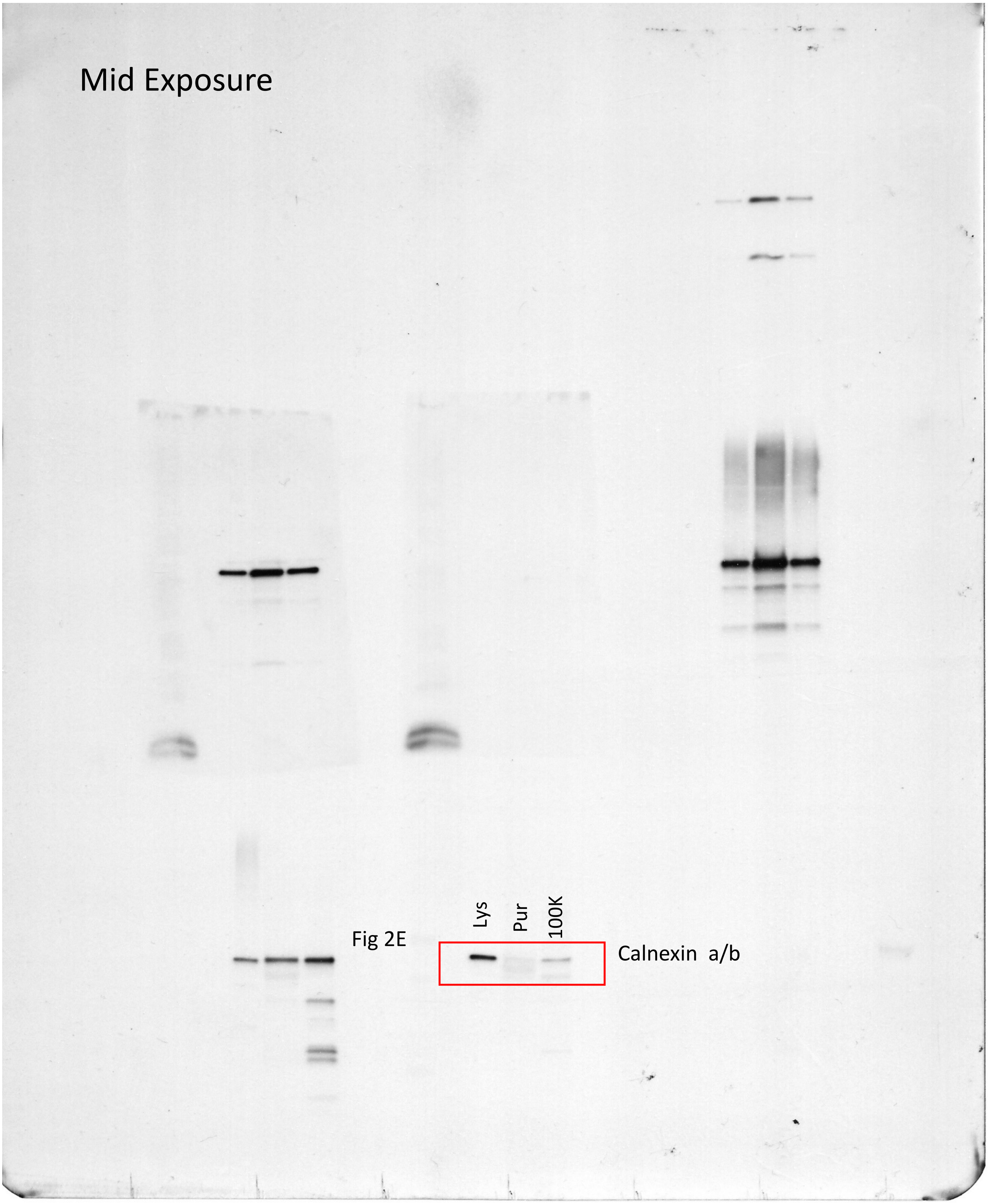


Figure 2E - Mid exposure Blot.
See High exposure blot for annotations

Lys: Lysate
Pur: Purified exosome
100K: Unpurified exosome (100K spin pellet)

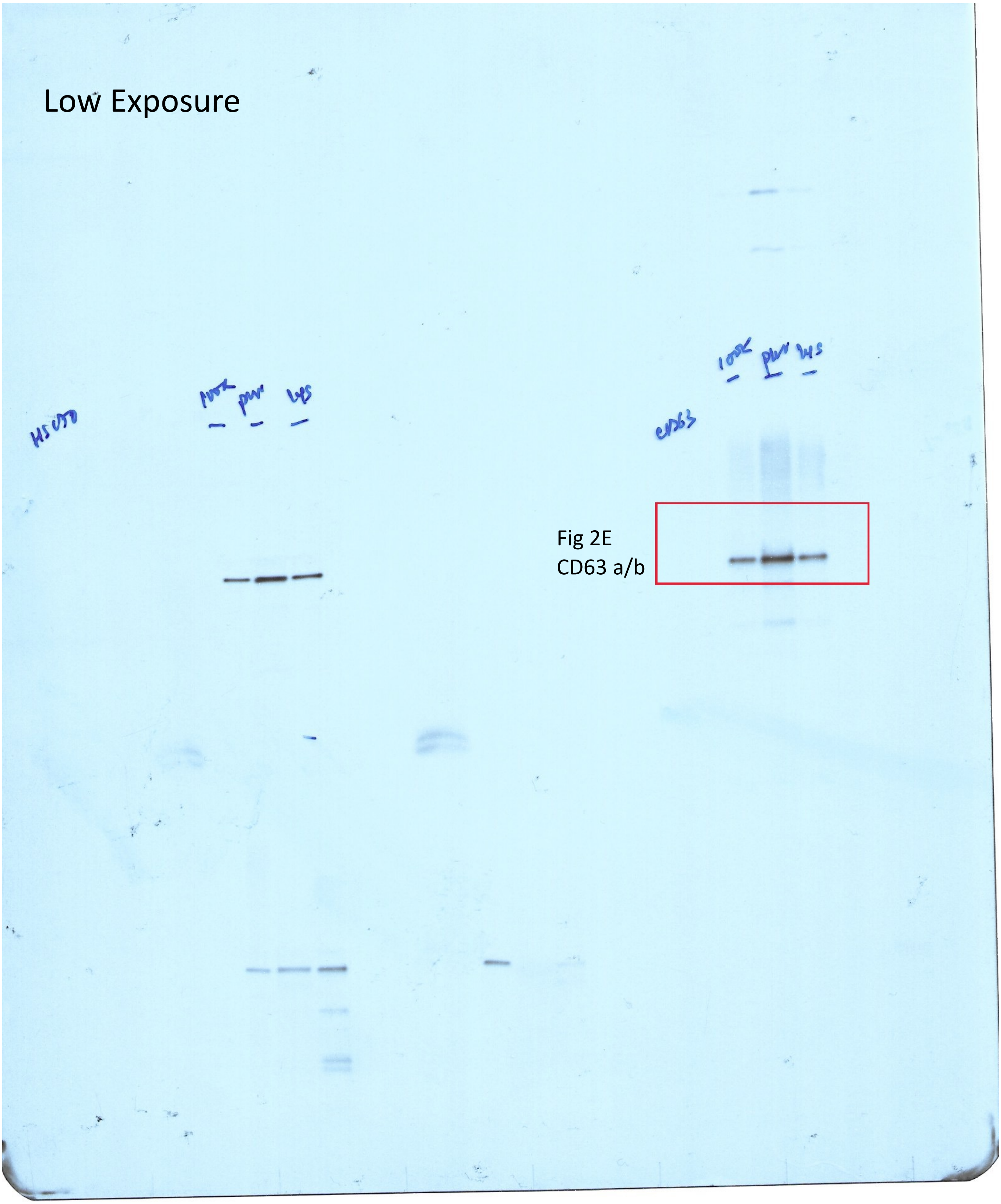


# Figure 2E - Low exposure Blot

Lys: Lysate
Pur: Purified exosome
100K: Unpurified exosome (100K spin pellet)

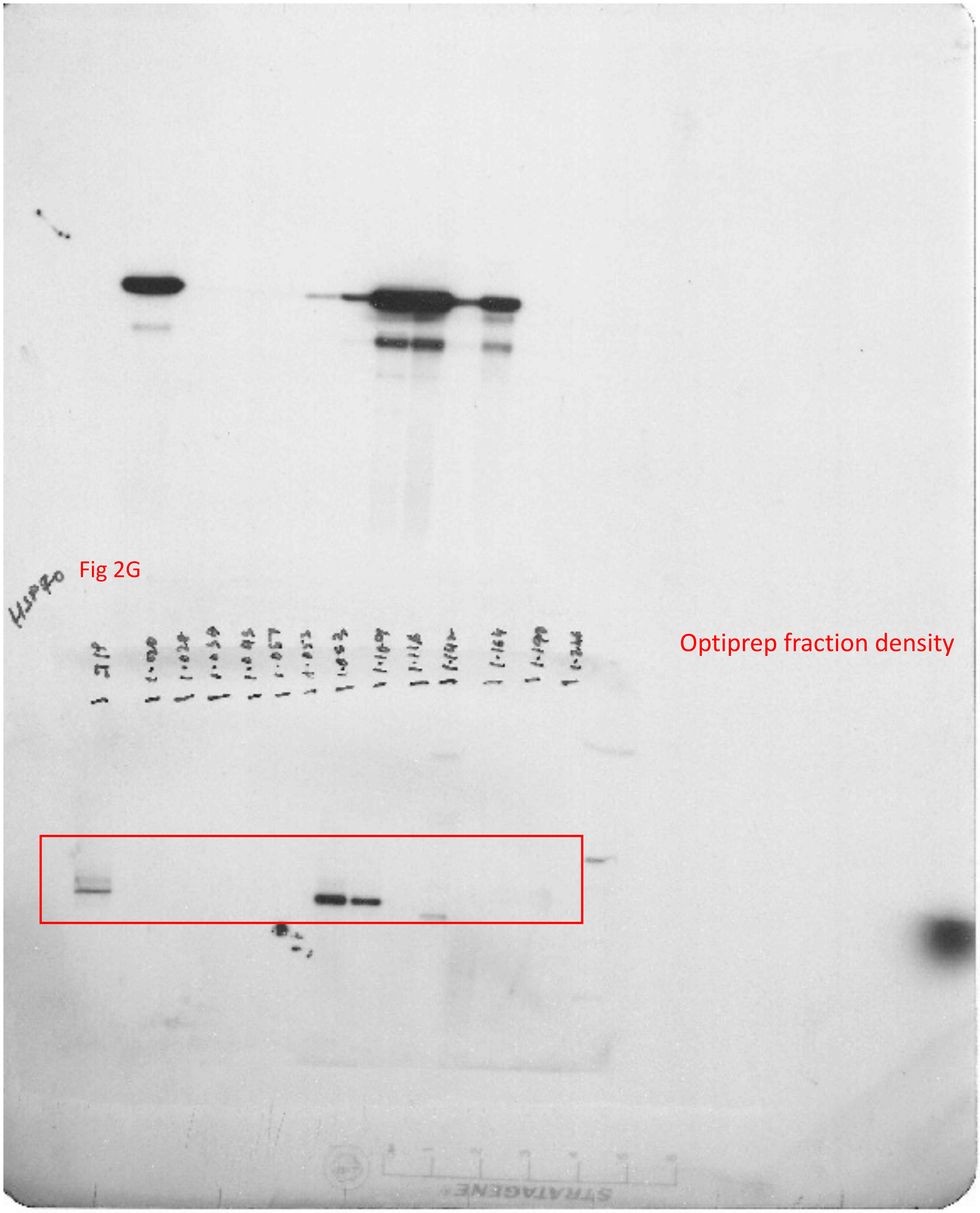


Figure 2G – Hsc70

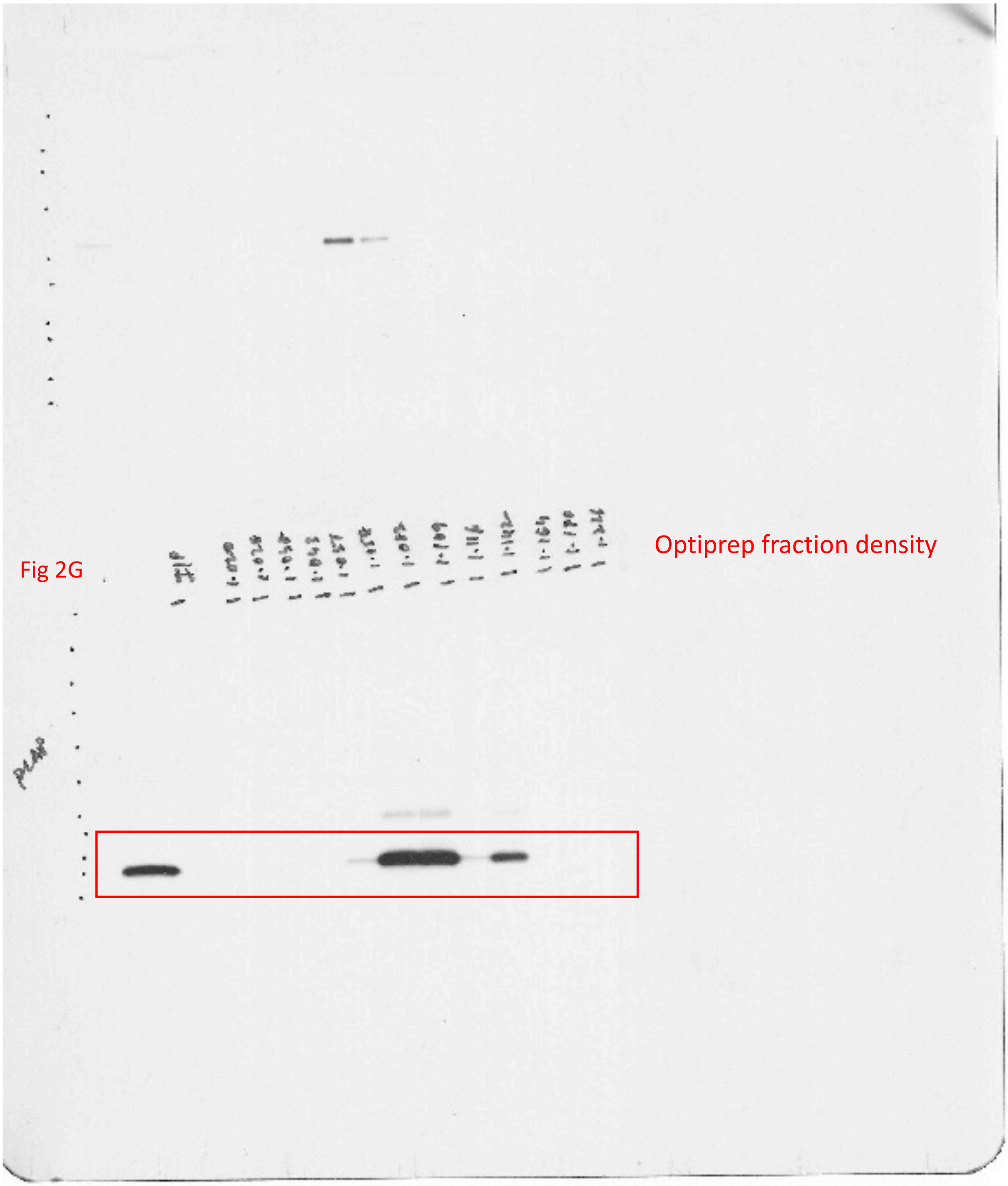


Figure 2G - FLAP

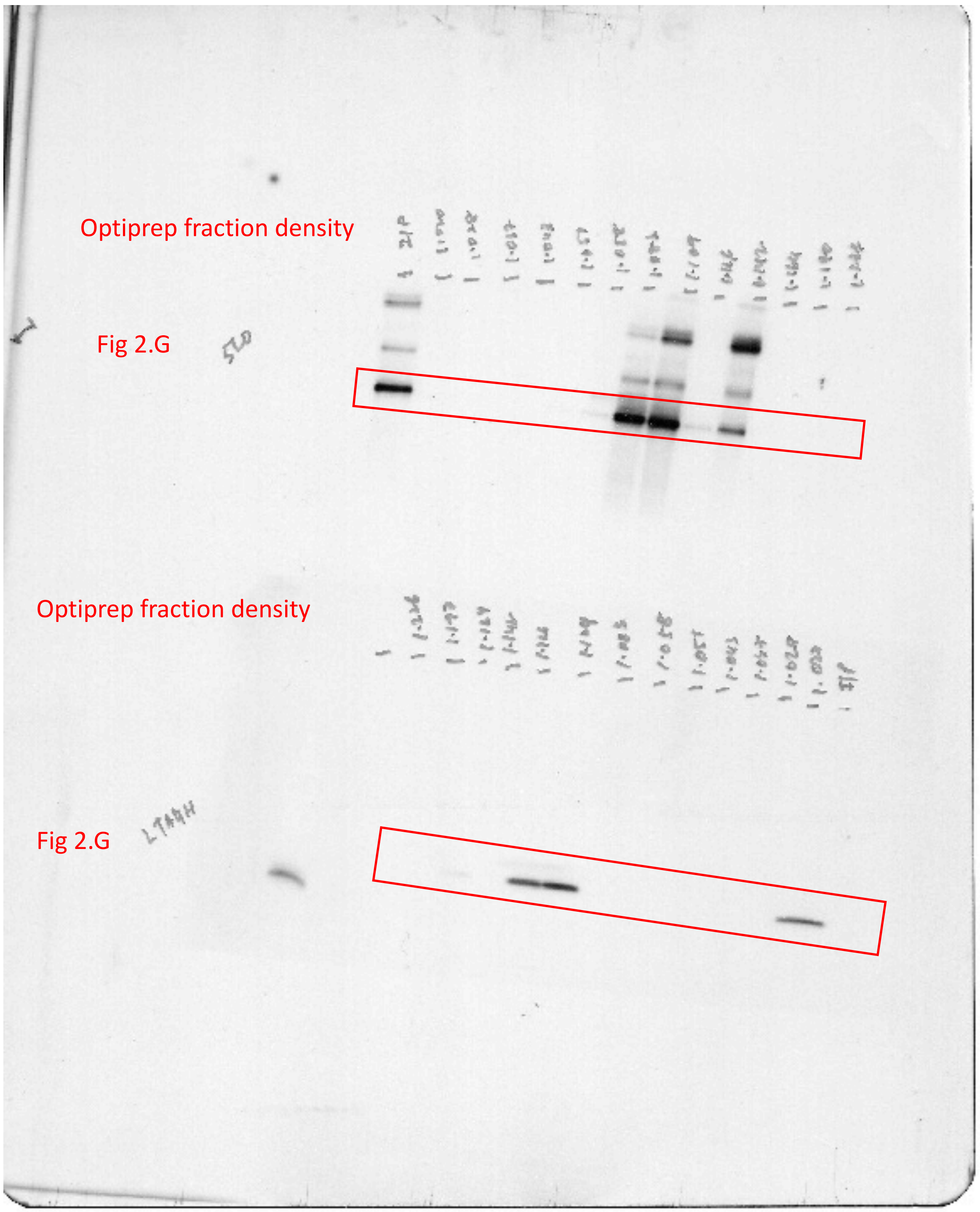


Figure 2G – 5-LO and LTA4H

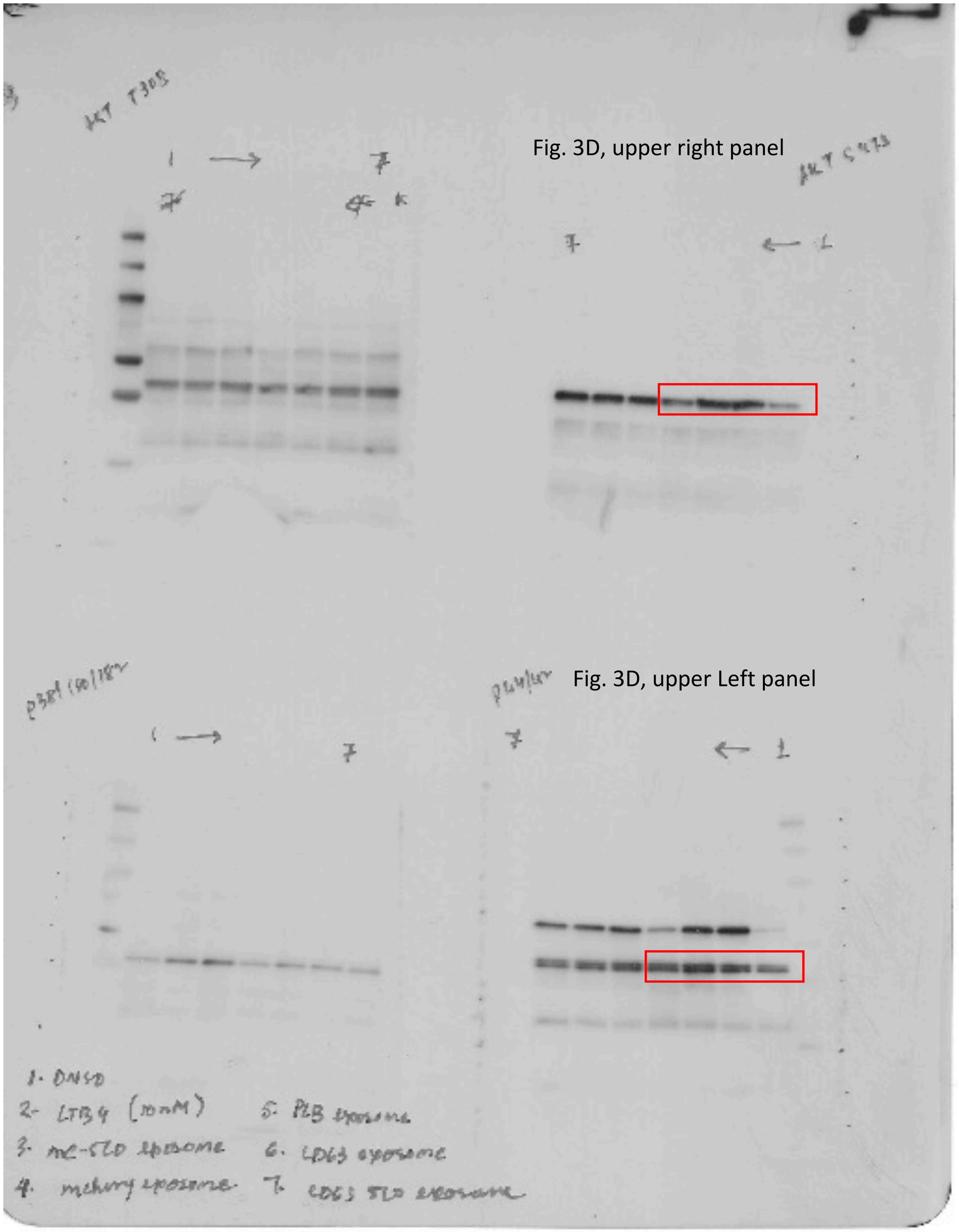


Figure 3D - pERK, pAKT blots

1. DMSO Treated Cells
2. LTB4 treated cells
3. Exosome treated mCherry 5LO cells
4. Exosome from mCherry Cells

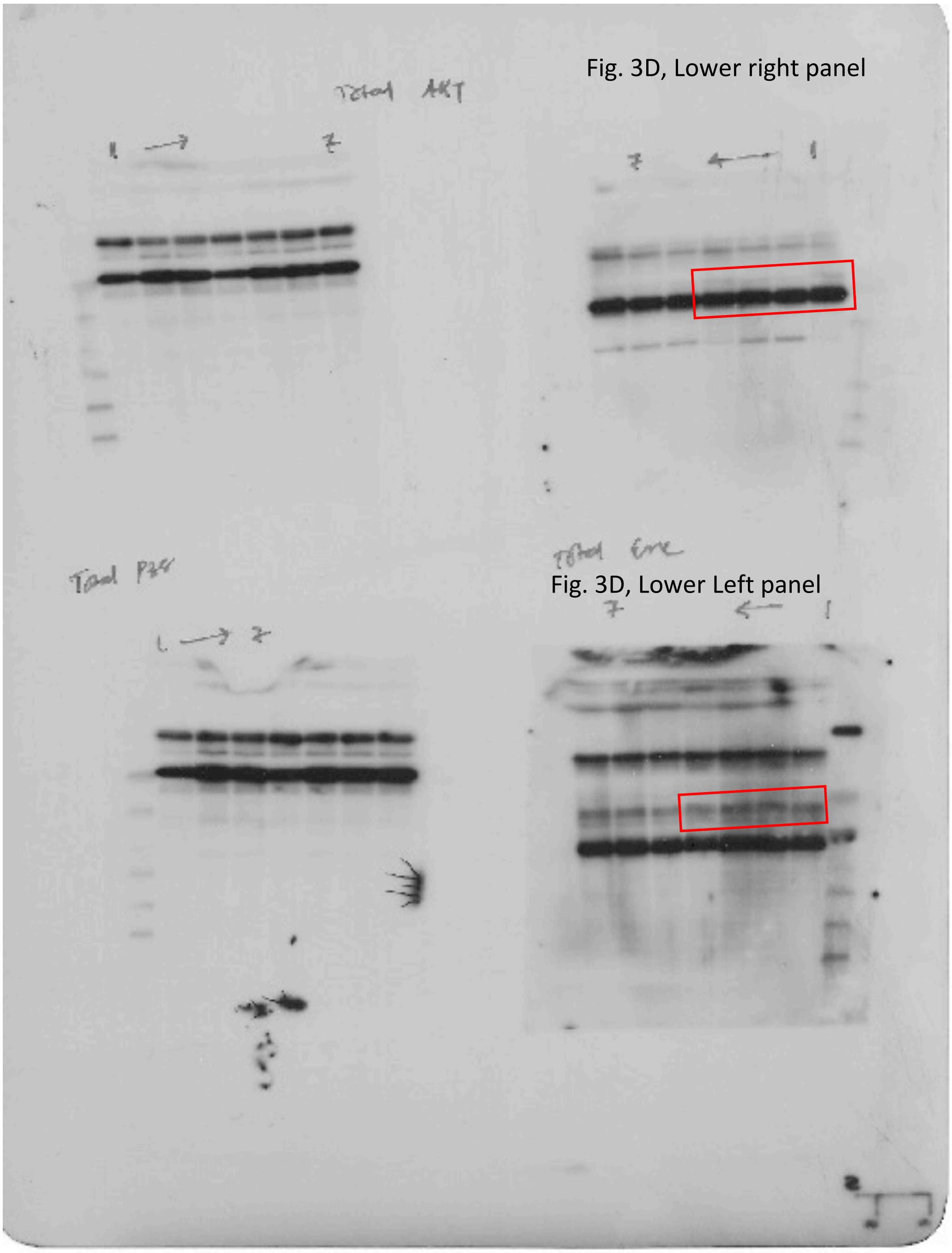


Figure 3D - Total ERK, AKT blots
Annotations same as for Fig 3D pERK/AKT blots

1. DMSO Treated Cells
2. LTB4 treated cells
3. Exosome treated mCherry 5LO cells
4. Exosome from mCherry Cells

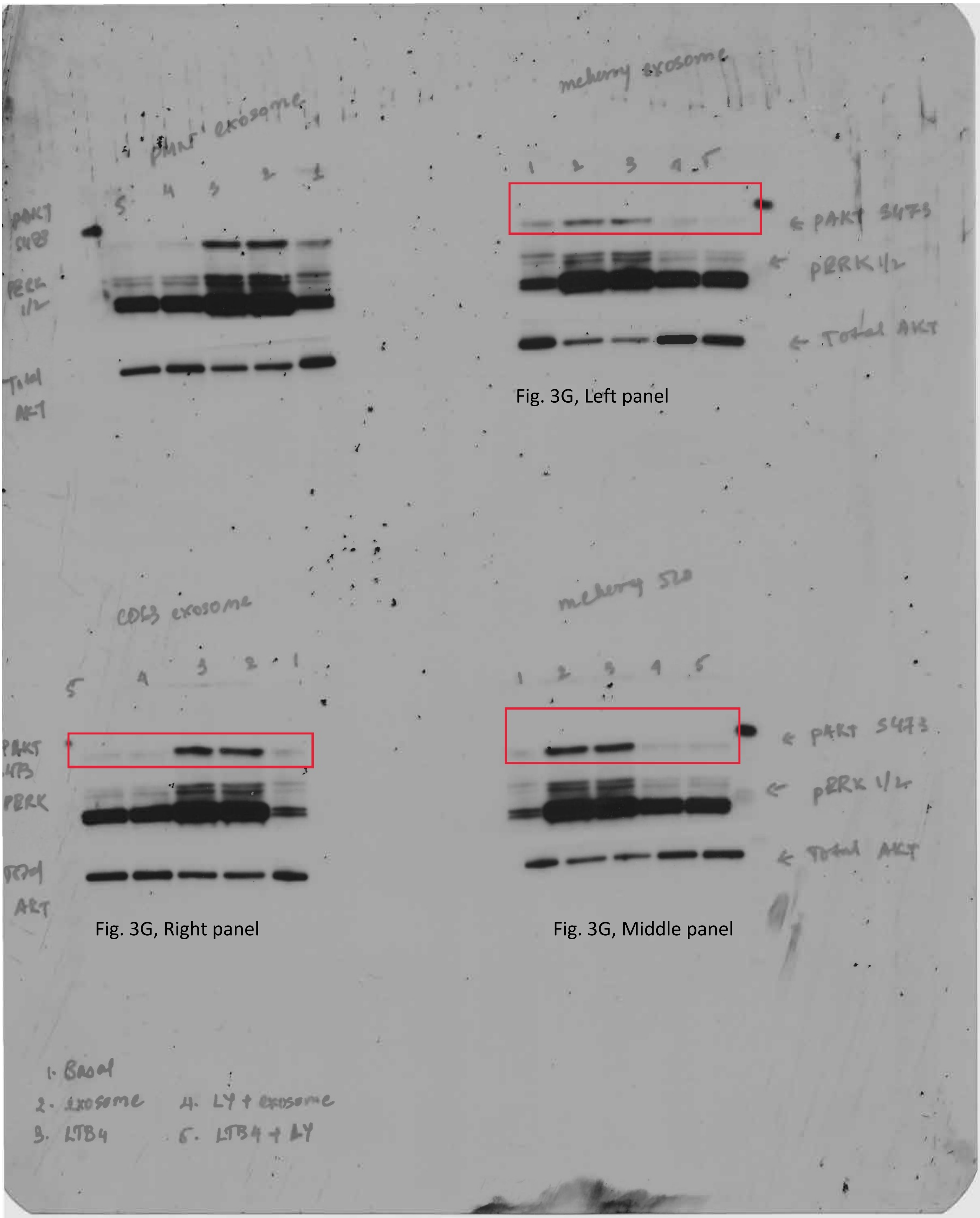


Fig. 3G, Left panel

Fig. 3G, Right panel

Fig. 3G, Middle panel

Figure 3G

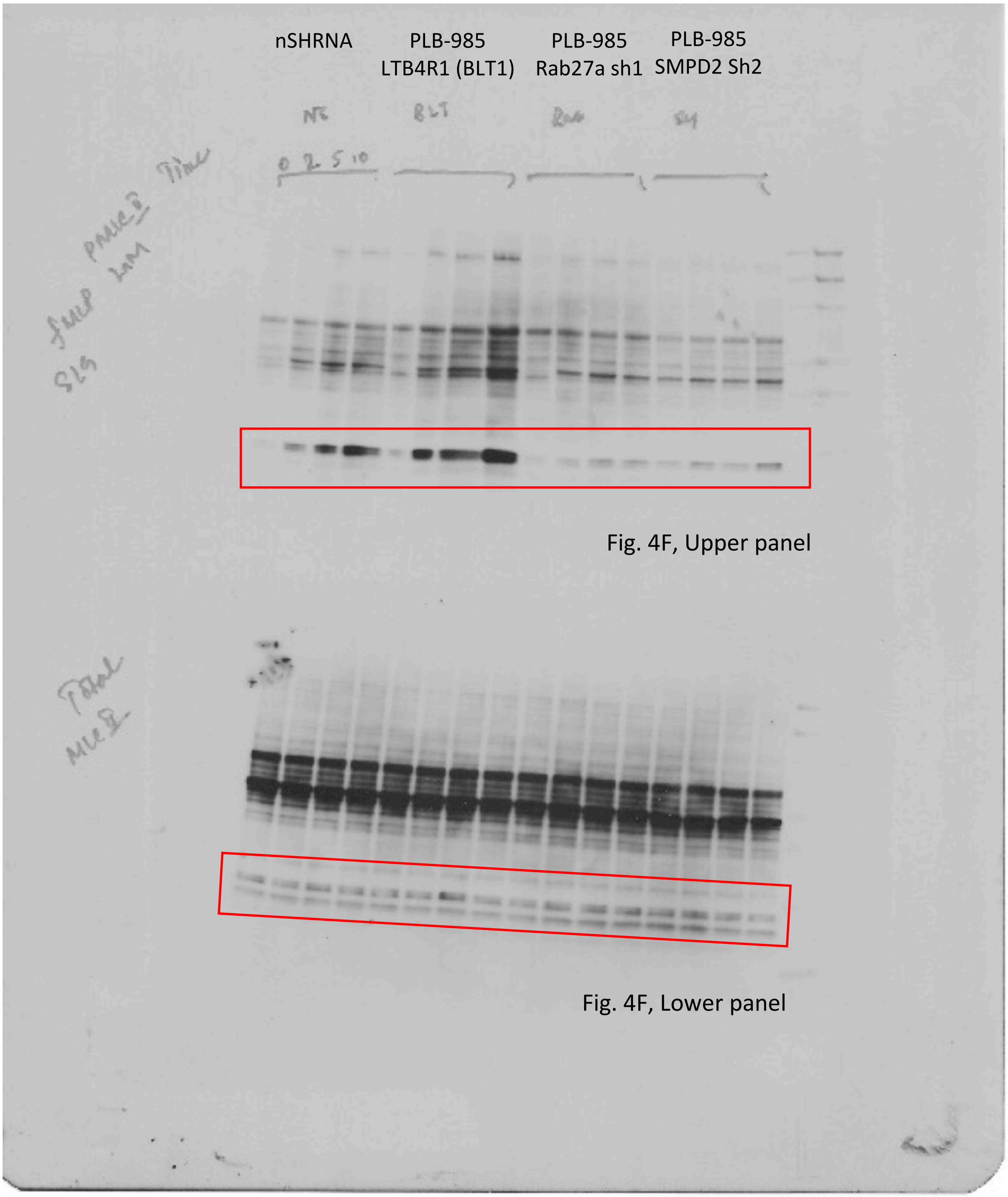


Fig. 4F, Upper panel

Fig. 4F, Lower panel

Figure 4F

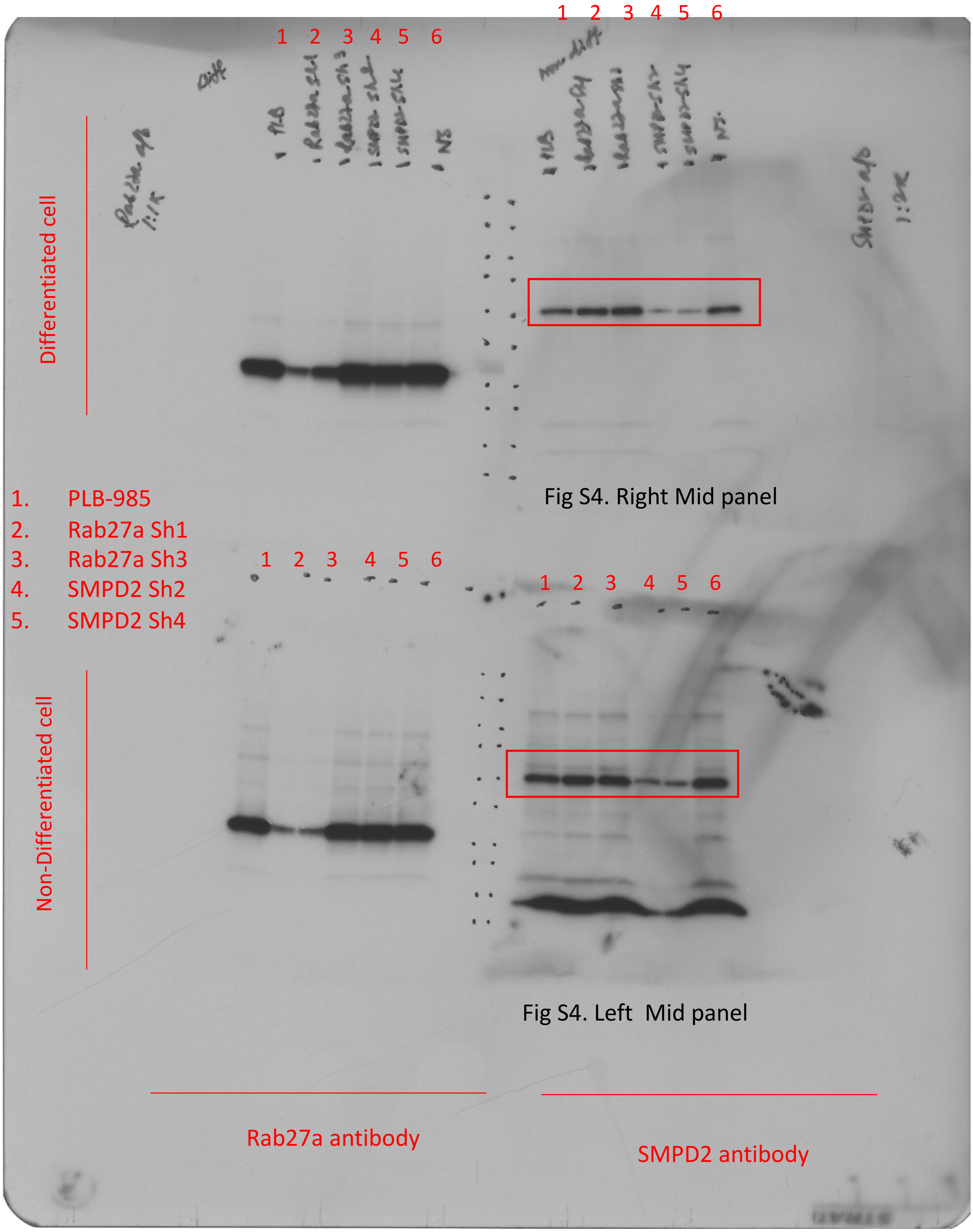


Figure S4A- High exposure

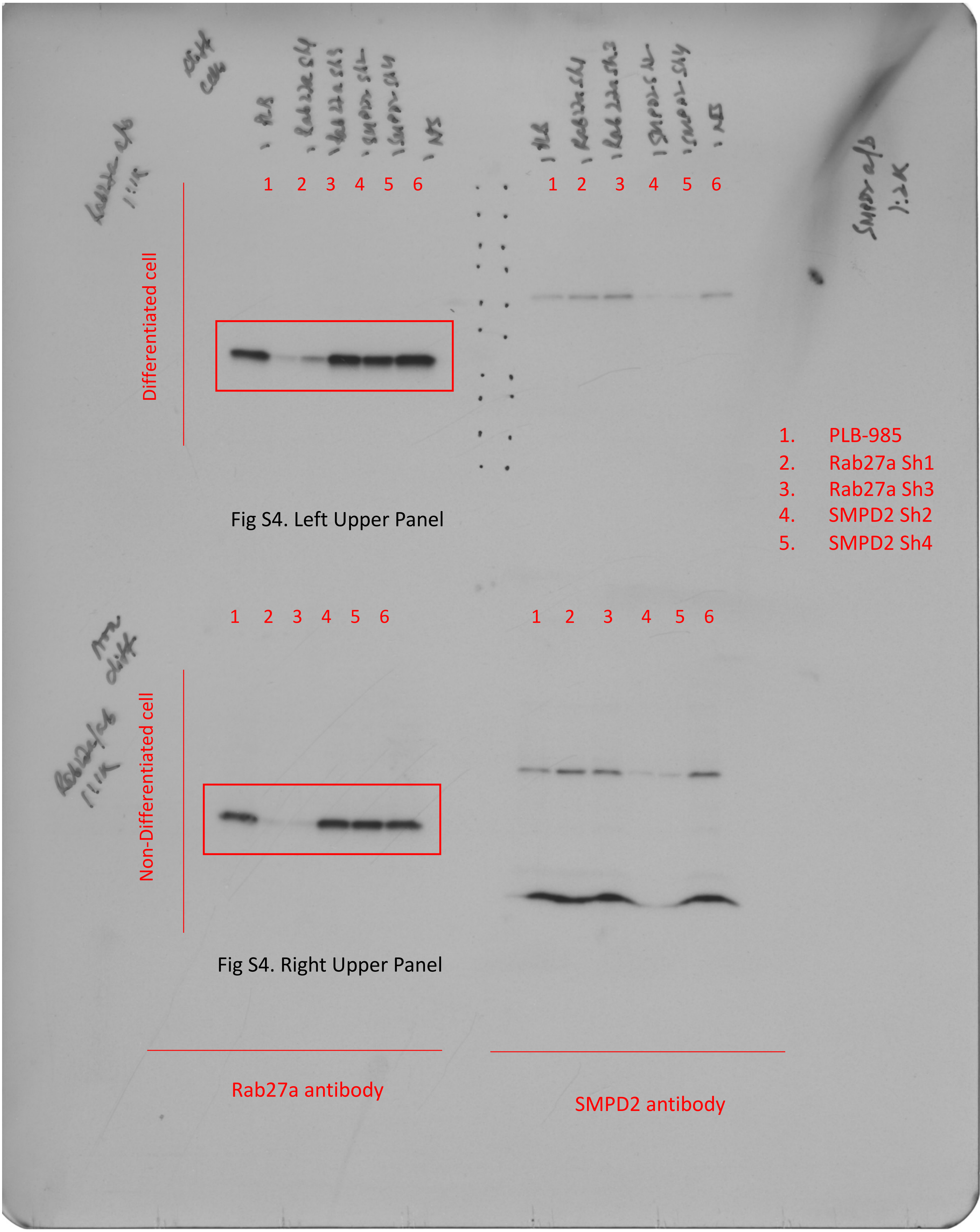


Figure S4A - Low exposure

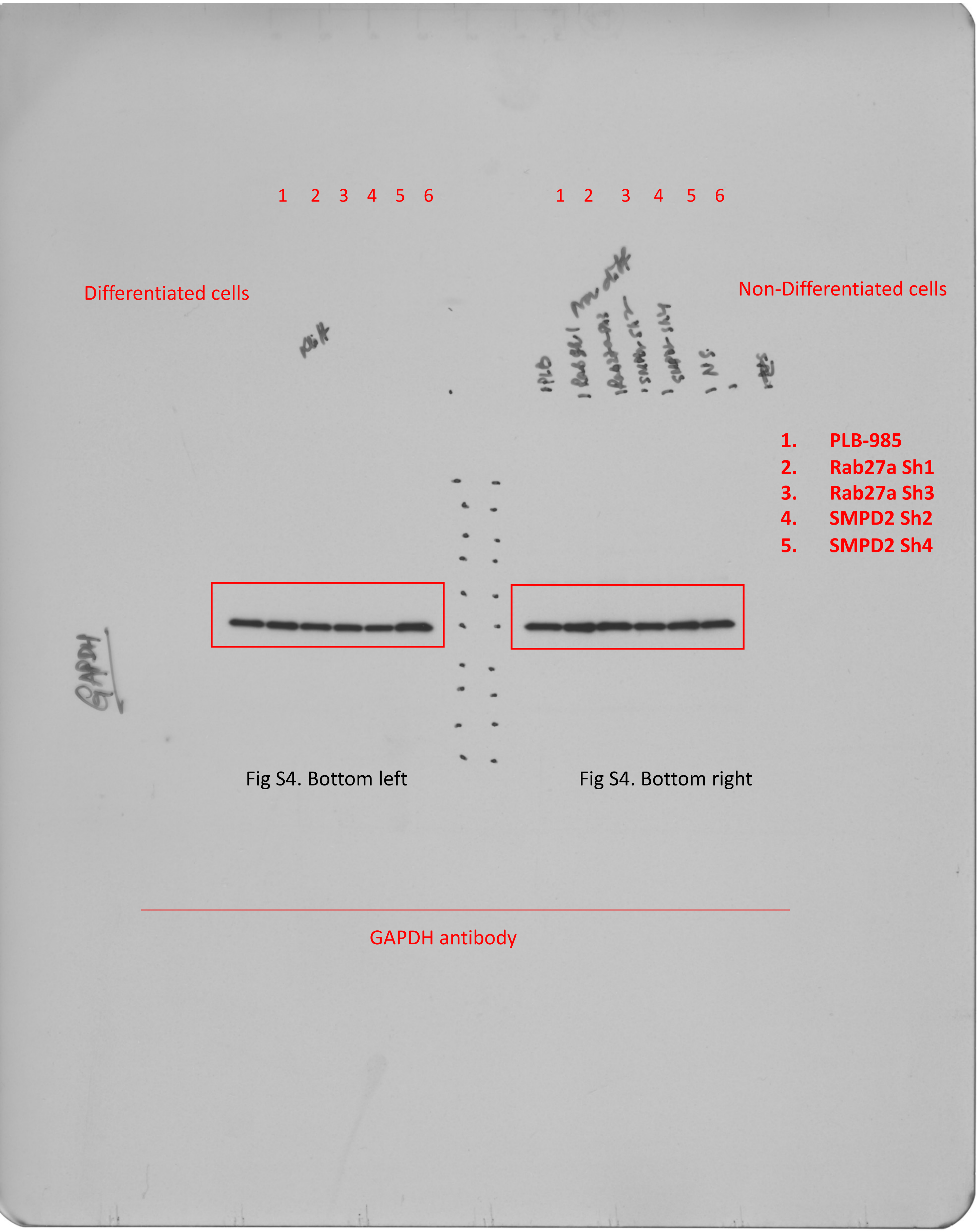
1 2 3 4 5 6
1 2 3 4 5 6
Differentiated cells
Non-Differentiated cells
1. PLB-985
2. Rab27a Sh1
3. Rab27a Sh3
4. SMPD2 Sh2
5. SMPD2 Sh4
Fig S4. Bottom left
Fig S4. Bottom right
GAPDH antibody

Figure S4A

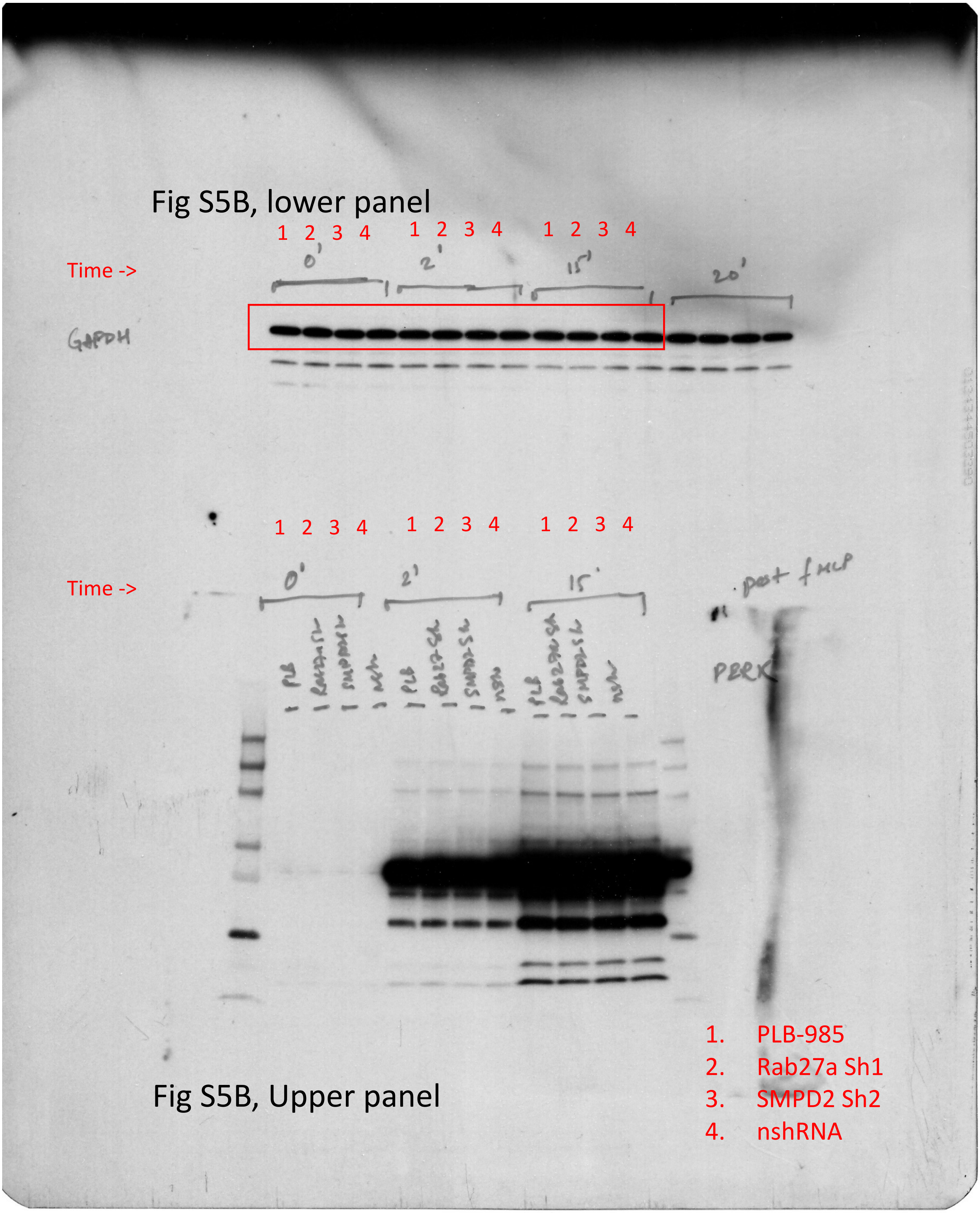


Figure S5B - High exposure

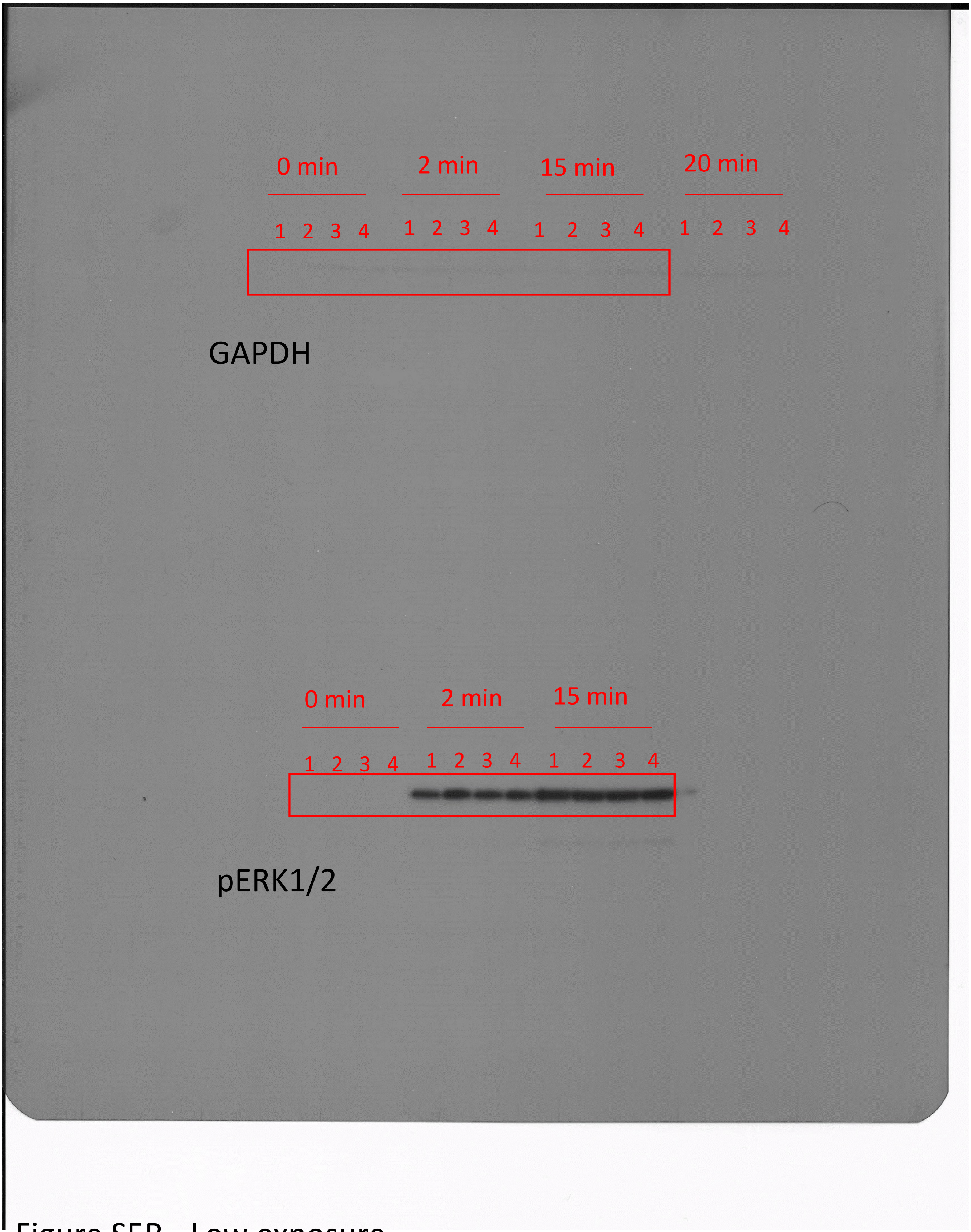


Figure S5B - Low exposure

See Fig S5B High exposure for annotations
